# A Burst of miRNA Innovation in the Early Evolution of Butterflies and Moths

**DOI:** 10.1093/molbev/msv004

**Published:** 2015-01-08

**Authors:** Shan Quah, Jerome H.L. Hui, Peter W.H. Holland

**Affiliations:** ^1^Department of Zoology, University of Oxford; ^2^School of Life Sciences and The State Key Laboratory of Agrobiotechnology, The Chinese University of Hong Kong, Shatin, Hong Kong

**Keywords:** microRNA, Lepidoptera, segmentation, wing patterning, Gli, homeobox

## Abstract

MicroRNAs (miRNAs) are involved in posttranscriptional regulation of gene expression. Because several miRNAs are known to affect the stability or translation of developmental regulatory genes, the origin of novel miRNAs may have contributed to the evolution of developmental processes and morphology. Lepidoptera (butterflies and moths) is a species-rich clade with a well-established phylogeny and abundant genomic resources, thereby representing an ideal system in which to study miRNA evolution. We sequenced small RNA libraries from developmental stages of two divergent lepidopterans, *Cameraria ohridella* (Horse chestnut Leafminer) and *Pararge aegeria* (Speckled Wood butterfly), discovering 90 and 81 conserved miRNAs, respectively, and many species-specific miRNA sequences. Mapping miRNAs onto the lepidopteran phylogeny reveals rapid miRNA turnover and an episode of miRNA fixation early in lepidopteran evolution, implying that miRNA acquisition accompanied the early radiation of the Lepidoptera. One lepidopteran-specific miRNA gene, miR-2768, is located within an intron of the homeobox gene *invected*, involved in insect segmental and wing patterning. We identified *cubitus interruptus* (*ci*) as a likely direct target of miR-2768, and validated this suppression using a luciferase assay system. We propose a model by which miR-2768 modulates expression of *ci* in the segmentation pathway and in patterning of lepidopteran wing primordia.

## Introduction

During development, the information in an organism’s genome is used to coordinate the generation of morphology. Evolutionary modification of this developmental process in different animal lineages has given rise to the vast diversity of body form and function observed across the Metazoa today. Many evolutionary changes to genes have been documented including gene duplications (Brooke et al. 1998; Putnam et al. 2008), coding sequence alterations (Fondon and Garner 2004), and modifications to gene expression (Jeong et al. 2006). Although much focus has been on the evolution of *cis*-regulatory elements that affect gene expression (Putnam et al. 2008; Chan et al. 2010), there are also *trans*-acting factors that should not be overlooked. In particular, there is emerging evidence that evolutionary changes to microRNAs (miRNAs) could have a role to play in the evolution of development (Griffiths-Jones et al. 2011; Hui et al. 2013; Moran et al. 2014; Ninova et al. 2014).

miRNAs are short (∼22 nt), noncoding RNAs initially transcribed as longer primary transcripts. Each primary transcript includes one or more imperfect hairpins of approximately 80 nt that are excised in the nucleus to give rise to precursor miRNAs. These are exported to the cytoplasm and undergo further processing prior to being incorporated in the RNA-induced silencing complex (RISC). The mature miRNA then functions to guide RISC to specific messenger RNA transcripts to bring about downregulation of gene expression, by binding to specific target sequences primarily in the 3′-untranslated regions (3′-UTRs) of protein-coding genes (Kenny et al. 2013). Several miRNAs are known to target genes involved in developmental processes (Yekta et al. 2004; Moran et al. 2014), and a general role in stabilization of developmental pathways in the face of fluctuating environmental conditions has also been proposed (Peterson et al. 2009; Herranz and Cohen 2010).

The miRNA repertoire in a given evolutionary lineage is constantly shifting under the opposing influences of miRNA birth and miRNA death (the gain and loss of miRNA genes). Previous work in vertebrates (Heimberg et al. 2008), mammals (Meunier et al. 2013), drosophilids (Lu et al. 2008; Nozawa et al. 2010; Lyu et al. 2014), and flatworms (Fromm et al. 2013) have suggested that miRNA birth rates can differ substantially in different lineages, while loss is thought to be less frequent than gain. For example, there was a burst of miRNA innovation on the stem lineage leading to the vertebrates following divergence from other chordates, and this has been postulated to be a contributory factor in the evolution of vertebrate complexity (Heimberg et al. 2008). Endoparasitic platyhelminths have undergone significant losses of conserved miRNA families which have not been observed to the same extent in other bilaterian phyla studied. This is proposed to be correlated with the evolution of reduced morphological and metabolic complexity (Fromm et al. 2013).

**Fig. 1.**
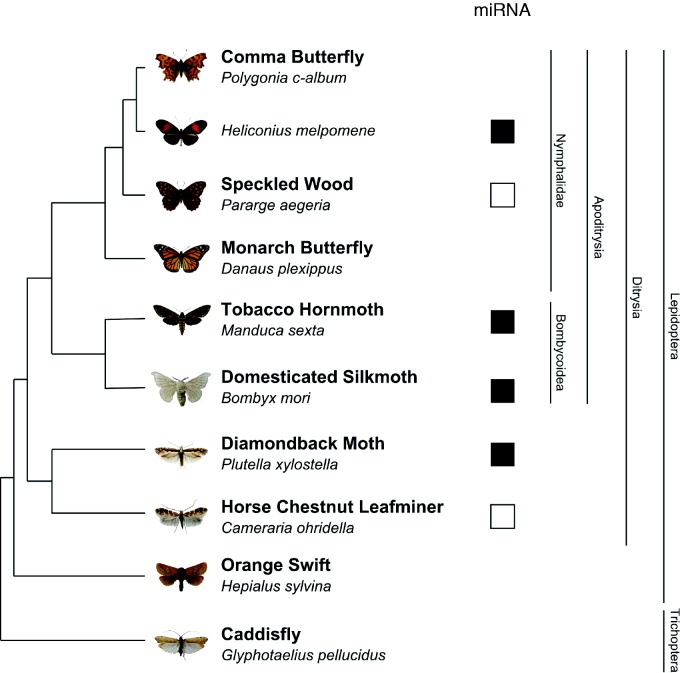
Phylogeny of the lepidopteran species for which genome sequence data is available. The orange swift genome is of insufficient coverage and was not used for assessment of miRNA distribution. Black squares indicate published miRNA sequencing projects and white squares represent the data presented in this manuscript.

Lepidoptera represents an ideal clade to study the relationship between miRNAs and morphological evolution (fig. 1). This group comprises over 150,000 species of moths and butterflies and is one of the most species-rich insect orders. Lepidopterans share a large number of morphological apomorphies and underwent a major evolutionary radiation during the Cretaceous (Regier et al. 2013; Wahlberg et al. 2013; Kawahara and Breinholt 2014) following their divergence from their sister group Trichoptera (caddisflies). The phylogeny of the Lepidoptera is well-studied, with clearly supported superfamilies identified through both morphological and molecular studies (Regier et al. 2013; Wahlberg et al. 2013). In outline, the vast majority of species diversity is found in the Ditrysia, which makes up 99% of extant lepidopteran species (Wahlberg et al. 2013). Within this clade, the Yponomeutoidea (e.g., *Plutella xylostella*) and Gracillarioidea (e.g., *Cameraria ohridella*) are distant sister groups that diverged early from the rest of the Ditrysia (Bazinet et al. 2013; Regier et al. 2013). Most of the remaining ditrysians form a strongly supported clade—the Apoditrysia (Bazinet et al. 2013), which includes the Bombycoidea (including silkmoths and hawkmoths) and the Papilionoidea (butterflies, including the highly diverse nymphalids). One major advantage of using the Lepidoptera for the comparative analysis of miRNA complements is the plethora of genome resources available in the public domain, including sequences from the Domestic Silkmoth *Bombyx mori* (Mita 2004; Xia et al. 2004), the butterflies *Heliconius melpomene* (*Heliconius* Genome Consortium 2012) and *Danaus plexippus* (Zhan et al. 2011), the Diamondback moth *P. xylostella* (You et al. 2013), and the Tobacco Hornworm hawkmoth *Manduca sexta*. Our group has also recently sequenced and assembled draft genomes for the Horse chestnut Leafminer moth *C. ohridella*, the Speckled Wood butterfly *Pararge aegeria*, the Comma butterfly *Polygonia c-album*, and the Orange Swift moth *Hepialus sylvina* (Ferguson et al. 2014). The same study also reports a draft genome assembly for the caddisfly *Glyphotaelius pellucidus* as a representative of the Trichoptera (fig. 1).

However, while genome sequences can be used to search for known miRNA homologues, sampling of actual small RNAs is required for the identification of novel sequences and hence the determination of the miRNA repertoire in a given species. Prior to a recent report (Etebari et al. 2013) on small RNA sequencing in *P. xylostella* (Yponomeutoidea), miRNA data were limited to *B. mori* and *M. sexta* (Bombycoidea) (Liu et al. 2009, 2010) with limited information from wing discs of *H. melpomene* (Surridge et al. 2011). To enlarge the comparative analysis, ensuring the inclusion of distant lineages, the gracillarid *C. ohridella* and the nymphalid butterfly *Pa. aegeria* were selected for small RNA sequencing. Our analyses reveal that a burst of miRNA fixation occurred early in the evolution of the clade. Few miRNA fixations occur on more recent internal branches, even in speciose groups such as the Nymphalidae. We also find high numbers of evolutionarily young, species-specific miRNA sequences, suggesting that high miRNA birth rates are matched by high death rates with few novel genes persisting to integrate stably into developmental programs. We present evidence that one such miRNA has been recruited for a role in segmentation or wing patterning.

## Results

### Small RNA Sampling in C. ohridella and Pa. aegeria

Selection of informative species for miRNA sampling to help elucidate the pattern of miRNA evolution across the Lepidoptera was carried out using three essential criteria: phylogenetic placement, accessibility of different life-cycle stages to afford deep sampling of miRNA species, and availability of genomic sequence of sufficient coverage to enable novel miRNA precursor prediction and detection. The phylogenetic criteria focused our attention on basally branching Lepidoptera lineages and on representation of the speciose clade Nymphalidae, particularly because the published nymphalid miRNA data derive from a restricted tissue type (*H. melpomene* wing disc, Surridge et al. 2011).

Species from basally branching lineages with genomic data include the Orange Swift moth *Hep. sylvina* and the Horse-chestnut leafminer *C. ohridella* (fig. 1). To assess suitability for verification of miRNA sequences, we first used a BLASTn search with 20 conserved miRNAs originating in the common hexapod ancestor or earlier (supplementary file S1, Supplementary Material online). This identified only 12 out of 20 query miRNA sequences in the former genome, but 19 out of 20 in the latter. Furthermore, *C. ohridella* represents a basally divergent lineage within the Ditrysia and a distant sister group to *P. xylostella* (Bazinet et al. 2013; Regier et al. 2013) and provides an excellent basis for comparative analysis of miRNAs originating at the base of the Ditrysian tree. This invasive species is now extraordinarily common in the UK and most life-cycle stages can be readily collected.

The second species chosen for deep sequencing of small RNAs was the Speckled Wood butterfly (*Pa. aegeria*), a nymphalid. This species also has fairly complete genome coverage (19 out of 20 sequences recovered), and life-cycle stages can be collected from a laboratory-reared population.

### Deep Sequencing and miRNA Identification

Small RNA sequencing was undertaken for multiple developmental stages to maximize representation of miRNAs expressed. In *Pa. aegeria* we sampled four time points during embryogenesis (1, 15, 24, and 48 h postoviposition), two larval time points (first and last instar), three pupal time points (0, 3, and 5 days postpupation), and a single adult male and female. *Cameraria ohridella* RNA samples were from larvae, pupae, and adults. In both species, whole bodies, eggs, or pupae were used at each time point for RNA extraction. RNA extracted from individual stages was pooled within each species.

**Fig. 2.**
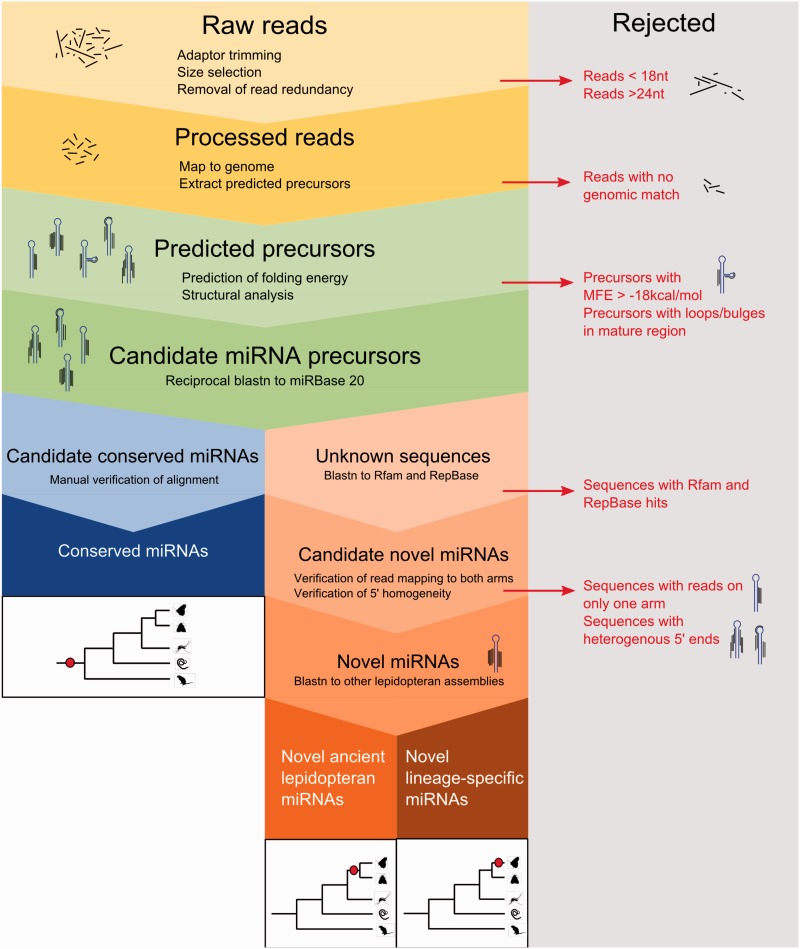
Data processing pipeline for annotation of miRNAs in *Cameraria ohridella* and *Pararge aegeria* from raw Illumina sequencing output.

A total of 80,414,031 raw Illumina HiSeq reads were obtained (table 1) and processed according to the schematic in figure 2, designed to ensure removal of spurious reads, degradation products, and other small RNAs. Because small RNA sequencing yields the mature miRNA products (22 nt), not precursor sequences (∼80 nt), the miRDeep2 algorithm was used to extract predicted miRNA precursor sequences based on the positions of mapped reads in the genome. All predictions generated by miRDeep2 were first subjected to a free energy filtering step to select stable hairpins. Only genomic sequences fulfilling stringent stability criteria were considered to be candidate miRNA precursors.

**Table 1.** Summary of the Small RNA Sequencing Data Sets from *Cameraria ohridella* and *Pararge aegeria*.

	C. ohridella	Pa. aegeria
Stages sampled	Larvae, pupae, adults	Embryos, larvae, pupae, adults
Raw reads	41,621,320	38,792,711
Processed reads	2,338,028	2,813,742
Total predicted miRNAs	113	139
Conserved miRNAs	87 + 3^a^	80 + 2^a^
Lineage-specific miRNAs	23	57

^a^In *C. ohridella*, we identified 87 miRNAs with entries in miRBase 20 as well as three newly reported sequences (Cam-042/Par-110, Cam-110 and Cam-154) shared with other lepidopterans. In *Pa. aegeria*, there were 80 miRNAs with entries in miRBase 20 as well as two newly reported sequences (Cam-042/Par-110 and Par-471). Cam-042 and Par-110 were discovered separately in *C. ohridella* and *Pa. aegeria* and are likely to be homologous miRNAs.

### *Conserved miRNAs in* Cameraria *and* Pararge

Of the candidate precursors identified, we first identified evolutionarily conserved sequences occurring in more than one species. A total of 87 and 80 “conserved miRNAs” of varying evolutionary ages were identified in *C. ohridella* and *Pa. aegeria*, respectively (supplementary file S2, Supplementary Material online). *Cameraria ohridella* has undergone lineage-specific duplications of several miRNA genes including miR-14, miR-2763, and miR-2796 of which only single copies exist in other Lepidoptera.

### *Novel miRNA discovery in* Cameraria *and* Pararge

Following identification of previously documented miRNAs, we filtered the remaining candidates to retain only genuine miRNA genes and classify these into two categories. The first category consists of novel lineage-specific miRNAs found thus far only in the species sampled; the second comprises newly discovered sequences of older origin which may have been missed in previous miRNA sequencing projects.

To ensure removal of non-miRNA gene sequences from our data set, we subjected all remaining sequences to additional validation (fig. 2). In particular, sequences were checked for 5′-homogeneity in the small RNA reads. Sequences with highly heterogenous 5′-ends in their mature reads are unlikely to represent genuine miRNAs as the 5′-end determines the miRNA seed (Kozomara and Griffiths-Jones 2011) and is likely to have effects on target specificity. We discarded these precursors as they could represent degradation products of longer transcripts. As a final conservative criterion, we stipulated that a candidate must have reads mapped to both arms of the hairpin in order to be described as a miRNA.

Comparison to available lepidopteran genome assemblies revealed that our data set includes 23 and 57 lineage-specific miRNAs in *C. ohridella* and *Pa. aegeria*, respectively (supplementary file S2, Supplementary Material online). Only one sequence, “Par471,” was identified by BLASTn in several other ditrysian species but has not been reported in miRBase. The recent publication of a *P. xylostella* miRNA sequencing study (Etebari et al. 2013) has identified a predicted precursor (PC-3p-82883_6) which is likely to be Par-471. Conservation of this sequence was verified by alignment and synteny checks as well as structural analysis of predicted precursors in all species where this sequence is found (supplementary file S3, Supplementary Material online).

### Phylogenetic Placement of Lepidopteran miRNAs

**Fig. 3.**
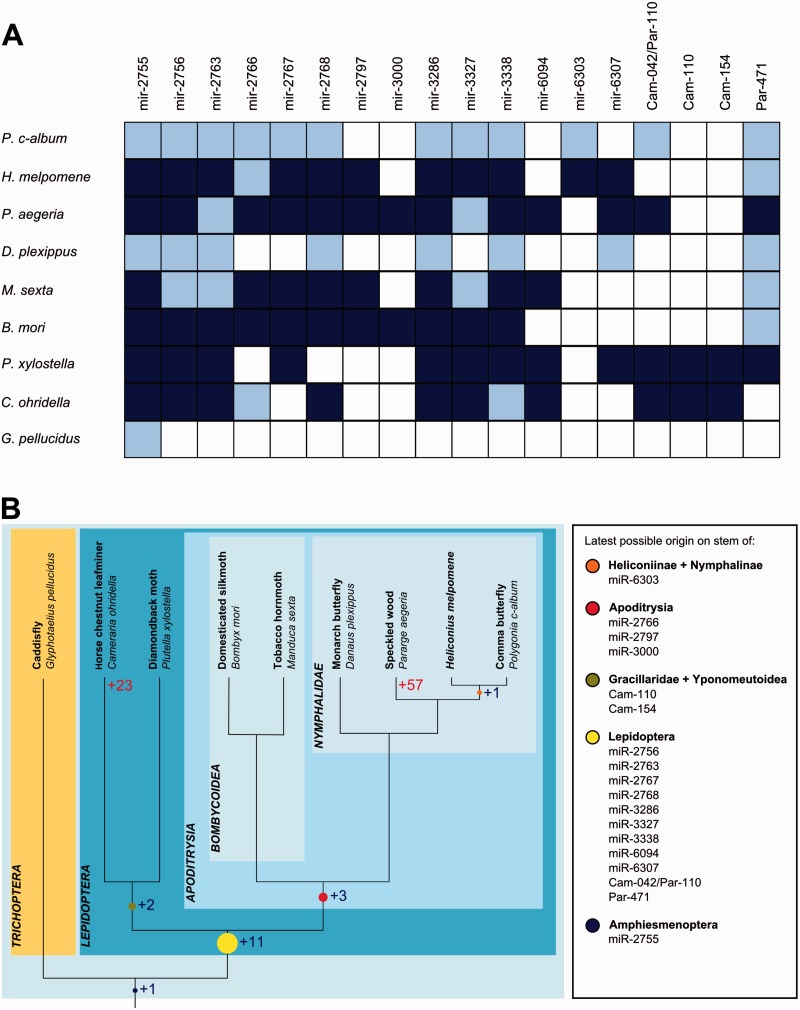
(*A*) Presence/absence matrix for each Lepidopteran-specific miRNA family. Light blue squares indicated BLASTn and structural predictions of presence. Dark blue squares indicate evidence from deep sequencing. (*B*) Phylogenetic distribution of miRNAs in the Lepidoptera and its sister group Trichoptera showing inferred miRNA gains at each node. Lineage-specific gains are only plotted for *Cameraria ohridella* and *Pararge aegeria*.

Using the information revealed by our deep sequencing together with previous studies on lepidopteran miRNAs, we determined the presence or absence of each sequence in each species (fig. 3*A*) and compared these to a phylogeny of the Lepidoptera (Regier et al. 2013). This revealed the latest possible node of origin for each sequence (fig. 3*B*). The caddisfly (*G. pellucidus*, Trichoptera) was used as an outgroup to the Lepidoptera. We validated the completeness of this genome by running BLASTn using the same set of 20 conserved miRNAs described previously (supplementary file S1, Supplementary Material online). Nineteen out of 20 of the query sequences were retrieved, suggesting that genome coverage of *G. pellucidus* is adequate and that the likelihood of miRNA genes reported absent as an artefact of genome completeness is low.

As expected, many miRNA precursors had origins prior to the last common ancestor of Lepidoptera and Trichoptera; these are not the focus of this study. A total of 459 sequences were identified which were unique to the Lepidoptera and/or Trichoptera. The majority of these are lineage-specific: 315 from *B. mori*, 10 from *H. melpomene*, 8 from *M. sexta*, 23 from *C. ohridella*, and 57 from *Pa. aegeria*. Of the novel sequences reported in *P. xylostella* (Etebari et al. 2013), we identified 21 sequences with reads mapping to both arms which are likely to be miRNA precursors (supplementary file S4, Supplementary Material online).

A total of 25 potential miRNA precursors were found to be present in Lepidoptera and Trichoptera, and shared by at least two species. Six of these (identified in previous studies) were removed from further analysis as we are not confident that they represent genuine miRNAs under our stringent criteria. These are miR-2739, miR-3389, miR-2733, miR-6495, miR-6496, and miR-6497. The miR-2738 sequence has a predicted secondary structure with an asymmetric bulge in its mature region; its pattern of read mapping in *B. mori* is suggestive of a degradation product. miR-3389 displays a heterogenous pattern of read mapping on its 5′-arm, again suggestive of a degradation product, and no reads from its 3′-arm have been reported. miR-2733 maps to several different genomic regions in *B. mori* and may be derived from a repetitive element. The miR-6495, miR-6496, and miR-6497 sequences were found to be derived from ribosomal RNA fragments.

We searched for the remaining 19 sequences in all available lepidopteran and trichopteran genome assemblies using BLASTn. The miR-2779 sequence was removed from further analysis as we are unable to confirm that the predicted precursors for this sequence identified in *B. mori*, *M. sexta*, and *P. xylostella* are homologous to each other by structure or synteny.

We plotted the latest possible origin for each miRNA onto the lepidopteran phylogeny (fig. 3*B*). Only one sequence (miR-2755) is shared between the Lepidoptera and its sister group Trichoptera. Strikingly, we find that the majority of novel miRNA acquisition is concentrated at the base of the lepidopteran tree, with 11 of these novel sequences likely to have been present in the last common lepidopteran ancestor. Two other sequences (Cam-110 and Cam-154) unite the distantly related sister species *C. ohridella* and *P. xylostella*. There were three further innovations on the Apoditrysian stem. We find very little miRNA acquisition on more recent internodes, with a single gain (miR-6303) in a common ancestor of the Heliconiinae and Nymphalinae. The current miRNA distribution also suggests that several lineage-specific losses may have occurred, though we have less certainty about miRNA loss due to the limitations of genome assemblies.

### cubitus interruptus *Is a Potential Target for miR-2768*

**Fig. 4.**
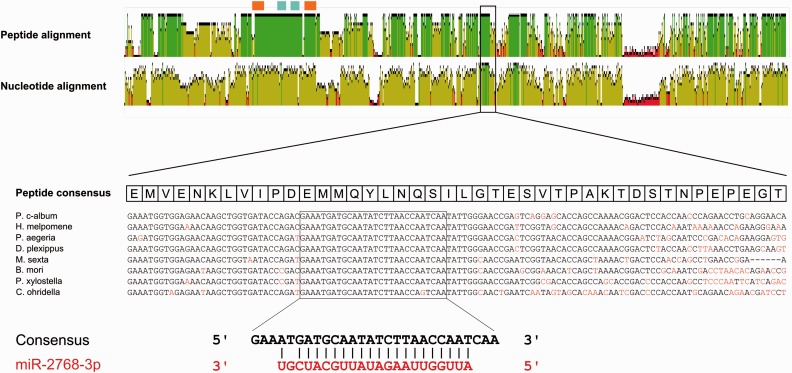
Conservation of the peptide (top graph) and nucleotide (lower graph) sequence across the length of the *ci* gene in eight lepidopteran species. Positions with 100% mean pairwise identity between all pairs in the column are colored in green. Brown indicates positions with identity under 100% but above 30%. Red indicates less than 30% identity. The orange boxes show the positions of zinc finger C2H2 domains and blue boxes indicate the position of the zinc finger double domains. A closeup is shown of the nucleotide sequence alignment in the region of the putative miR-2768 binding site. Residues differing from the consensus are colored in red.

Given the acquisition of new miRNAs early in lepidopteran evolution, we sought to identify target genes. This analysis is hampered by the lack of well-annotated lepidopteran transcriptomes with documented 3′-UTRs, the target of many animal miRNAs. However, applying the PITA target prediction algorithm (Kertesz et al. 2007) to annotated coding sequences from the *H. melpomene* transcriptome (Hines et al. 2012) revealed an almost perfectly paired match between miR-2768 and the *cubitus interruptus* (*ci*) transcript. Subsequent analyses of the corresponding region in other lepidopterans reveal that this site is highly conserved between butterflies and moths (fig. 4), with 21 of the 22 bases (including the seed sequence) in miR-2768-3p capable of pairing with the consensus candidate site. This complementarity is greater than that observed between most bilaterian miRNAs and their targets. The miR-2768-3p sequence itself is also completely conserved in all lepidopteran species studied (supplementary file S5, Supplementary Material online) and its precursor gene lies in an intron of the *invected* (*inv*) gene.

To gauge if the candidate site in *ci* is identical between species by chance, or as a by-product of conservation of amino acid sequence, we assessed the overall conservation of the *ci* gene across Lepidoptera. Using genome data for three lepidopterans (*B. mori*, *D. plexippus*, and *H. melpomene*), we aligned predicted *ci* coding sequences and compared nucleotide and deduced peptide sequences (fig. 4). This revealed that while the peptide sequence of Ci is divergent between different insect orders (supplementary file S5, Supplementary Material online) and only aligns well in the region containing the zinc finger domains, in Lepidoptera it is more conserved across much of its length (fig. 4, supplementary file S5, Supplementary Material online). Consistent with selection acting at the amino acid level, the corresponding nucleotide sequence is weakly conserved with the majority of the coding sequence showing evidence of third codon position wobble. However, there are two clear regions in the predicted *ci* cDNAs where the nucleotide sequence is almost completely conserved, and strikingly one of these contains the putative miR-2768-3p target site. This implies there is a reason other than amino acid constraints that has forced conservation of local sequence, consistent with conservation as a target site for interaction with miR-2768-3p.

**Fig. 5.**
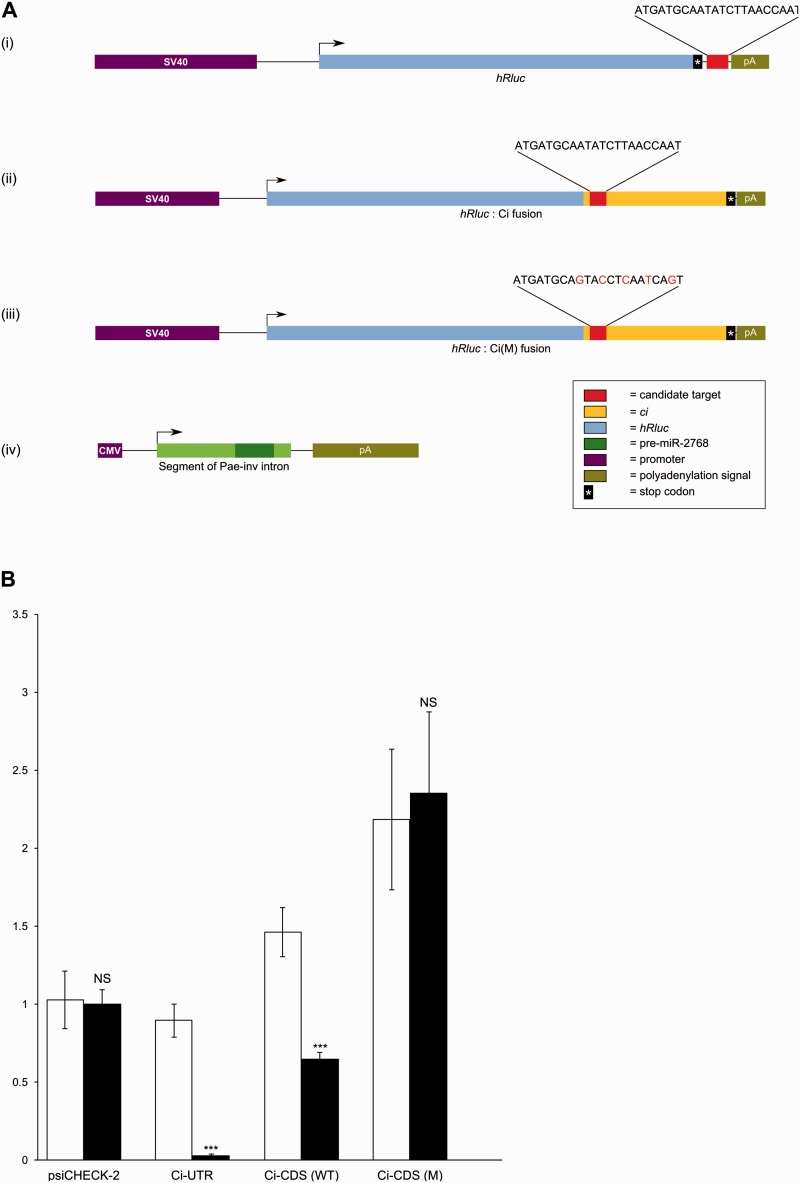
(*A*) Constructs used for functional validation of Pae-miR-2768 binding to its predicted site in *Pae-ci*. (i) psiCHECK-2:Ci-UTR, (ii) psiCHECK-2:Ci-CDS(WT) (iii) psiCHECK-2:Ci-CDS (M), (iv) pCS2+:2768. (*B*) Pooled luciferase assay data for the candidate miR-2768 target site in the presence or absence of miR-2768, and for wild-type and mutant *hRluc*:*ci* fusion constructs in the presence or absence of miR-2768. Error bars represent standard deviation. ***P < 0.001; NS, not significant.

In order to functionally test the interaction of miR-2768-3 p with its predicted target, we cloned the consensus 22 bp candidate site from lepidopteran *ci* into a luciferase expression vector downstream of the *hRluc* Renilla luciferase stop codon (fig. 5*A* (i); construct psiCHECK-2:Ci-UTR). The vector also contains a synthetic firefly luciferase gene (*hluc+*) as a control for transfection efficiency. We also generated an expression construct for miR-2768 by cloning a 339 bp fragment of the *inv* intron containing the miR-2768 precursor sequence under a constitutive promoter (fig. 5*A* (iv); construct pCS2+:2768). Following cotransfection, the ratio of Renilla:firefly luciferase expression is used as a measure of miRNA activity (fig. 5*B*). We detect a very strong (∼37-fold) downregulation of Renilla luciferase expression in the presence of both miR-2768 and its candidate site from *ci*. In the absence of the miRNA, no downregulation is observed. This confirms that the miR-2768 precursor region is capable of being processed into a functional miRNA in cells, and that this downregulates transcripts containing the predicted binding site, such as lepidopteran *ci* gene.

As the identified candidate site lies within the coding sequence, we further tested the ability of miR-2768 to downregulate a target when the site occurs within a coding region. To do this, we modified the *hRluc* gene on the psiCHECK-2 vector to encode a fusion protein between *hRluc* and the *ci* exon containing the target site (fig. 5*A* (ii); construct psiCHECK-2:Ci-CDS). A mutant version of this construct was also generated with five synonymous transition mutations introduced into the candidate target site (fig. 5*A* (iii); construct Ci-CDS(M), supplementary file S5, Supplementary Material online). These mutations should ensure that the protein sequence is not affected, while simultaneously disrupting miRNA binding. Both constructs were assayed for Renilla:firefly luciferase activity in the presence and absence of miR-2768. We detect downregulation of expression from the wild-type construct in the presence of miR-2768, but there is no downregulation of the mutant fusion construct (fig. 5*B*). A slight increase in Renilla:firefly luciferase activity was observed in the mutant construct relative to the wild-type irrespective of the presence of miR-2768. Overall, these experiments support our hypothesis that miR-2768 is capable of binding to the target sequence from the *ci* gene and exerting downregulation within coding sequences.

## Discussion

### *miRNAs Discovery in* C. ohridella *and* Pa. aegeria

Using small RNA sequencing, we identified 113 and 139 miRNA genes in the Horse chestnut Leafminer moth *C. ohridella* and the Speckled Wood butterfly *Pa. aegeria*, respectively. It is likely that these are not fully comprehensive figures, because some conserved ancient miRNAs known from other animals were not detected, perhaps due to highly restricted spatiotemporal expression. Although the number of sequences shared with at least one other species is similar in both organisms (90 in *C. ohridella*; 82 in *Pa. aegeria*), the number of lineage-specific sequences identified in *Pa. aegeria* (57) is more than detected in *C. ohridella* (23) despite equal sequencing depth in both species. We suggest this results from the difference in the range of developmental stages sampled in each species. *Pararge aegeria* was extensively sampled at different time points for each developmental stage, including embryos; in contrast, we lack data on small RNAs expressed during embryonic development in *C. ohridella*. Consistent with this, research in drosophilid fruitflies has shown that many young, fast-evolving miRNAs are highly expressed during embryogenesis and are downregulated during later development (Ninova et al. 2014). It is possible that the early embryo provides a more permissive environment for gene expression changes; this suggestion is compatible with “phylogenetic hourglass” models that identify early developmental stages as prone to evolutionary change by incorporation of newly evolved genes (Domazet-Lošo and Tautz 2010).

Most of the evolutionarily “older” miRNAs have been reported before. This is not surprising because conserved miRNAs have been the focus of much study. We did identify one additional conserved miRNA, Par-471, from *Pa. aegeria* which we suggest is widely distributed across Lepidoptera, because its precursor sequence could be identified in most lepidopteran genome sequences. This miRNA is also present in a small RNA data set from the Diamondback moth *P. xylostella* (Etebari et al. 2013). Although the precursor has been conserved in sequence and structure across other lepidopterans (supplementary file S5, Supplementary Material online), it has not been reported in deep sequencing projects in the Apoditrysia. The mature Par-471 miRNA is not highly represented in our pooled *Pa. aegeria* library suggesting it may have a spatiotemporally restricted expression pattern.

### Birth and Death of miRNAs in the Lepidoptera

Assigning miRNA genes to nodes on a phylogenetic tree of Lepidoptera yielded two seemingly paradoxical conclusions. First, there is extensive birth of new miRNAs along terminal branches in the phylogeny generating a plethora of young miRNAs. Second, most of the “conserved” miRNAs, shared by more than one species of Lepidoptera, originated early in phylogeny, with little evidence for integration of new miRNAs in the 100 My (Wahlberg et al. 2013) following the initial radiation of the Lepidoptera. How can these two conclusions be reconciled?

The present-day miRNA complement of any given lineage is the net result of the opposing forces of miRNA birth and miRNA death. The birth of new miRNAs may occur through de novo generation of new precursors from existing hairpin-like segments of existing transcripts (Campo-Paysaa et al. 2011) or through the duplication and divergence of existing miRNA genes. miRNAs may also be lost through mutations that disrupt the formation of a stable stem loop structure. The balance between these forces in shaping miRNA evolution throughout the Metazoa has been a subject of debate. Some authors have argued for a continuous addition of miRNAs to metazoan genomes over time with very low rates of secondary loss (Tarver et al 2013); others have presented evidence for a more fluid model with continuous gains and losses operating on each stem in a clade (Meunier et al. 2013). What is clear, and also supported by our data, is that terminal nodes in a phylogeny tend to have an abundance of young, lineage-specific miRNAs (Lu et al. 2008; Nozawa et al. 2010; Meunier et al. 2013; Lyu et al. 2014). This was reported by Lu et al. (2008) in drosophilids (Lu et al. 2008) and although this study was subsequently criticized for overestimating lineage-specific miRNA gains through sampling of possible degradation products (Berezikov et al. 2010), evidence for a high miRNA birth rate on terminal branches was also later presented for mammals (Meunier et al. 2013) and confirmed in drosophilids (Nozawa et al. 2010; Lyu et al. 2014). In Lepidoptera, the number of lineage-specific miRNAs identified with available miRNA deep-sequencing data are 23 in *C. ohridella*, 21 in *P. xylostella*, 315 in *B. mori*, 8 in *M. sexta*, 57 in *Pa. aegeria*, and 10 in *H. melpomene*. Although the exact numbers quoted for each lineage vary (probably due to depth of transcriptome sequencing and rigor of miRNA annotation), this is still indicative of the continuous birth of new miRNA sequences on terminal branches within the lepidopteran phylogeny.

It is estimated that a large proportion of new miRNAs (>94% in drosophilids) will rapidly be lost (Lyu et al. 2014). The quick turnover phase in miRNA evolution is estimated to last for about 30 My in drosophilids, after which the remaining sequences will have been integrated into gene regulatory pathways. It is widely thought that miRNAs incorporated into such pathways are then unlikely to be lost, although there are exceptions. For example, secondary loss of conserved miRNAs have been noted in tapeworms, which may be linked to morphological simplification (Fromm et al. 2013), and loss of conserved miRNAs has also been documented in mammals (Meunier et al. 2013).

It is likely that the constant generation of new miRNA precursors has been going on throughout lepidopteran evolution. Birth rates of novel miRNAs are unlikely to drop substantially given the low requirements for transcripts to adopt a suitable secondary structure and become integrated into the miRNA processing pathway (Campo-Paysaa et al. 2011; Lyu et al. 2014), though it has been postulated that they may sometimes be increased through increased transcriptional activity in the genome (Heimberg et al. 2008). Despite this constant production of new material for selection to act upon, we find that hardly any new miRNAs have been integrated into lepidopteran miRNA repertoires following the establishment of the deeper branches in lepidopteran evolution. The distribution of lepidopteran-specific miRNAs shows the majority of innovation occurring along the Lepidopteran stem following divergence from the Trichoptera. Only a single sequence discovered so far, miR-2755, was acquired along the common lineage leading to the Lepidoptera and Trichoptera, and few or no conserved sequences were acquired in nodes such as Nymphalidae or Bombycoidea. Hence, we suggest that the concentration of new miRNA sequences close to the base of Lepidoptera, around 100 Ma (Wahlberg et al. 2013), was not a burst of accelerated miRNA birth, but rather a decrease in miRNA death rate. We argue that early in lepidopteran evolution, a set of new miRNA genes were incorporated into genetic regulatory pathways. Such pathways might include those governing lepidopteran-specific characters such as the patterning of wing and body scales, color vision, or interaction with angiosperms.

### The ci Gene Is a Likely Target for miR-2768

To understand how novel lepidopteran miRNAs were incorporated into genetic regulatory pathways, it is necessary to identify candidate target genes which these miRNAs regulate. The majority of identified target sites for metazoan miRNAs lie within 3′-UTRs, possibly due to functional constraints (Grimson et al. 2007; Gu et al. 2009). This phenomenon may be somewhat exaggerated, however, as many miRNA target prediction programs consider only 3′-UTRs in predictions of miRNA binding (Lewis et al. 2005; Wang and El Naqa 2008; Maragkakis et al. 2009). Indeed, miRNA target sites located within coding sequences have been documented in animals (Forman et al. 2008; Tay et al. 2008), including humans (Forman et al. 2008), and have recently been suggested to be a common occurrence in cnidarians (Moran et al. 2014). There is some evidence suggesting that miRNA binding in coding regions may require higher sequence complementarity between the miRNA and its target, and proceed via RISC-mediated cleavage (Duursma et al. 2008).

We found evidence that the transcript from the *ci* gene is a target for downregulation by one of the novel lepidopteran miRNAs, miR-2768, within its coding sequence. All lepidopteran *ci* cDNAs contain a sequence which is an almost perfect complement to miR-2768-3p. This candidate target is strikingly well-conserved at the nucleotide level in all species analyzed, even though the majority of the *ci* coding sequence is not, suggestive of selection for function on its nucleotide sequence rather than only on its peptide sequence. Luciferase assays conducted in cell culture also indicate that this target site is capable of being targeted and downregulated by miR-2768, both when located within the 3′-UTR as well as when present as part of the coding sequence. We suggest this miRNA-target interaction arose early in lepidopteran evolution, since we find that the miR-2768 precursor sequence is present in all lepidopteran genomes examined, but absent in the caddisfly *G. pellucidus*. We also discovered that the miR-2768 gene itself is located in an intron of the homeobox gene *inv*, in the same transcriptional orientation, suggesting a mechanism by which miR-2768 was recruited to regulate *ci*.

**Fig. 6.**
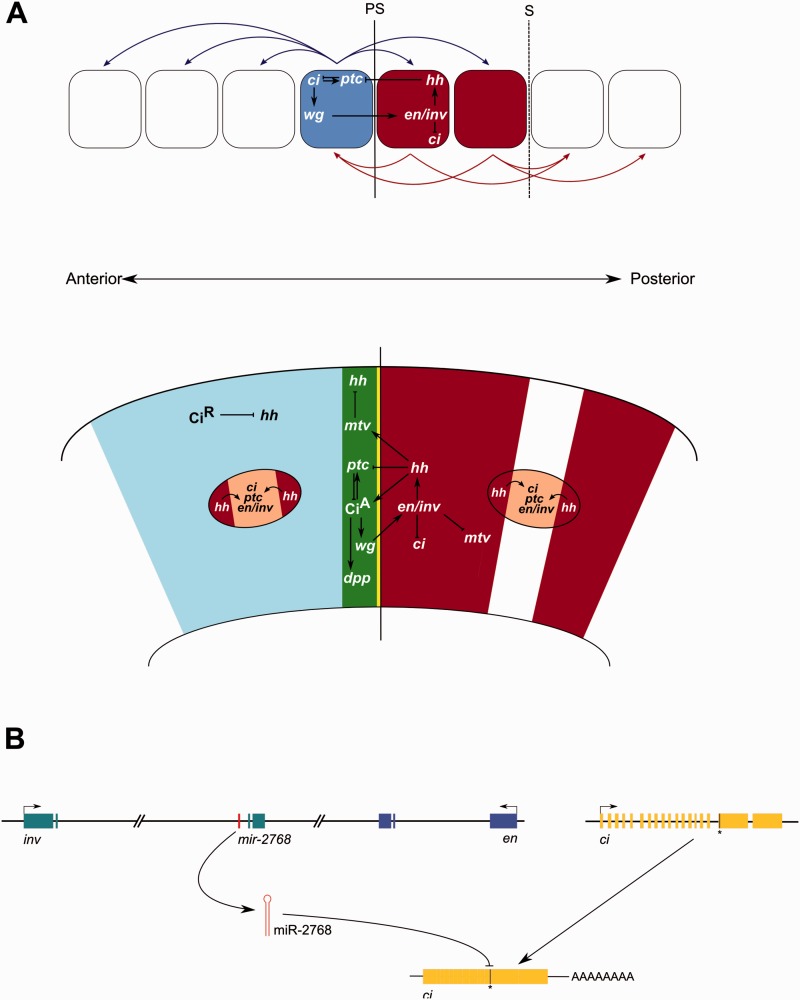
(*A*) Involvement of *ci* in the Hedgehog signaling pathway during anteroposterior patterning of the *Drosophila melanogaster* embryo (above) and wing imaginal disc patterning in *Precis coenia* (below). PS, parasegment boundary. S, segment boundary. (*B*) Model for the contribution of miR-2768 to the repression of *ci*. The asterisk indicates the position of the miR-2768-3p binding site in the coding region of the *ci* messenger RNA.

We postulate that miR-2768 serves to downregulate the expression of *ci* during butterfly and moth morphogenesis, possibly during segmentation of the embryo or during postembryonic patterning of the wing primordia, or indeed both processes. Ci is the insect homologue of the vertebrate Gli protein family and functions as an effector of Hedgehog (Hh) signaling. The Hh signaling pathway is essential in the establishment of compartment boundaries during anteroposterior segmentation of the insect embryo (Ingham and McMahon 2001), subsequent anteroposterior patterning of the cuticle in *Drosophila* (Sanson et al. 1999), as well as in the wing imaginal disc during late larval and pupal stages in insects (Keys 1999; Ingham and McMahon 2001). Common to these systems is a regulatory circuit (fig. 6) whereby the Hh ligand, expressed in posterior compartment cells under the influence of Engrailed (En) and its paralogue Invected (Inv), signals anteriorly across the compartment boundary to activate *wg* expression. The transcription factor acting downstream of the Hh signal to induce expression of *wg* and other downstream genes is Ci. Wg signals back across the compartment boundary to maintain expression of *en/inv*. In the *en/inv* domain, *ci* is not expressed due to repression by En/Inv. Regulation of Ci activity is essential as it mediates many of the downstream actions of Hh. This is achieved both at the transcriptional level through the actions of En/Inv in repressing *ci* transcription, as well as through posttranslational modifications to the Ci protein which regulate its localization and activity. We propose that in the Lepidoptera, miR-2768 functions to add another level of regulation over the Ci activity at a posttranscriptional level. As miR-2768 is located within the transcription unit of *inv*, it is likely to be coexpressed with *inv* (Rodriguez et al. 2004; Baskerville and Bartel 2005) and may reinforce the repression of *en/inv* on *ci*.

There is another system where Hh signaling has been co-opted for use in patterning where the gene interactions described above have been modified. Many butterflies and moths have large, brightly colored eyespots on their wings. Eyespot morphology and genes involved in determining eyespot color and pattern may differ between species (Brunetti et al. 2001; Oliver et al. 2012), and the regulatory networks behind eyespot focal specification have only been studied in detail only in Nymphalidae (Oliver et al. 2012). In their report, they propose that a network co-option event occurred in the evolution of eyespot focal patterning and was subsequently modified in different lineages to generate novel patterns. In *Bicyclus anynana* and *Junonia coenia*, focal development first involves an accumulation of *hh* in cells flanking developing foci. Hh signaling subsequently induces expression of *ptc*, *ci*, and *en/inv* in cells which will form the focus (Keys 1999; fig. 6). This process of eyespot specification occurs regardless of whether the focus develops in the anterior of posterior compartment, implying a relaxation of compartmental restrictions. Also relaxed must be the transcriptional repression of *ci* by *en/inv* as both are coexpressed in the same cells. It has been noted that some miRNAs are coexpressed with their target genes, and it has been postulated that their role may be to subtly modulate target mRNA levels. We speculate that perhaps miR-2768, produced from the intron of *inv*, may subtly regulate the levels of *ci* mRNA in regions of coexpression such as eyespots.

### Concluding Remarks

We demonstrate that the majority of miRNA innovation retained by selection within the Lepidoptera arose early in lepidopteran evolution and suggest that novel lepidopteran miRNAs were recruited for regulatory roles in embryonic and wing patterning. We describe a novel gene regulatory network involving *ci*, *inv*, and miR-2768 in butterflies and moths, in which miR-2768, generated from an intron of *inv*, downregulates *ci*. This network may be involved in segmentation and in patterning of wing primordia in Lepidoptera.

## Materials and Methods

### Sample Collection

*Cameraria ohridella* larvae and pupae were obtained by dissection from leaf mines on Horse chestnut (*Aesculus hippocastanum*) leaves collected in Oxford University Parks, UK, in July 2013. Adults were collected by incubating leaves in a damp environment in the laboratory at room temperature. Twenty individuals were sampled for each developmental stage. Adults were not sexed.

*Pararge aegeria* specimens were obtained from a colony at Oxford Brookes University originally founded with specimens from Belgium (Carter et al. 2013). Eggs were collected within 20 min of oviposition and homogenized 1, 15, 24, and 48 h after oviposition. For first instar larvae, a mixture of whole larvae was homogenized for a total mass of 0.10 g. Two final instar larvae (total mass = 0.11 g) were used. Single whole pupae were sampled at 0, 3, and 5 days postpupation. One adult male and one adult female were homogenized separately following removal of the wings and legs.

### RNA Extraction and miRNA Sequencing

Small RNA extraction was carried out using the miRVana miRNA Isolation Kit (Life Technologies, AM1560) following the manufacturer’s protocol. Tissues were homogenized in miRVana Lysis Buffer using a single bead in the TissueLyser II (Qiagen). All tissues were homogenized fresh.

Quantity and quality of the extracted RNA was assessed using the Experion Automated Electrophoresis System (Bio-Rad) on an Experion HighSens chip. Sample pooling was carried out for each species such that equal amounts of RNA were used from each sampling time point. Library preparation and deep sequencing were carried out at the Beijing Genomics Institute (Hong Kong). The two libraries were barcoded and run on a single lane of the Illumina HiSeq 2000 platform. Raw read and processed data are available on the Gene Expression Omnibus (accession number GSE63644).

### Computational Analysis

We developed a conservative pipeline for identification of true miRNA sequences in the small RNA sequencing reads (fig. 2). FastQC (http://www.bioinformatics.babraham.ac.uk/projects/fastqc, last accessed January 23, 2015) was first used to validate read quality. Raw reads were processed by converting FASTQ to FASTA file formats and removing adapter sequences. Reads were collapsed to remove redundancy; collapsed reads less than 18 nt and greater than 24 nt were discarded. Remaining reads were mapped to Bowtie-indexed genome assemblies and those that failed to align were discarded.

The miRDeep2 algorithm (Friedländer et al. 2012) was used to predict precursor hairpins at genomic locations where the reads mapped. Each predicted precursor was then manually inspected for the following criteria before being accepted as a true putative miRNA: stable hairpin structure with less than −18 kcal/mol free energy, at least 18 paired bases on the main stem, and the absence of large internal loops and bulges in mature regions. Free energy of the predicted precursors was calculated using RNAeval.

For conserved miRNA identification, all precursors passing the structural criteria were checked against known precursors in miRBase 20 using the following BLASTn parameters: word_size = 4, reward = 5, penalty -4, gapopen = 8, and gapextend = 6. BLAST hits were assessed and verified by reciprocal BLASTn. Verified sequences were annotated as conserved miRNAs. Sequences without BLASTn hits to miRBase 20 were subjected to further processing and retained only if they passed the following criteria: homogeneity of 5′-ends in mature products, read mapping to both arms of the hairpin, and no BLASTn hits to RepBase, Rfam, or NCBI bacterial sequences (taxid:2). The positions where each candidate precursor mapped onto the genome were screened to remove duplicate, overlapping predictions.

### Phylogenetic Placement of Lepidopteran miRNAs

All documented miRNA precursor sequences were downloaded from miRBase 20 (Kozomara and Griffiths-Jones 2011) and filtered to remove sequences present outside of the Lepidoptera (represented by *B. mori*, *M. sexta*, and *H. melpomene*). Lepidopteran-specific miRBase entries, along with sequences reported here, were then compared by BLASTn against genome assemblies for *H. melpomene* (www.butterflygenome.org, last accessed January 23, 2015), *D. plexippus* (http://monarchbase.umassmed.edu/, last accessed January 23, 2015), *M. sexta* (https://www.hgsc.bcm.edu/arthropods/tobacco-hornworm-genome-project, last accessed January 23, 2015), *B. mori* (http://sgp.dna.affrc.go.jp/KAIKObase/ and http://silkworm.genomics.org.cn/silkdb/, last accessed January 23, 2015), *P. xylostella* (http://dbm.dna.affrc.go.jp/px/, last accessed January 23, 2015), *G. pellucidus*, *Hep. sylvina*, *C. ohridella*, *Pa. aegeria*, and *Polygonia c-album* using the BLASTn parameters described above. Genome assembly data for *G. pellucidus*, *Hep. sylvina*, *C. ohridella*, and *Pa. aegeria* are also publicly available in the Oxford University Research Data archive (doi: 10.5287/bodleiandury.3) and raw sequence data are available in the NCBI BioProject database (accession number PRJNA241175), as reported in Ferguson et al. (2014). Each species was scored for presence or absence of individual miRNA families. Where possible, syntenic conservation was verified by comparing the positions of the miRNA and its flanking genes between species.

### miRNA Target Predictions

Transcriptome data for *H. melpomene* were downloaded from EnsemblMetazoa. The PITA target prediction algorithm (Kertesz et al. 2007) with default parameters was run on all contigs using a cutoff score of −20. This algorithm computes the difference in free energy gained upon pairing of the miRNA to its target and free energy lost in opening up RNA secondary structure in order to make the site accessible to miRNA binding.

### Vector Construction

A synthetic oligonucleotide duplex was constructed consisting of the predicted miR-2768 target site (22 bp) from the *Pa. aegeria ci* gene flanked by restriction sites and inserted into the multiple cloning site of the psiCHECK-2 vector (Promega, C8021) downstream of the *hRluc* stop codon to make the psiCHECK-2:Ci-UTR construct. To generate a construct, psiCHECK-2:Ci-CDS(WT), with the putative *ci* target site within its coding region, a 369 bp region of the *ci* exon containing the site was cloned and inserted immediately preceding the stop codon of *hRluc*. A mutated version with synonymous transition mutations within the candidate site was generated by polymerase chain reaction (PCR) to give the psiCHECK-2:Ci-CDS(M) construct. All constructs were validated by sequencing. For construction of the miR-2768 expression vector, a 339 bp segment containing the miRNA precursor was amplified by PCR from *Pa. aegeria* genomic DNA and inserted into the multiple cloning site of pCS2+.

### Luciferase Assay Protocol

HeLa cells (ATCC CCL-2) were cultured in 25-cm^2^ flasks at 37 °C, 5% CO_2_ in Dulbecco’s modified Eagle’s medium (Sigma-Aldrich, D5671) supplemented with 10% fetal calf serum, 2 mM l-glutamine, and penicillin/streptomycin. Cells were passaged and transferred to 96-well assay plates (Sigma-Aldrich, CLS3603) 36 h prior to transfection. The time between plating and transfection was optimized experimentally for maximal transfection efficiency. Transfection was carried out using FuGENE HD (Promega, E2311). Each transfection reaction was set up according to the manufacturer’s protocol in 100 µl using a total of 2 µg of plasmid DNA and 6 µl of FuGENE HD. Luciferase assays were performed 36 h after transfection with the Dual-Glo Luciferase Assay System (Promega, E2920). Firefly and Renilla luciferase expression was quantified by measuring luminescence with no emission filter on the FLUOstar Omega microplate reader (BMG LABTECH). For each assay, three biological replicates (two for psiCHECK-2:Ci-UTR without miR-2768) on separate plates were performed at separate times and relative Renilla and firefly luciferase activities were recorded. Each biological replicate consisted of three or five technical replicates. The mean Renilla:firefly ratio for pCS2+:2768 cotransfected with blank psiCHECK-2 was used to normalize readings across plates.

## Supplementary Material

Supplementary files S1–S5 are available at *Molecular Biology and Evolution* online (http://www.mbe.oxfordjournals.org/).

Supplementary Data
