# Supplementary material for: A Burst of miRNA Innovation in the Early Evolution of Butterflies and Moths
Source: Mol Biol Evol. 2015 Jan 8;32(5):1161–74. doi: 10.1093/molbev/msv004 (PMC4408404; doi:10.1093/molbev/msv004)
Supplement: Supplementary Data [file supp_32_5_1161__index.html]

A burst of miRNA innovation in the early evolution of butterflies and moths — A Burst of miRNA Innovation in the Early Evolution of Butterflies and Moths — A Burst of miRNA Innovation in the Early Evolution of Butterflies and Moths — Supplementary Data 

# A Burst of miRNA Innovation in the Early Evolution of Butterflies and Moths

## Supplementary Data

files

**Files in this Data Supplement:**

- Supplementary Data - pdf file
- Supplementary Data - pdf file
- Supplementary Data - pdf file
- Supplementary Data - pdf file
- Supplementary Data - pdf file
