# Supplementary material for: A Burst of miRNA Innovation in the Early Evolution of Butterflies and Moths
Source: Mol Biol Evol. 2015 Jan 8;32(5):1161–74. doi: 10.1093/molbev/msv004 (PMC4408404; doi:10.1093/molbev/msv004)
Supplement: Supplementary Data [file supp_msv004_Supplement_S1.pdf]

Supplement S1: List of miRNAs of ancient origin used to verify genome assemblies and miRNA data

			Recovered in						
					Cameraria ohridella		*Pararge aegeria*		
**miRNA name**	**Precursor sequence**	**Origin**	***Glyphotaelius pellucidus***	***Hepialus sylvina***	**Genome**	**miRNA sequencing**	**Genome**	**miRNA sequencing**	***Polygonia c-album***
bmo-let-7	GGUACUGCCGUCGGCUUGUUGAGGUAGUAGGUUGUAUAGUACGGAAAUACAACACAUAGGUGCGACUGUAUAGCCUGCUAACUUUCCGAGCUGACGGAAUGACA	Bilateria	Yes	Yes	Yes	Yes	Yes	Yes	Yes
bmo-bantam	UAAAAGGAACUACGAAACUGGUUUUCAUAAUGAUUUGACAGAUUGUUUUGUAUUCUGAGAUCAUUGUGAAAGCUAAUUUUGUUCCUGGUA	Protostomia	Yes	Yes	Yes	Yes	Yes	Yes	Yes
bmo-mir-iab-4	GCGUCGCCUUCUGUACGUAUACUGAAUGUAUCCUGAGUGCGCGAUUCUUGUCCGGUAUACCUUCAGUAUACGUAACAGAAGAUGACAC	Arthropoda	Yes	Yes	Yes	Yes	Yes	Yes	Yes
bmo-mir-1a	AGCCUUGCGCAAGUUCCGUGCUUCCUUACUUCCCAUAGUCAUUGUAAUCAUAUGGAAUGUAAAGAAGUAUGGAGCUGCGCGGGCG	Bilateria	No	Yes	Yes	Yes	Yes	Yes	Yes
bmo-mir-2a-1	GGCGGAAUGUAGUCGUGGCAUCAAAGUCGGUUUGUCAUAGGUCAUAACGUAACUAUCACAGCCAGCUUUGAUGAGCACGACUUCAUCUGU	Protostomia	Yes	Yes	Yes	Yes	Yes	Yes	Yes
bmo-mir-7	CGCUUCGUGUUGUAUGGAAGACUAGUGAUUUUGUUGUUUUUGUUGACUAACAAGAAAUCACUAAUCUGCCUACAAAGCGACAGCA	Bilateria	Yes	No	Yes	Yes	Yes	Yes	Yes
bmo-mir-8	CACGACGGAGUAACGGUUCGCAUCUUACCGGGCAGCAUUAGAGUCCUGUCUAUAUUUUCUAAUACUGUCAGGUAAAGAUGUCGUCCGCGCUCCACGUUCGUC	Bilateria	Yes	Yes	Yes	Yes	No	Yes	Yes
bmo-mir-9a	AGUAGAUUGGUUAAUUAUCUUUGGUUAUCUAGCUGUAUGAGUAUUACUGACGUCAUAAAGCUAGGUUACCGGAGUUAAGUGCCGUCUACA	Bilateria	Yes	Yes	Yes	Yes	Yes	Yes	Yes
bmo-mir-10	AGUGCCCUACAUCUACCCUGUAGAUCCGAAUUUGUUUGAAGUGAGGCGACAAAUUCGGUUCUAGAGAGGUUUGUGUGGUGCACG	Eumetazoa	Yes	Yes	Yes	Yes	Yes	Yes	Yes
bmo-mir-11	GUUUUGUGGCUAGCACUCGACAUGUGACCUGUGUUCGGUAAUCACAUCACAGUCAGAGUUCUAGCUACACGGCGCG	Hexapoda	Yes	No	Yes	Yes	Yes	Yes	Yes
bmo-mir-12	UUCUGAGUAUUACUUCAGGUACUGGUUGUAUUAAAUAAAAAACGGAAUAAGUUAUUAACACAGCCUAAUAGAGAUAUUACGGAUUUACGA	Protostomia	Yes	No	Yes	No	Yes	Yes	No
bmo-mir-14	UUGUCAUUUGUGUCGGGGAGAGAAAUCGACGAGGCUGUUUUAUUUAGUCAGUCUUUUUCUCUCUCCUAUAUGAGUGACAU	Hexapoda	Yes	Yes	Yes	Yes	Yes	No	Yes
bmo-mir-33	UAAAGCGAGAGUGCAUUGUAGUUGCAUUGCACCGGUUCCAUUAACGUGCAAUAUGACUACAAGGCAAAUCCCGAAUUAGU	Bilateria	Yes	Yes	Yes	Yes	Yes	Yes	Yes
bmo-mir-34	AGAAUCAGGGUAGACCGCGUUGGCAGUGUGGUUAGCUGGUUGUGUAUGGAAAUGACAACAGCCACUAACGACACUGCUCCUGCGUGCACCCUAAAUCA	Bilateria	Yes	Yes	Yes	Yes	Yes	Yes	Yes
bmo-mir-92a	GGCCCGUUUGGCUGGGCAGUGACUGGCGCCAUAUUCGGUACGUGUGCGAUAUUGCACCAGUCCCGGCCUAUCCGAGCGGGC	Bilateria	Yes	No	No	Yes	Yes	Yes	No
bmo-mir-124	CAGUCCACCUCCUCGCGUUCACUGCCGGAGCCGUUAUGUAUAUUUAAAAUUCAUAAGGCACGCGGUGAAUGCCAAGAGCGGACUC	Bilateria	Yes	Yes	Yes	Yes	Yes	Yes	Yes
bmo-mir-133	AAAGCGAGAGCGUUGUUCGCUUUAGCUGGUUGACUUCGGGUCAAAUUGUCAUUUUCAUAUCAUUUGGUCCCCUUCAACCAGCUGUAGUUAACAUCUGCUUU	Bilateria	Yes	No	Yes	Yes	Yes	Yes	Yes
bmo-mir-184	GUGCAGUGACGUGCCCUUGUCAUUCUUCAGGCCCUGUGUAUUUACAACUACUGGACGGAGAACUGAUAAGGGCACGCCGUGUAC	Bilateria	Yes	No	Yes	Yes	Yes	Yes	Yes
bmo-mir-279a	ACGUCAAUUUCUUUCGAUGAGUGGAGGUUUAGUGCAUGUUUAUUUACACCAUGACUAGAUCCACACUCAUCCAUGGAAGUUGCGA	Protostomia	Yes	No	Yes	Yes	Yes	Yes	Yes
bmo-mir-285	UGUCCAUGUUUUGACAACUGUAUUCGAGUGUGUGGAAGAGUUUUAAAAAUCCUCUAGCACCAUUCGAAUUCAGUGCUCGAACGUGGUUA	Bilateria	Yes	No	Yes	Yes	Yes	Yes	Yes
