# Supplementary material for: A Burst of miRNA Innovation in the Early Evolution of Butterflies and Moths
Source: Mol Biol Evol. 2015 Jan 8;32(5):1161–74. doi: 10.1093/molbev/msv004 (PMC4408404; doi:10.1093/molbev/msv004)
Supplement: Supplementary Data [file supp_msv004_Supplement_S2.pdf]

Conserved miRNAs in *Cameraria ohridella*

Working ID		Primary sequence	miRDeep score	MFE	5p mature sequence	5' count	3p mature sequence	3' count	Maps to
Cam-034	Coh-bantam	gaagagaaacauaauauagaggaaucacgaaacugguuuucauaaugauuugacagauuguuuguauuucugagaucauugugaaagcugauuuuggucuaugucua	10520.5	-31.30	ugguuuucauaaugauuugacaga	21	ugagaucauugugaaagcuga	20322	>NODE 264450 length 2168 cov 8.011070 13279
Cam-002	Coh-let-7	aacauagucccauguuuuggugagguaguagguuguauaguacaguauuacaacacccggugaacuguauagccugcuagcuuuccuugacuggugacacuggcaacaua	5.2	-41.90	ugagguaguagguuguauag	2092013	cuguauagccugcuagcuuucc	35	>NODE 1036071 length 20211 cov 9.531591 37867
Cam-015	Coh-miR-10	cgaaaacuggcgauuucucuagugcccuacaucuacccuguagauccgaauuuguuugaaguaaugcgacaaauucgguucuagagagguuugugugguguaccacuacguc	51493.9	-38.14	uacccuguagauccgaauuugu	22763	aaauucgguucuagagagguuu	78206	>NODE 273584 length 3021 cov 9.970539 13686
Cam-086	Coh-miR-1000	caugucacucaagauaauuccagcucaauaccuguauauuguccugucacagcaguaagauacaacgcuacugcuggacggggacaauuucuggguguugcagcagaaacag	748.8	-42.90	auauuguccugucacagcagua	253	cugcuggacggggacaauuucu	1207	>NODE 309475 length 3347 cov 9.235136 15254
Cam-044	Coh-miR-11	aggaagguucgaucucguggcuagcacucgggcugugagauguguuuccuuuucacaucacagucagaguucuagcuaugcggugcgagcaucacuacagagaguaugaaaaug	5314.9	-45.20	cuagcacucgggcugugagaugug	6903	caucacagucagaguucuagcu	2682	>NODE 1499543 length 3870 cov 9.710336 44367
Cam-159	Coh-miR-1175	caugcgaacggggaccucucgcguaaaggaguaaguggaggugugaucucuucacuuuuuauuuuuaaagugagauucaacuccuccaacuuaaccccgaaacgcaauugcauc	195.4	-39.30	aaguggaggugugaucucuucacu	179	ugagauucaacuccuccaacuuaa	182	>NODE 331476 length 8192 cov 9.508911 16209
Cam-063	Coh-miR-124	ucaagaagccguaucggcagucuaucuccucgcguucacugccguugccauuauguugucuugcuauccauaaggcacgcggugaaugccaagaguugacucauucuucag	1392.4	-38.40	gcguucacugccguugccauuau	6	uaaggcacgcggugaaugcca	2717	>NODE 370250 length 573 cov 11.860384 17820
Cam-099	Coh-miR-133	uuaaagcaaaagugugagcguuguuugcuuuagcugguugacuucgggucaaauuguaacuauaaaucauuugguccccuucaaccagcuguaguugacaucugcuucgcuu	331.6	-41.40	agcugguugacuucgggucaaa	5	uugguccccuucaaccagcugu	637	>NODE 814107 length 2686 cov 9.320923 33216
Cam-061	Coh-miR-13a	cacgcguucucgggccuagccugucaaagcggccgugaaaugacacuuuuuuacauaucacagccacuuugauguggucgguuugcagagaaaaacaacgccccuccagccu	1559.5	-34.50	cugucaaagcggccgugaaaug	2852	uaucacagccacuuugaugugg	214	>NODE 363576 length 1067 cov 7.466729 17547
Cam-070	Coh-miR-13b	ccuacaagaagcgacucggacguccgaucccuucguuaaaauggcugugccaugucgaauuauaggaucauaucacagccauuuuugacgaguugggucgugcgugguaucugc	995.9	-40.50	ucguuaaaauggcugugccaug	13	uaucacagccauuuuugacgaguu	1930	>NODE 363576 length 1067 cov 7.466729 17546
Cam-065	Coh-miR-14a	gaaugaagacguuccaguuuguguucaccgugucggugagagaaauuggcucgacuuugcuuuaaaacagucagucuuuucucucuccuauaugguaagcauacguccu	1636.3	-40.90	cggugagagaaauuggcucgacu	942	ucagucuuuucucucuccuau	2129	>NODE 135070 length 3028 cov 9.523778 7269
Cam-057	Coh-miR-14b	uuauauggcgcuguaauagucgucugucguaugggaagagaaauuggcuuggcuagcucuuguguaucagucagucuuuuucucuuucuuauaugucaggcuuuaaaucuuc	2218	-34.90	gggaagagaaauuggcuuggcu	944	ucagucuuuuucucuuucuuau	3398	>NODE 323604 length 4197 cov 10.374791 15792
Cam-003	Coh-miR-184	gcguuuugaaaaugaagaguuuuugcagaacuagcccuuuucauucucucggccagugguuuuugugcgacuggacggagaacugauaagggcuggcgaugcaaauugc	205256.9	-41.80	ccuuuucauucucucggccagu	432	uggacggagaacugauaagggc	402162	>NODE 817265 length 2821 cov 8.210209 33305
Cam-043	Coh-miR-190	aaacgaagacgcccucgguaagauauguuugauauucuugguuguuuauuccacuaucacccgagaaucaaacauauuacuacugaucgcgucaccaaacuacguugaagagac	4705.4	-35.02	agauauguuugauauucuugguug	8184	cccgagaaucaaacauauuacua	263	>NODE 485962 length 353 cov 8.206799 22869
Cam-122	Coh-miR-193	ugugaagaaaaaccuacccagcuacgcuugcaucuugggucuugguggccuggucgguguuuuaauucccuacuggccugcuaagucccaaguugcaucguaacaucacu	222.5	-40.50	ugggucuugguggccuggucgg	94	uacuggccugcuaagucccaag	333	>NODE 320955 length 888 cov 8.177928 15703
Cam-001	Coh-miR-1a	agaagcaagaagaaaacagccuagcgcgaguuccgugcuuccuuacuucccauagugauuguuuagcguauggaauguaaagaaguauggagcugcgcgggcgucuac	2316367.9	-44.00	ccgugcuuccuuacuucccaua	140	uggaauguaaagaaguauggag	4543315	>NODE 510311 length 3229 cov 9.419015 23989
Cam-275	Coh-miR-1b	gauugcgugcuuggguagacgcccgcgcagcuccauacuucuuuacauuccauacgcuaaacaaucacuaugggaaguaaggaagcacggaacucgcgcuaggcuguuuucu	1.3	-38.54	-	0	ugggaaguaaggaagcacggaa	17	>NODE 510311 length 3229 cov 9.419015 23992
Cam-124	Coh-miR-210	gacugcacgaacaguaugaaguccaaagcagcugcuggccacuguacaagauuaguucuuuccuauacucuugugcgugugacagcggcuauugcgggcauuucauaaucaa	178.1	-40.10	agcugcuggccacuguacaagau	58	cuugugcgugugacagcggcuau	283	>NODE 495727 length 2254 cov 6.641526 23318
Cam-054	Coh-miR-252	acacaaacuaaaucuuguuccuaaguacuagugccgcaggaguuauucauacaucuccugcugcuuaagugcuuaucaaugaguauuuaguguuggaucggcgauggaacca	3081.2	-36.10	cuaaguacuagugccgcaggag	6032	ccugcugcuuaagugcuuauca	3	>NODE 579510 length 821 cov 8.588307 26095
Cam-011	Coh-miR-263a	caaucgcuuuaugcacaggcaauggcacuggaagaauucacggguuucuuuuccgucccguggucucuuaguggcaucacuggugcuuugcgauccuucacaacauguc	72821.8	-36.70	aauggcacuggaagaauucacgg	141452	cguggucucuuaguggcaucac	20	>NODE 718135 length 2186 cov 8.894785 30172
Cam-143	Coh-miR-263b	agauuuucagcaccaugggccuuggcacugggagaauucacaggcguuauuuaaagcccgugaauuuccugcugccuuagcucaggguugcuuuucaucaucaucgaagaug	126.7	-41.40	cuuggcacugggagaauucaca	228	ugaauuuccugcugccuuag	12	>NODE 380805 length 2024 cov 7.210474 18327
Cam-152	Coh-miR-2731	ucgaggucauagccaaacggcuuuuaucucuccauaccaggagaucgacaucuccagagguacagcccauuauuugccccggacgcaugucgcucuuuuugaguuuagu	-3.6	-29.70	-	0	uauuugccccggacgcaug	194	>NODE 406378 length 8739 cov 7.936491 19425
Cam-126	Coh-miR-274	cccuguuaugcgaaguugguuugugaccgucacuaacgggcaguuauaacauuuugcccguuuugacgaucgcaaaaugaacucgcuuaacuauaaacgucuaucggaagcau	5.1	-40.40	uuugugaccgucacuaacg	484	cguuuugacgaucgcaaaauga	7	>NODE 1055099 length 1428 cov 7.252101 38423
Cam-004	Coh-miR-275	ccagcugaacacucaacaccaaugguccggccgccgcgcgcuacuccggcgccgaggcugcccuacccagucagguaccugaaguagcgcgcgaugaccaccauucucau	181161.6	-43.40	cgcgcuacuccggcgccgaggcu	6	ucagguaccugaaguagcgcgcg	355035	>NODE 365460 length 2367 cov 11.462189 17601
Cam-008	Coh-miR-2755	uggaagauacgauauaaagaggauguggugaggcaagguggccuagcagcguguuuuuuuuauagguuugcacccugucagaccauacuuguuucaacgcaucccuuaacauc	84494.5	-39.60	caagguggccuagcagcguguu	129	cacccugucagaccauacuuguu	165590	>NODE 318035 length 1711 cov 8.439509 15602
Cam-016	Coh-miR-2756	aucaucaggguuggcgguguacccuguagcugcuuaggggcgcggcuuuaauaaccgccucuaaguuguuacauagugccccuccaucuaucagucaucgucaucguaucga	22780.6	-38.60	acccuguagcugcuuaggggcg	44704	ccucuaaguuguuacauagugc	1	>NODE 197627 length 5987 cov 10.459496 10407
Cam-010	Coh-miR-276	aagaaagaaguuuugaucuuaaaggugauuaccacuagcgagguauagaguuccuacguguccuauuuuguaggaacuucauaccgugcucuuggguuugccaaucuucuuc	48397.3	-38.60	agcgagguauagaguuccuacg	8883	uaggaacuucauaccgugcucu	86039	>NODE 646096 length 3281 cov 6.768973 28062
Cam-092	Coh-miR-2763a	aaguaaucugccauaaaagucuucaacguuucccaaaguacugaguuuaauacgucaugaaacgucacugauauuaugcucauuacuuugucuacguugcaugauaauucuuc	340.6	-26.90	ccaaaguacugaguuuaauacguc	8	auauuaugcucauuacuuugucu	649	>NODE 324782 length 1987 cov 9.921993 15840
Cam-253	Coh-miR-2763b	agaacacucaucgacguacgccaaagugguguguguaauuuaucaaaguaagaacaauuacauuaugcacccccucuugcauacguuguaugaaucuacaucaaucgaagac	25.9	-25.50	ccaaagugguguguguaauuua	32	cauuaugcacccccucuugcau	16	>NODE 324782 length 1987 cov 9.921993 15837
Cam-040	Coh-miR-2765	aagaaagacguaucuuggccugguaacuccaccaccguuggcguuacuaguccgccaacgggguuagaguuccugcucugagaugcgagacucgacgaaccaagauauccua	4851.7	-40.60	ugguaacuccaccaccguuggc	9501	caacgggguuagaguuccugcu	7	>NODE 1253774 length 8666 cov 8.462612 41405
Cam-098	Coh-miR-2768	guccguugcaauauuuugaccaauuuuaaauuucgaauggaccaaguuccaaauucgaauccugugugaauugguuaagauauugcaucgucugccuguaacggaccaau	745	-39.53	cguugcaauauuuugaccaauu	6	uugguuaagauauugcaucguc	1454	>NODE 540184 length 3939 cov 6.780655 24931
Cam-045	Coh-miR-277	cccuggagcucgugccaggagugcguuugcacagaaugcgacucaauugcuuuaugcaagguugacacuguaaaugcacuaucugguacgacacuccgggguggcuuacau	4142.6	-54.40	cgugccaggagugcguuugca	645	uaaaugcacuaucugguacgaca	7460	>NODE 509265 length 1954 cov 9.830604 23943
Cam-026	Coh-miR-278	aaaagugaagaaagaagaaguaucaagugcuacgcccggacgaacuucucagaucggccgauugaccuggucggugggaucuucguccguuuguaucgcuuauacguu	12318.6	-40.20	ccggacgaacuucucagaucggcc	4839	ucggugggaucuucguccguuu	19249	>NODE 310246 length 12610 cov 9.997701 15279
Cam-148	Coh-miR-2786	aagaugaucuucggccccugagguucugcaagcgaaggaauauuacgcccgaccguguacucucucgcuuucaugaccuugggguucgaggagaugcagcagcagcucaaga	102.4	-45.60	agguucugcaagcgaaggaaua	119	cucucucgcuuucaugaccuug	70	>NODE 634421 length 52338 cov 9.835397 27677
Cam-135	Coh-miR-2788	aaauaacgaucugcgcgugagucgccgcaaagauggggguuucucagcggcaugugccaaguaacugaucgcaaugcccuuggaaaucccaaacaugcccgugacaucuaa	245.5	-35.10	ugggguuucucagcggcaugu	157	caaugcccuuggaaaucccaaa	317	>NODE 1194383 length 8749 cov 7.684878 40559
Cam-027	Coh-miR-2796a	aagaagguagaaguaaugaguacagccagaaggggguuucuuucggccuucagcugcgaaaaaaaaugccuguaggccggcggaaacuacuugcugacuguacuuuaca	18990.9	-44.00	agggguuucuuucggccuucag	131	guaggccggcggaaacuacuugc	37110	>NODE 661793 length 608 cov 8.549342 28585
Cam-270	Coh-miR-2796b	acgaaggcaaagauggaaaagguguacagucgguaggggauucauuuuaccugcagcuuagcauugccuguaggcggacugaaugacccuacgccgacuguacuuaacacacca	5.3	-44.71	aggggauucauuuuaccugca	7	uaggcggacugaaugacccuacgc	19	>NODE 117801 length 1181 cov 16.349703 6296
Cam-019	Coh-miR-279a	gacgagggacuucggaagcgcaacaauuucuuucgaugaguggagguuuagugcauguuccuuuacaucaugacuagauccacacucauccauggaaguugcaagagucguc	20028.2	-40.70	cgaugaguggagguuuagugcaug	2253	ugacuagauccacacucaucca	36993	>NODE 661828 length 19309 cov 9.591486 28588
Cam-018	Coh-miR-279b	uuaaauaauaucaacuauaucaacugcagucagugagugucuuucuaaaugcacagguuucuccauucugugacuagaucuacacucauugauugcauuuggaccuaaaucu	22599	-37.80	agugagugucuuucuaaaugca	3	ugacuagaucuacacucauuga	44316	>NODE 1263723 length 6920 cov 8.627312 41534
Cam-035	Coh-miR-279c	caaaugcaugacacccuuagagauuuuuacgaaggcugggugcauuucuagaacauguuuuucgaagccaugacuagauuuucacucauccuccgugaaaauuugucgccug	5357.6	-32.10	gcugggugcauuucuagaacaug	106	ugacuagauuuucacucauccu	10396	>NODE 343973 length 2492 cov 7.326645 16704
Cam-017	Coh-miR-279d	gucuauuuaucuugacuugaugauauuugugggcgaguuuguuucuggugcgugugaauauaaauaaucaugacuagauccauacucgucugcagccaucaacucauucau	34857.2	-36.64	ggcgaguuuguuucuggugcgug	11	ugacuagauccauacucgucu	68351	>NODE 1263723 length 6920 cov 8.627312 41532
Cam-012	Coh-miR-281	augccuauaaucuguuaaugaagagagcuauccgucgacaguauugacaguaaacacugucauggaguugcucucuuuaugaacggaugcuggcccagcaguaugauauucg	48376.2	-44.40	aagagagcuauccgucgacagu	94723	cugucauggaguugcucucuuu	31	>NODE 260640 length 6147 cov 7.778103 13144
Cam-093	Coh-miR-282	caagaaacaggacguuaaccuagccucuccuuggcuuugucuguuauuucaaacuucagacauagccuaaaagagguuacguuuaguccuuaacauuuaacaucuaugaaga	1046	-35.62	uagccucuccuuggcuuugucu	1605	acauagccuaaaagagguuacg	420	>NODE 1048779 length 2380 cov 6.818487 38244
Cam-146	Coh-miR-283	guauuguaauuuuuuuuuaauguggggauaucacaaaauaucagcugguaauucugggcauagacaacacccggauugcucgcugguaucacgugguguuucacuaugaccgu	91.8	-38.50	aaauaucagcugguaauucu	74	cggauugcucgcugguaucacg	98	>NODE 175783 length 5559 cov 9.554236 9280
Cam-025	Coh-miR-285	ugaagaccugaaaaccccauguauuugacaacugcauucgagugaguggauagaguuugaaucaauuaucuagcaccauucgaauucagugcucgaaucugggauauc	8933.8	-33.70	acugcauucgagugaguggauaga	4	uagcaccauucgaauucagugc	17512	>NODE 408592 length 952 cov 8.781512 19537
Cam-047	Coh-miR-2a	ugaagaggcgguuaaugauagaugcagccgcgccaucaaagucgguuuguuauagaucauugagcaacuaucacagccagcuuugaugagcauggcugcauuguaccaga	9509.5	-41.50	ccaucaaagucgguuuguuaua	506	ucacagccagcuuugaugag	18138	>NODE 363576 length 1067 cov 7.466729 17550
Cam-358	Coh-miR-2as	gcgcauucccagcacaguagucaacaaagcuggcugugauaugaacuuaguaacaugucacauccacuucgucgaccacucgcacggacauucucuccggaaaucuuucaaa	6.1	-28.50	ucaacaaagcuggcugugauau	6	ugucacauccacuucgucgacc	1	>NODE 363576 length 1067 cov 7.466729 17540
Cam-048	Coh-miR-2b	augaagacguaagaagaaauaggccucgcgccgcucucaaagugguugucauauguguacuuguaucauaucacagccagcuuugaugagcgcugcgaggcgcuacugca	5.5	-49.10	cucucaaagugguugucauaug	177	ucacagccagcuuugaugag	10754	>NODE 363576 length 1067 cov 7.466729 17544
Cam-007	Coh-miR-2c	gagaauguccgugcgaguggucgacgaaguggaugugacauguuacuaaguucauaucacagccagcuuuguugacuacugugcugggaaugcgcaacaaugaagacucuug	80564.7	-39.30	ucgacgaaguggaugugacaug	156767	uaucacagccagcuuuguugacu	1192	>NODE 363576 length 1067 cov 7.466729 17541
Cam-056	Coh-miR-305	cacgaagauuggcaaaccccucgucgcacgcccauuguacuucaucaggugcucuggugaugguauuuccaggcgcuuguuggaguacacuuaccgugucggcgauaggcug	6656.7	-44.90	auuguacuucaucaggugcucugg	5822	aggcgcuuguuggaguacacuua	7174	>NODE 365460 length 2367 cov 11.462189 17603
Cam-033	Coh-miR-306a	gacaguagcccgcccccauaucagguacuaggugacucugaguggcugguuugucucagagaugccuugugccuggauaugggugguagcuaaucauccaccacuuggauca	8763.9	-48.60	ucagguacuaggugacucuga	16575	agagaugccuugugccuggaua	606	>NODE 498062 length 5073 cov 8.557461 23415
Cam-230	Coh-miR-306b	gaugauuagcuaccaccauauccaggcacaaggcaucucugagacaaaccagccacucagagucaccuaguaccugauaugggggcgggcuacugucgcguucucucuuca	5	-39.00	uccaggcacaaggcaucucugag	40	-	0	>NODE 498062 length 5073 cov 8.557461 23414
Cam-055	Coh-miR-307	ugaaaggacaacucguuuccauugacucacucaaccugggugugaugugugcacucggugcucggcccaucacaaccuccuugagugagcgaauguggacgagacacgauua	3016	-44.60	acucacucaaccugggugugaug	36	ucacaaccuccuugagugagcga	5862	>NODE 679693 length 2205 cov 6.832653 29098
Cam-014	Coh-miR-308	uggaagauacauguuaagaugaguguaguuuccuuucgugguauucuuccugugaaugugagaugacuacaaucacaggagaauacugcgaggggcagcuaccuauuaac	28769	-48.70	cgugguauucuuccugugaaugu	24	aaucacaggagaauacugcgag	56396	>NODE 509545 length 7981 cov 9.709310 23961
Cam-009	Coh-miR-31	uuuuaagccuauggcuggggaaggcaagaugucggcauagcugauugaauauuguaugcggcugugucacaucgagccagcucaauccacugguguucaacuaaaacaagaa	4.9	-37.46	aggcaagaugucggcauagcu	154208	ggcugugucacaucgagccagc	6	>NODE 423848 length 3233 cov 8.770802 20227
Cam-196	Coh-miR-316	ugugaaguucugcauagauuugucuuuuuccgcuuugcugcugauugauuaaaauugucaacggcagagcgaaaaagucuccuuuaugagaacugucuccggaacugcuuaa	54.9	-36.20	ugucuuuuuccgcuuugcugcu	74	acggcagagcgaaaaagucucc	26	>NODE 1676125 length 5537 cov 7.432906 45836
Cam-037	Coh-miR-317	caagacagaagacgccggacuauugaacgaccgggugccacgcugugcucucucgguucccauguucgagugaacacagcuggugguaucucaguuguucuguaguucucacc	12543.2	-47.50	cgggugccacgcugugcucucu	40	ugaacacagcuggugguaucuca	20010	>NODE 497570 length 2281 cov 8.804033 23378
Cam-252	Coh-miR-3286	cagaugaagacgugaaacccaaaacaacuauuggucacugcacaugaguagagaaggaucaaaugcuaucuugugcguguuccaacaguuauuguggguugaaacaauaucg	11.5	-31.50	aacuauuggucacugcacauga	1	uugugcguguuccaacaguuau	15	>NODE 117801 length 1181 cov 16.349703 6294
Cam-105	Coh-miR-33	gaagauugacaacuguggaagugcauuguaguugcauugcacuuguuugguuuauugugcaauaucacuacaaggcaacuuuugcggcugucgcauucaucauaugaaaga	249.9	-38.24	gugcauuguaguugcauugca	470	caauaucacuacaaggcaacuu	12	>NODE 245179 length 1317 cov 12.226272 12461
Cam-371	Coh-miR-3327	gcacccucaagaagauuuaggguaauguauaaacaacaggaauguuauguacaguauaaugugagcucuauauguaacguuuuuguugucuuaaauuucucuccacucauca	9.8	-24.92	aacaacaggaauguuauguaca	7	auauguaacguuuuuguugucuu	10	>NODE 242931 length 296 cov 18.226351 12362
Cam-021	Coh-miR-34	gaagaaacgguaaaccgcguuggcaguggugguuagcugguuguaauggcucaauauacagccucuagcgacacugccucugcguuaaccuaauuuaucuacuucgcaucc	5.1	-40.50	uggcagugugguuagcugguu	31296	cagccucuagcgacacugccucu	196	>NODE 598335 length 640 cov 12.315625 26586
Cam-094	Coh-miR-375	uaauuuggcagugugagccgacccgagcgguaugagcaaacuuugaauuuugaacguuaguuuguucgccccggcucgugucgguuaaugcugcgcaagcgacgauuuacca	478.2	-46.29	acccgagcgguaugagcaaacu	622	uuuguucgccccggcucgugu	307	>NODE 716789 length 3949 cov 6.887313 30130
Cam-096	Coh-miR-6094	auuuguuaucgucaucgguggucuggggguaccuccagucugcccaacccugaguuugcuggguuacaugguauucgagaccucugcugauccuggaccuugccuuuuuu	427	-35.30	gucaucgguggucuggggguac	116	uauucgagaccucugcugauc	714	>NODE 1446069 length 5040 cov 9.174802 43785
Cam-095	Coh-miR-7	gaaacccgccgcgcguuguauggaagacuagugauuuuguuguuaguuaucauuaaacaagaaaucacuaaucucccuacaaagccuggcaaucucuucgugggaccc	619.8	-35.60	uggaagacuagugauuuuguuguu	1192	caagaaaucacuaaucucccua	9	>NODE 508182 length 1129 cov 7.262179 23910
Cam-080	Coh-miR-71	aauuuaaaauggaaaagcuuguugcgguuuggaagacaugaguagugagauguccaugaacaucgcccguucucacuaccuugucuuucaugcggcuccagcuaacgucauc	1608.4	-36.90	uggaagacaugaguagugagaug	955	ucucacuaccuugucuuucaug	2176	>NODE 465018 length 531 cov 5.201507 21998
Cam-062	Coh-miR-745	aauagauagaaguugugaaccucgguucugcuggcgcuccucguugggcagguugcuggcuugagaucgcgagcugccuaucgaagggcuacggaagacugcagguguccuc	1809.9	-41.10	gcgcuccucguugggcagguu	143	gagcugccuaucgaagggcuac	3404	>NODE 106986 length 245 cov 8.906122 5698
Cam-066	Coh-miR-750	gacaguucagugaccgucugccagaucuaacuuuccagcucacgcguggagucaugucuccgucuguuuggguuucaaagucaauaucgccaacaucaugcgaacggg	-1.4	-25.80	ccagaucuaacuuuccagcuca	2220	-	0	>NODE 331476 length 8192 cov 9.508911 16208
Cam-073	Coh-miR-79	gaggugaucuaggaaguaaugcgcguugcgggcucuuuggaaauuuagcucucgugacguuucccugcucauaaagcuagauuaccaaagcacucucucgcgcuuaagc	862	-33.60	cuuuggaaauuuagcucucguga	2	auaaagcuagauuaccaaagca	1689	>NODE 214814 length 1653 cov 13.555959 11219
Cam-013	Coh-miR-8	gccccgccgcacacgcaggagacucgguucgcaucuuacagggcagcauuagagucuguuuacguauaucuaauacugucagguaaagauguccuccgaucuccucuuucg	41237.7	-37.50	caucuuacagggcagcauuaga	17275	uaauacugucagguaaagauguc	63530	>NODE 714109 length 2385 cov 13.141300 30023
Cam-064	Coh-miR-87	aucaauucaugguaauaucugguacacugaugggccugaaauguugcucugaccugugcccuuuaucuaggugagcaaacuuucagguguguuggugccgauuguucaa	1400.8	-35.30	gggccugaaauguugcucugaccu	5	gugagcaaacuuucaggugug	2735	>NODE 72354 length 512 cov 6.746094 4021
Cam-106	Coh-miR-927	uaauggagugccgagaugccaauuauuccauuuuagaauuucuacgcuuuaccguugccaaguauuugggcaaagcguuuggauucuaaaacgggauuugugguucaac	4.7	-35.30	-	0	caaagcguuuggauucuaa	641	>NODE 1261864 length 1140 cov 4.406140 41514
Cam-212	Coh-miR-929	gacaaaaggcgucgggggccaaauugacucuaguagggaguccgugauuauacgcgacuuccuaaucgagucagguugacuucugugagccgcauucuucuagaugaugaa	21.8	-43.10	aaauugacucuaguagggagu	28	cuuccuaaucgagucagguuga	6	>NODE 324102 length 1057 cov 8.578051 15801
Cam-039	Coh-miR-92a	gaucagucuucuuuguccagggggcccguguggaugggguggugacuggggcuauauucagauauuggcgauauugcaccagucccggccuaucugcgcgggcccugucg	6225.7	-55.20	ggguggugacuggggcuauauu	2627	uauugcaccagucccggccuau	9575	>NODE 488307 length 9888 cov 9.621258 22977
Cam-071	Coh-miR-92b	gucggucguuacuccgcgguggucgugcuuaggacgcgauuugguguaaccguugugcuuuuccgauaacuauugcaccaaucccggccugccugcggccaccgcaucca	2024.5	-46.00	aggacgcgauuugguguaaccgu	1330	uauugcaccaaucccggccugc	2632	>NODE 488307 length 9888 cov 9.621258 22979
Cam-138	Coh-miR-932	cugcuuggguagccaagaccucaauuccguagugcauugcagugcauaaggccaccugcaagcagugcggaagugagguggaggcaaccgauuucaacauuaacgaaagaa	5.3	-45.50	ucaauuccguagugcauugca	248	ugcaagcagugcggaagugagg	78	>NODE 727572 length 3543 cov 9.047418 30466
Cam-103	Coh-miR-965	ggaauacacugggcgacugaagggaaaaguuguaacgccuuauguuguacccauuuaucauaagcguauagcuuuuccccuuuguagcucaguuaaugcaccacauuauuugga	389.6	-38.80	agggaaaaguuguaacgccuuaug	535	uaagcguauagcuuuuccccuu	219	>NODE 354454 length 3143 cov 11.319122 17225
Cam-020	Coh-miR-970	aaugaagagagugagugagugaucggggagccuggcgggugaucuuauugguagugugaugaugaaucuaucauaagacacacgcggcucucugcaucaucucccgaucug	14833.1	-42.00	gugagugagugaucgggg	27	ucauaagacacacgcggcucu	29062	>NODE 435267 length 1614 cov 4.978934 20766
Cam-112	Coh-miR-971	ccaguuuggggugcacgguccacugaaagauauugaacaucaagcacaagcuggcuugguguucuaccuuacagugaacugagcuccacauuauucagcuacgauuuaugggcc	4.9	-38.10	cacugaaagauauugaacaucaag	12	-	0	>NODE 801328 length 1329 cov 5.732130 32806
Cam-174	Coh-miR-981	agcgaggacucagaagauauccggacaugauucggguuucgcgacgacagaaccguuaaauggaaguagguucguugucgacgaaaccugcaaaauguccggugaaacuacu	5.3	-45.44	-	0	uucguugucgacgaaaccugca	94	>NODE 457747 length 967 cov 9.188210 21702
Cam-006	Coh-miR-989	ucaaaaugcgaagaagucgagguucgaaauggccauuaccucacaaucacgugcgugucaaaguaucaacgugugaugugacguaguggaaguaucguuccgacuguuuc	134920.5	-36.50	gccauuaccucacaaucacgugcg	123	gugugaugugacguaguggaag	264342	>NODE 352054 length 1750 cov 8.401714 17111
Cam-038	Coh-miR-993	gaagauaaauuguuggcacauucgugaucuacccuguagauccgggcuuuuguaguaaugcaccuagucagaagcucgucucuacagguaucucacggcuggaagaccucgcu	14141.7	-39.50	uacccuguagauccgggcuuuu	17128	gaagcucgucucuacagguaucu	10586	>NODE 712819 length 1190 cov 8.085714 29962
Cam-085	Coh-miR-998	uggugguagggugugaaaggaaugacgggaggcgcggugagguguaucccguguggcugcauugaccuuguagcaccaugggauucagcucacugcccaucccacagcgac	1372.1	-50.90	aaggaaugacgggaggcgcggug	1	uagcaccaugggauucagcuc	2681	>NODE 1499543 length 3870 cov 9.710336 44366
Cam-049	Coh-miR-9a	gaaaacccaguuggcccgccucuuugguauccuagcuguagguguugccacguucuaccuaaaguuaugguaccgaagucccgagcuaacucacauucugcuccgcaaaua	4029.1	-36.90	ucuuugguauccuagcuguag	7267	uaaaguuaugguaccgaagucc	628	>NODE 444138 length 2220 cov 7.668919 21069
Cam-023	Coh-miR-9b	ugucguagauugguuaauuaucuuugguuaucuagcuguaugaguauuugacaucauaaagcuagguuaccggaguuaaguaccgucuaacucagcgaacaacaagcaaaac	27040.9	-34.90	ucuuugguuaucuagcuguauga	43136	auaaagcuagguuaccggaguua	9894	>NODE 70099 length 404 cov 11.284654 3898
Cam-205	Coh-miR-9d	agaguaccagccgaaaauagcaccauacuuauuuggugggcuagcuuaugaauguuccauuuucuaugucauaaagcuaucacaucaaauugauauggugugcauaagacag	35.4	-27.40	auuuggugggcuagcuuauga	37	auaaagcuaucacaucaaauug	28	>NODE 1514790 length 1505 cov 6.042525 44565
Cam-076	Coh-miR-iab-4	gaaaacgcgucgcuuucuguacguauacugaauguauccugagugccucauucuuuccgguauaccuucaguauacguaacaguaggcgacacuugacaucgaugggaaga	920.5	-42.40	acguauacugaauguauccuga	1607	cgguauaccuucaguauacgu	190	>NODE 250371 length 8284 cov 9.429985 12746
Cam-207	Coh-miR-iab-8	augucaagugucgccuacuguuacguauacugaagguauaccggaaagaaugaggcacucaggauacauucaguauacguacagaaagcgacgcguuuucuucaugacucuuc	5.1	-40.22	uuacguauacugaagguauaccg	59	aggauacauucaguauacguaca	11	>NODE 250371 length 8284 cov 9.429985 12747
Cam-042	Newly discovered	caguuuuugucaucgaaaugucagccccauuugcugugacucuaacaucuaauagguuauugaaucacuacuagguguuagggucacagcgaaugugcgugacguaac	5714.1	-48.70	gcugugacucuaacaucuaaua	568	cuagguguuagggucacagcga	10638	>NODE 107182 length 1282 cov 11.429797 5701
Cam-110	Newly discovered	caacgugcauauuuuuuucnnnnnnnnnncacggguagucauuuugcuagucgauagucccaugauacuaucacugggaaaaugauaccacgccuuauuugacaaaaacgu	136.6	-25.60	gguagucauuuugcuagucgaua	3	ucacugggaaaaugauaccacg	262	>NODE 1533624 length 3047 cov 7.091237 44831
Cam-154	Newly discovered	auuuuaaaagaaauuacugguuuacugaguagcaaucauuuacuuaguagacauuuacauuccuacuaugucacugggugaaugaugccacuucuguaaacugaaacuac	103.7	-34.22	agcaaucauuuacuuaguagacau	97	ucacugggugaaugaugccac	99	>NODE 10635 length 398 cov 6.746231 658

Conserved miRNAs in *Pararge aegeria*

	Blastn miRBase20	Putative primary sequence	miRDeep score	MFE	Putative -5p	5' count	Putative -3p	3' count	Maps to
Par-052	Pae-bantam	agucaagaaagaagagaucaggaauuacgaaacugguuuucauaaugauuugacagaugguuuuauauucugagaucauugugaaagcugauuuuguuccuaucguucu	3648.1	-35.10	ugguuuucauaaugauuugacaga	16	ugagaucauugugaaagcugauuu	6718	>NODE_556918_length_20507_cov_8.602428_44831
Par-003	Pae-let-7	ggcauaagccgccggcgggggugagguaguagguuguauaguacugcauuacaccacauaggagagacuguauagccugcuaacuuucccugacggcggcagagcgacgug	5.6	-52.72	ugagguaguagguuguauag	471768	cuguauagccugcuaacuuucc	10	>NODE_552053_length_7825_cov_9.408946_44647
Par-001	Pae-miR-1	uacaagagagaagacacauagccuugcgcuaguuccgugcuuccuuacuucccauaguauuaaauucauauggaauguaaagaaguauggagcugcgcugguaugccggcgu	821679.8	-40.60	ccgugcuuccuuacuucccaua	41	uggaauguaaagaaguauggag	1611620	>NODE_475318_length_18295_cov_8.714786_42328
Par-216	Pae-miR-1000	uugcgcguccccgccauauuguccugucacagcaguacgugauacuacugcugcgccgggacaagccggcgguggcgucgcccaccaccagcagcccgaagauacca	48.9	-47.20	auauuguccugucacagcagua	106	cugcugcgccgggacaagccgg	19	>NODE_182425_length_4030_cov_8.463524_23850
Par-009	Pae-miR-10a	aaggagaaugucaucaaaauuagugcccuacaucuacccuguagauccgaauuuguuugaagugaggcgacaaauucgguucuagagagguuuguguggugcaugcacggcga	39878	-37.44	uacccuguagauccgaauuugu	16605	caaauucgguucuagagagguuu	61582	>NODE_197924_length_7971_cov_7.477857_25343
Par-090	Pae-miR-11	cuugcauugaagaacgggaaucuucgcuccuuguggcuaguacucgagacugugaucuguguaaccgucacaucacagucagaguucuagcuacacgcugcgaacucacuuc	725.4	-44.50	cuaguacucgagacugugaucug	3	caucacagucagaguucuagcu	1409	>NODE_186966_length_3532_cov_6.613817_24266
Par-200	Pae-miR-1175	agaagaagagagauuacuagcguuaaggauuaaguggaggugugaucucuucacuuuuuaugcuuaaagugagauucaacuccuccaacuuaacccugacucgcaauuucauc	162	-36.00	aaguggaggugugaucucuuc	84	ugagauucaacuccuccaacuuaa	226	>NODE_146393_length_5872_cov_8.470367_20518
Par-101	Pae-miR-12	cuuaccuuuuuacccccccauccaguagaagaaguagauagaagauccucguagaaugccgcauuguuucugaguauuacuucagguacugguggaaugauauauaaug	-4.8	-21.00	-	0	ugaguauuacuucagguacugg	849	>NODE_397723_length_8295_cov_8.728993_38835
Par-072	Pae-miR-124	gugaagauagaagucaagaguccgucuccucgcguucacugcauugccucuuauguuuuuauauuagucauaaggcacgcggugaaugccaagagcggacucaagcaac	5.2	-43.52	-	0	uaaggcacgcggugaaugcca	1265	>NODE_17397_length_3819_cov_6.929301_3793
Par-073	Pae-miR-13a	cucgcguucucgggccuagccugucaaagcggcaugugacauguuuucaguuuucauaucacagccacuuugauguggucgguuuacugagaccaucuuauugauacacuuaa	819	-34.70	cugucaaagcggcaugugacaug	1379	uaucacagccacuuugaugugg	155	>NODE_134367_length_3129_cov_5.797699_19357
Par-121	Pae-miR-13b	uuaacgaagauuuccggggcucagguccguggcccuaucguaaaaauaguugugcaauggucgucuuucauaucacagccauuuuugacgaguugggcugcgugccguauguua	177	-38.50	ucguaaaaauaguugugcaaug	13	uaucacagccauuuuugacgaguu	320	>NODE_134367_length_3129_cov_5.797699_19356
Par-136	Pae-miR-133	gcggcgugaaaguguuagcguuguucgcuuuagcugguugauuucgggucaaauuguacucuacuaucauuuggucccuucaaccagcuguaguugacaucugcuuugcuu	146.5	-39.90	agcugguugauuucgggucaaau	38	uugguccccuucaaccagcugu	241	>NODE_172805_length_2528_cov_6.099684_23052
Par-446	Pae-miR-137	agauugugaagaaaauucaaacuugaaggucacgcguauucuuggggaauuaacacauuuuacgagauguuauugcuugagaauacacguagcugacgaggagaaucucauc	15.3	-33.10	acgcguauucuuggggaauuaaca	6	uauugcuugagaauacacguag	17	>NODE_157584_length_4404_cov_7.926431_21659
Par-005	Pae-miR-184	acagaagaagagaagagaaguauacagcgaugggcccuugucauucuucuugcccaguguauuaagcuacuggacggagaacugauaagggccuguccuguaucauaucaac	69797.6	-46.50	ccuugucauucuucuugcccagu	136884	uggacggagaacugauaagggc	12	>NODE_396734_length_3228_cov_7.505266_38786
Par-039	Pae-miR-190	aaaaaagccacgugucgguaagauauguuugauauucuugguuguuuuuuguaucacccgggaaucaaacauauuaccuaccgcucguggcgcauucaucugaagacaag	2399.8	-38.80	agauauguuugauauucuugguug	4166	cccgggaaucaaacauauuaccua	85	>NODE_45520_length_1458_cov_7.674211_9066
Par-192	Pae-miR-193	gaagaaaagaaagaguagaagucccaaccuuagcgagggucuuggcggucuagugggugugcucaguucuuacuggccugcuaagucccaagcugguggugagacagaacgcc	97.6	-41.00	agggucuuggcggucuagugggg	57	uacuggccugcuaagucccaag	126	>NODE_602129_length_11373_cov_7.926493_45642
Par-113	Pae-miR-210	acaucaagaugaaguugaaugaaguccaaagcagcugcuggccacugcacaagauuaguuagagcauacucuugugcgugugacagcggcuauugcgggcguuucauaaacga	266.4	-41.70	agcugcuggccacugcacaaga	11	cuugugcgugugacagcggcuau	502	>NODE_318834_length_20562_cov_8.469410_34250
Par-031	Pae-miR-252	uuauaaacuaaaucauguuccuaaguacuagugccgcaggaguaauuuguguaucuccugcugcuacagugcuuaucaauagauuuagugaaacaacguuggugaaga	3135.6	-34.60	cuaaguacuagugccgcaggag	6130	ccugcugcuacagugcuuauca	10	>NODE_425521_length_2157_cov_7.861845_40102
Par-010	Pae-miR-263a	aacucgcaccaugcacaggcaauggcacuggaagaauucacggguuccuuuauuaaucccguggucucuuaguggcaucacuggugcugggcgauacuucgauuuaugagacaa	27388	-40.92	aauggcacuggaagaauucacggg	53124	cguggucucuuaguggcaucac	31	>NODE_129983_length_7531_cov_8.059487_18833
Par-343	Pae-miR-263b	aaaagaaggcuauguugggucuuggcacugggagaauucacaggaguuauauaauuacccgugaauuuccugaugccuuagcccacuguugccgcugucgucagagaag	5	-39.10	cuuggcacugggagaauuca	54	-	0	>NODE_74647_length_1733_cov_7.188113_12936
Par-382	Pae-miR-274	uuauuuguuagcgaaguugguuuugugaccgucacuaacgggcaguaacgacuauuugcucguuuugacgaucgcaaaaugaacucgcuugacuaauuuucgacgcuucagggg	36.6	-36.80	uuugugaccgucacuaacgggca	50	ucguuuugacgaucgcaaaaug	14	>NODE_162937_length_1365_cov_9.059340_22130
Par-006	Pae-miR-2755	uaaagaaguaaucaagaauaaggggcugguggcacaagguggccuagcagcguguuuucaauaaacuuugcacccugucagaccauacuuguucuaacaccccuauuuucaa	28769.6	-34.54	caagguggccuagcagcguguuu	7	cacccugucagaccauacuuguu	56415	>NODE_130317_length_1694_cov_8.417946_18871
Par-016	Pae-miR-2756	agagcugaggccaguuggggacccuguagcugcuuaggggcgguguuugacccucuccugagcugcuacaucguuccucgcugugaccugcaaucgucgcuacguccugcaa	13513	-49.30	acccuguagcugcuuaggggcg	26452	cuccugagcugcuacaucguuc	37	>NODE_103737_length_5270_cov_7.928083_16151
Par-012	Pae-miR-276	aucgaaacgccuucaucgaccuggugauuuccacuagcgagguauagaguuccuacguaauacaugccuguaggaacuucauaccgugcucuugguuuugccaaucuuc	18593.4	-38.20	agcgagguauagaguuccuacg	3756	uaggaacuucauaccgugcucu	32705	>NODE_329857_length_15256_cov_7.884701_34867
Par-037	Pae-miR-2765	cuucggaacguaucugggccugguaacuccaccaccguuggcgugaccucgccaacgggggcagaguucccucuccuagacgcgauguucuccugguacaagagcugaag	2423.8	-48.30	ugguaacuccaccaccguuggc	4744	caacgggggcagaguucc	1	>NODE_230511_length_5869_cov_7.952121_28590
Par-007	Pae-miR-2766	gagagauugaagauuauugagcaaacauuugaugcacccgccauucuucucgacugacguuaucuaaucaucagucuugucgaaugguggguggucuaauguucaau	5.1	-40.60	ccgccauucuucucgacugacg	116	ucagucuugucgaauggugg	88001	>NODE_618393_length_4356_cov_7.159320_45949
Par-008	Pae-miR-2767	aauauauuuuauugucguuucaaguaaaucucgugcggcuuguguuuaauucacgccguaagagauuuauuuaaaucgaugagaaaaaugcaccuguaaauuaauuaaaa	20415.5	-30.70	caaguaaaucucgugcggcuug	39974	cgccguaagagauuuauuuaaa	3	>NODE_87035_length_3878_cov_8.478598_14402
Par-106	Pae-miR-2768	gucaacgaguacccgugaggcggucggugcaauauuuugaccaauuuuauaauaaacuaguaguguaugaauugguuaagauauugcaucguccgcuuuaugggcaugccuu	5.6	-55.00	ugcaauauuuugaccaauuuuau	1	auugguuaagauauugcaucgu	451	>NODE_583490_length_9116_cov_8.291246_45305
Par-042	Pae-miR-277	cucgggaugcgugccaggagugcguuugcaaagcacgcgacgaguuugcuuuauggcaacguugacacuguaaaugcacuaucugguacgacaccccgggauguguaucgacg	2358.6	-52.50	cgugccaggagugcguuugca	88	uaaaugcacuaucugguacgaca	4528	>NODE_123219_length_9231_cov_7.857004_18162
Par-024	Pae-miR-278	augaagaaagaagagcaguaucaagugcuacgcccggacgaacuuuccauguucggccgauauauacgggucggugggaucuucguccguuuguaucgcuuauacaugua	4851.2	-40.90	ccggacgaacuuuccauguucggc	531	ucggugggaucuucguccguuu	8534	>NODE_199832_length_1571_cov_8.899427_25525
Par-244	Pae-miR-2788	uccugaauauauguggaaaaagggccgccguauagcugggguuuccuagcgguaugugccucccaucaugcaaugcccuuggaaaucccaaacgugcuggugacuauaaccc	58.9	-35.40	uggggguuuccuagcgguaugugc	21	caaugcccuuggaaaucccaaa	84	>NODE_602129_length_11373_cov_7.926493_45640
Par-015	Pae-miR-279b	uauaaaaagauaacaaggcuuucuucuggucagccaauggguuuaaaucugguggcacaguguucaccugugacuagaucuacacucauugauugcucggaggaugau	15645.3	-35.30	aauggguuuaaaucugguggcaca	238	ugacuagaucuacacucauuga	30442	>NODE_200190_length_3872_cov_7.435434_25555
Par-018	Pae-miR-279a	ggauucugcgaccggcguaaaagucaauuucuuucgaugaguggagguuuagugcauguuuuuuucaucaugacuagauccacacucauccauggaaguugcgaugcu	9221.8	-38.70	cgaugaguggagguuuagugcaug	1672	ugacuagauccacacucaucca	16396	>NODE_102121_length_7324_cov_8.694566_15979
Par-020	Pae-miR-279d	agacuacaagcuuugacuugaugauauuugugggcgaguuugcuucugguucauguuuuugaugucaucaugacuagauccauacucgucugcagccaucaacucaau	13832.6	-38.30	gggcgaguuugcuucugguucau	4	ugacuagauccauacucgucugc	27118	>NODE_200190_length_3872_cov_7.435434_25557
Par-083	Pae-miR-279c	cacauugacugcaugaguuuauggaugacuucggaggaugaguggauuucuaguaucaaguugcacuucuugacuagauuuucacuuauccuucgcgguugucgcgcccugc	437.9	-45.50	gaugaguggauuucuaguaucaag	18	ugacuagauuuucacuuauccu	829	>NODE_232890_length_3754_cov_6.983218_28742
Par-057	Pae-miR-2796	aaagagaagagagagaagaauacgagugcuaucagcagggguuucuuucggccuucagcuauuaaaaagacuguaggccggcggaaacuacuugcugauguuacucucugucuac	2556.9	-49.71	aggggguuucuuucggccuuca	19	guaggccggcggaaacuacuugc	4987	>NODE_396979_length_2536_cov_7.032729_38806
Par-022	Pae-miR-2797a	aacuauauuuuguuguucgccuaaguagauuuuguacgacuuguuucuaacccgagucguuccgauuuugcuuaaguuuacaaugaaaccaaaucaucauacuuaucaaaa	4858	-25.50	cuaaguagauuuuguacgacuu	9502	gucguuccgauuuugcuuaagu	1	>NODE_123318_length_2615_cov_7.998088_18182
Par-045	Pae-miR-2797b	caagcaacuuugcgguuuauauaaguagauuuuguccgauuuuuauucauaacaaaaucagacaaagucuaccuguauaugcggcaauagucucaacguggaauacaauauc	3140.1	-33.30	auaaguagauuuuguccgauuu	6091	aucagacaaagucuaccuguau	29	>NODE_123318_length_2615_cov_7.998088_18184
Par-061	Pae-miR-2797c	uuugaauuuugcuggugaauuuaaguagaugccauccggcuuguuuugguauaccaagcggaaugggucuauuuaaauccacccaaaauguaucuucagauguggaccuu	1025.2	-37.20	uuaaguagaugccauccggcuu	1962	gcggaaugggucuauuuaaau	40	>NODE_123318_length_2615_cov_7.998088_18178
Par-014	Pae-miR-281	aagccaauaaucuguuaaugaagagagcuauccgucgacaguauugacauuaaacacugucauggaguugcucucuuuaugaacggauguggguucaacaaaaacaagagg	24507.7	-45.00	aagagagcuauccgucgacagu	47879	cugucauggaguugcucucuuu	22	>NODE_104908_length_2152_cov_8.167286_16296
Par-092	Pae-miR-282	gaagaauugggacguugaccuagccucuccuuggcuuugucuguaucuuuaugaucuuucagacauagccugauagagguuacguuuugucccuuucacuccucaauggugc	439.3	-42.70	uagccucuccuuggcuuugucu	712	acauagccugauagagguuacg	140	>NODE_57072_length_9740_cov_8.974333_10774
Par-205	Pae-miR-283	uuucgacaaaguagcgugguguacggaauauggcuaaauaucagcugguaauucuggggguaguuggcccccaggcuaucaguugguaucacgcuagacuccgcacugcuuc	80.4	-44.00	aaauaucagcugguaauucu	68	caggcuaucaguugguaucacg	88	>NODE_397723_length_8295_cov_8.728993_38829
Par-056	Pae-miR-285	aaccgaagcauaaccacggagccauaauuugacacugcauucgagugugugggguagaguguucaauuaucuagcaccauucgaauucagugcucgaauguggacgcucgacg	1280.1	-37.00	acugcauucgagugugugggua	10	uagcaccauucgaauucagugc	2493	>NODE_147664_length_8129_cov_9.519990_20608
Par-038	Pae-miR-2a	caauaaugaaugcggccuugccaucaaagucgguuugucauagaucauaaugaaccuaucacagccagcuuugaugagcacggcuacaugucuaucaucaguugccaacgga	5094.5	-35.50	ccaucaaagucgguuugucaua	4466	ucacagccagcuuugaugagca	5513	>NODE_134367_length_3129_cov_5.797699_19359
Par-081	Pae-miR-2b	gaaaauucgcacguugaaucgacgucgagugccgcucacaaagugguuguguuauguuaaauauccauaucacagccagcuuugaugagcgacaaucggcgaccuagcguc	1121.5	-43.90	cucacaaagugguuguguuaug	8	uaucacagccagcuuugaugagcg	2175	>NODE_134367_length_3129_cov_5.797699_19354
Par-033	Pae-miR-2c	aacaaggagcaauuagggagccgauaaagugguugugacguguauguauccucauaucacagccagcuuuguugacuucauuguugguuccaaucauaguucaaguuuuaug	5283.3	-37.30	ccgauaaagugguugugacgug	5750	uaucacagccagcuuuguugacu	4436	>NODE_134367_length_3129_cov_5.797699_19351
Par-291	Pae-miR-3000	aguaguagacuuuuaguucguagcaccuagaugaaagcacaaaauguuauaagcauuuguguucaccauuccggugcuaaaucagugcuuuucaaagugcagagugcagguucua	0.4	-30.90	uagcaccuagaugaaagcacaaa	46	-	0	>NODE_470371_length_9110_cov_8.792207_42033
Par-130	Pae-miR-307	aaauaagaagaacucauucccaguuacucacucaaccugggugugaugugugcauccuaugcucccccaucacaaccuccuugagugagcgacugugguugagaugucuacg	216.1	-44.20	acucacucaaccugggugugaug	74	ucacaaccuccuugagugagcga	341	>NODE_366300_length_58598_cov_9.109031_37424
Par-036	Pae-miR-308	nnnnnnnnnnnnnnnnggaacgccggcgucuccccgcgcgguauuaguucugugaauguguuuccaccacaaucacaggauaauacugcgaggggauucgccgacucucgcc	2751.2	-51.80	cgcgguauuaguucugugaaugu	24	aaucacaggauaauacugcgag	5361	>NODE_369834_length_3080_cov_8.217532_37762
Par-011	Pae-miR-31	aaaaaagccgguggcugggaaggcaagaugucggcauagcugauugacugucauauacggcugugucacaucgagccagcucaauccaccggcguucgacgugagaac	5.5	-51.00	aggcaagaugucggcauag	64433	-	0	>NODE_205582_length_2979_cov_7.960725_26160
Par-227	Pae-miR-316	gcacacagaagcuacaguucucgauaaauuugucuuuuuccgcuuugcugcugauccuuguagaaugucaacagcaaagcgaaaaggucuccuuuaugagaacaugcuucg	70.6	-41.20	ugucuuuuuccgcuuugcugcug	46	acagcaaagcgaaaaggucucc	84	>NODE_490220_length_5217_cov_8.499330_42873
Par-044	Pae-miR-317	aguugcagcuagucuccgggcuaucgaacgaccgggugccacgcugugcucucucgggauaaaagaugagugaacacagcugguggguaucucaguuguucccuaguuc	3788.2	-40.80	cgggugccacgcugugcucucu	2	ugaacacagcugguggguaucuca	6218	>NODE_89650_length_2139_cov_6.661057_14657
Par-125	Pae-miR-3286	aacgacacggcgggacgaagcccaaaacaacuauuggucacugcacaggacuaguaaacgugauauacucuugugcguguuccaauaguuauugugggugcuucauaaucga	168.7	-37.40	aacuauuggucacugcacagga	24	uugugcguguuccaauaguuau	299	>NODE_318834_length_20562_cov_8.469410_34248
Par-141	Pae-miR-33	ugaaguugacuguugcgggagugcauuguaguugcauugcacuuuuucacguauauugugcaauaucacuacacggcaauucucgcggcagucgcucucaacacgcuuuc	134	-44.46	gugcauuguaguugcauugca	242	caauaucacuacacggcaauuc	12	>NODE_100027_length_3353_cov_8.405010_15766
Par-344	Pae-miR-3338	gaagaaguucaaacaauguuggacguuucacagcaaacacaguaacgugcaaugcuaaaguucaucaugcauguacuuacuuuguuuguucugaaaugccucaacuagcaac	0.8	-33.12	-	0	auguacuuacuuuguuuguucu	21	>NODE_511388_length_1295_cov_8.365251_43480
Par-021	Pae-miR-34	gcuguacaggacguccgcacuggcagugugguuagcugguuguuuagugcaccacaacagccaguaacggcacugccccugcguacuccuuugauccacugaagaacag	5.4	-46.50	uggcagugugguuagcugguu	13357	cagccaguaacggcacugccccu	13	>NODE_443818_length_7984_cov_9.355335_40764
Par-076	Pae-miR-375	ugcguuuugugacgaggacgacccgagcggaugagcaaacuguauauucagcuaguuuguucgccccggcucgagucguuguuggcacagaguaucagcaaucaucacaua	629	-44.20	acccgagcggaugagcaaacu	916	uuuguucgccccggcucgagucg	301	>NODE_208985_length_6667_cov_8.727613_26475
Par-122	Pae-miR-6094	gauacacuaucaucagcgguggucugggguaccuaauagcagccucucuacccuuugccgggcugagugguauucgagaccucugcugauccuggacccuaauguuuuu	260.9	-43.70	aucagcgguggucugggguac	5	uauucgagaccucugcugauc	491	>NODE_151723_length_2822_cov_6.982991_21021
Par-097	Pae-miR-6307	aagucagaagaaaaacagagcgcgcguggggcgcgggaaaaguucgcauuuugacuugguuuggagggagucaagcucgaacuuuuccugcgucuucgcacgcucacaauc	328	-53.50	gcgggaaaaguucgcauuuugac	94	caagcucgaacuuuuccugcgu	530	>NODE_592132_length_4234_cov_9.070147_45471
Par-158	Pae-miR-7	cucggcgaccgcgcguuguuuggaagacuagugauuuuguuguuugauuaacuauaacaaagaaucacuaaucugccuacaaagcgucggugcacucuucauagaagac	203.4	-37.50	uggaagacuagugauuuuguuguu	385	caaagaaucacuaaucugccu	6	>NODE_222845_length_10399_cov_8.619098_27870
Par-084	Pae-miR-745	acaaugaagcgaagcagacagaacucguguuaguugcggcucaucguaaggcaguuugcuuguaguucgccagcugccuagcgaagggcaacaaguuacagaguuguucgacg	997.9	-39.60	cggcucaucguaaggcaguuugcu	951	cagcugccuagcgaagggcaaca	932	>NODE_226292_length_8577_cov_8.808441_28180
Par-069	Pae-miR-750	gcuccugguacgggauagguuucacgucugaguuggacaggggaucuugacaguuugauuauuauugcugccagaucuaucuuuccagcucacgcguggaaccuccaucaac	996.2	-47.60	aguuggacaggggaucuuga	8	ccagaucuaucuuuccagcuca	1937	>NODE_146393_length_5872_cov_8.470367_20520
Par-100	Pae-miR-8	acacguuucacucaaacaguuagaacguuaccuggcagaaaugauuuuuuaaaauacucccauccugcucgguaacuuucuuuauuguaugaaugacuuucucaauaagaau	332.4	-26.97	uagaacguuaccuggcagaaaug	8	uccugcucgguaacuuucuuuau	626	>NODE_637275_length_403_cov_74.846153_46315
Par-135	Pae-miR-87	aaacaggagagugaagaaguucgguacacugaugggccugaaucguugcucuuaccuguguuuaaauuaggugagcaaacuuucagguguguuagugccgacugugcgacuu	180.7	-41.20	gggccugaaucguugcucuuaccu	72	gugagcaaacuuucaggugugu	274	>NODE_175325_length_1216_cov_6.162006_23264
Par-034	Pae-miR-9	gaagaaagacagguuauuuaucuuugguuaucuagcuguaugaguacucugacaucauaaagcuagguuaccggaguuaagggccgucuguuaucgacguuucgaagaugug	4865.8	-37.40	ucuuugguuaucuagcuguauga	7114	auaaagcuagguuaccggaguua	2420	>NODE_158152_length_6584_cov_7.935146_21718
Par-032	Pae-miR-92a	gcggacgccuuuguucucgggggcccgcguggcuggguugugacuggcgcuauauucgguuugcgagcgauauugcaccagucccggccuauccaaacgggcccggccuu	3180	-52.90	ggguugugacuggcgcuauauu	1245	uauugcaccagucccggccuau	4975	>NODE_44822_length_4691_cov_8.416116_8903
Par-071	Pae-miR-92b	ucacugcuauggccacgcauagggcgugauucgguguaaccguuguuaaaauaucaaauugcaccaaucccggccugcaugcggccguugcuugugagaguguugccuuugacuc	1498.1	-40.70	agggcgugauucgguguaaccguu	135	aauugcaccaaucccggccugc	1505	>NODE_44822_length_4691_cov_8.416116_8906
Par-208	Pae-miR-927	aguagaagcgguguuuuuguuuuagaauuccuacgcuuuaccguugccaaguuuuugggcaaagcguuuggauucuaaaacgaaauauccagcaaaucuacauugaggcaag	89.8	-39.00	uuuagaauuccuacgcuuuacc	71	caaagcguuuggauucuaa	96	>NODE_266184_length_6247_cov_6.530815_31043
Par-616	Pae-miR-929	acaaaaagcucuuggaugucaaauugacucuaguagggaguccguuuacacgcgacucccuaaucgagucagguugacuuacuugaugccuauucuacuuuaagaagaga	4.7	-40.30	aaauugacucuaguagggag	7	cucccuaaucgagucagguuga	4	>NODE_134881_length_3247_cov_8.306437_19410
Par-253	Pae-miR-932	acacaaaggguucugaggccucaauuccguagugcauugcagugcauauagcccccugcaagcagugcggaagugaggcugaggucuccuuaggcaacgaaaccgggcugc	5.4	-48.80	ucaauuccguagugcauugca	79	ugcaagcagugcggaagugagg	47	>NODE_293200_length_5487_cov_8.108803_32779
Par-154	Pae-miR-965	agcuaggacacagcaaaaugcuauauaacugaacgggagaaguuguaacgcucuauguacuauauaaucauaagcguauagcuuuuccccuuaguuguguaugcuaucgcc	195.9	-39.50	cgggagaaguuguaacgcucuaug	163	uaagcguauagcuuuuccccuu	189	>NODE_209060_length_6801_cov_9.389502_26477
Par-023	Pae-miR-970	agaagagacagcuagaguugcuagguagagccuugcgugugaucuuauugguaguguaguagucaaucuaucauaagacacacgcggcucuuccuggcucacucguaacac	4260.1	-47.32	agccuugcgugugaucuuauuggu	4	ucauaagacacacgcggcucu	8315	>NODE_350970_length_9734_cov_8.357818_36278
Par-412	Pae-miR-971	aagaaacaguauucucaguccacuauaaguaugaacgccaagcauauuguacaaaguuuggugugucuaccuuacagugaauugcguauucugucucaucaacugaagaaagaa	9.6	-32.40	cacuauaaguaugaacgccaagc	17	uuggugguucuaccuuacag	1	>NODE_679445_length_22500_cov_8.827023_46863
Par-501	Pae-miR-981	gagaagauaaaugaagaagacaccgcacauaauucggguuucgagacgaugaaaccguaauuauaauugguucguugucgacgaaaccugcaaaaugugcggcaaacuccaa	13.4	-40.00	cggguuucgagacgaugaaacc	11	uucguugucgacgaaaccugca	7	>NODE_162079_length_3164_cov_6.820164_22063
Par-230	Pae-miR-988	aauagaagacuuuauaauugcaauugcccggagugugcguuguggcaaaggagaucaagguaguaccauccccuuguuacuaaccucacuuugguugauuguacauucagc	46.4	-33.20	gugugcguuguggcaaaggagau	2	ccccuuguuacuaaccucacuu	82	>NODE_150616_length_15537_cov_8.267169_20884
Par-002	Pae-miR-989	gacgaagagagaagaaguccaggguugaaauggccauuaccucacagucacguggucuuguaguacaacgugugaugugacguaguggaagucucgaccugggcccucuac	274515.7	-43.60	gccauuaccucacagucacgu	120	gugugaugugacguaguggaag	538321	>NODE_101997_length_1146_cov_7.147470_15966
Par-019	Pae-miR-993	aaauuggcacauucgugaucuacccuguagauccgggcuuuuguagcuuaguuuacaucagaagcucgucucuacagguaucucacggcuggaagaccucgcuacuaugauc	19061.4	-38.26	uacccuguagauccgggcuuuu	30591	gaagcucgucucuacagguaucu	6706	>NODE_354880_length_5608_cov_8.347717_36589
Par-093	Pae-miR-998	guaggugugggagcgggugucggccgggggcggccccgagcuacaucccguggggcuccagugccgcguguagcaccaugggauucagcucagggcugcucacuaacugcaa	714.1	-56.90	agcuacaucccguggggcucc	64	uagcaccaugggauucagcuca	1325	>NODE_268348_length_2785_cov_8.817953_31234
Par-096	Pae-miR-iab-4	gaaaaugcgucgccuucuguacguauacugaauguauccugaguguccgauucuuuccgguauaccuucaguauacguaacagaagaugacacuagccaucgcucgaagaca	365.7	-39.70	acguauacugaauguauccuga	566	cgguauaccuucaguauacgua	140	>NODE_121550_length_1406_cov_8.203414_17983
Par-360	Pae-miR-iab-8	auggcuagugucaucuucuguuacguauacugaagguauaccggaaagaaucggacacucaggauacauucaguauacguacagaaggcgacgcauuuucuucucuucucuuc	5.1	-41.62	uuacguauacugaagguauaccg	23	aggauacauucaguauacguac	7	>NODE_121550_length_1406_cov_8.203414_17982
Par-471	Newly described	uucguguaugaagaaaugguuuggucuguaaucagcuuucgcaauuugccgcacccguuaguaaucugggugggggcaaauugcgaaagccgauuauuugacuuucuagcaac	18.5	-51.70	gcuuucgcaauuugccgcacc	4	gugggggcaaauugcgaaagccg	23	>NODE_134607_length_5904_cov_7.965278_19380
Par-110	Newly described	uaggagccucauugcagugcaaucuaaauccgacagcugugacucucacacuuaauauguguuauauuuacuaggugugagggucacagcaagucguauuuaugaaauc	338.8	-42.10	gcugugacucucacacuuaaua	1	cuaggugugagggucacagcaa	662	>NODE_103998_length_12186_cov_7.991220_16175

Novel miRNAs identified in *Cameraria ohridella*

Working ID	Precursor sequence	mirDeep score	MFE	Putative -5p	5' count	Putative -3p	3' count	Maps to
Cam-041	gaagaaaagaagauauaaaugcuuaauacaugggcugugacucuaacaucuaauauuuuuacaaucacuacuagguguuagggucacagcgaaaguguuaagccagucauca	5722	-41.71	gcugugacucuaacaucuaaua	569	cuagguguuagggucacagcga	10652	>NODE_487546_length_5505_cov_8.651953_22948
Cam-068	gcugaagaaacguucuagaaaguguuuauucauaggcugugaccuuaacaucuaauauuuuaaaugauuacuagguguuagggucagagcgaaugcgugaaauucaucaa	1004.5	-34.50	gcugugaccuuaacaucuaaua	2	cuagguguuagggucagagcga	1968	>NODE_411029_length_1095_cov_7.494977_19667
Cam-078	aagaaggagcaaagagauaaugucauagaaagcaaguacucauucuccgagguugauaagucuguagcuaucacugggagcaaugaauacuaugcuuuuaauga	757.1	-30.30	aguacucauucuccgagguugaua	1	ucacugggagcaaugaauacuaug	1478	>NODE_436495_length_218_cov_10.724771_20813
Cam-090	accuggcuucugcaagaccuaacaacagcggcugaugaaagcucgccguuucaacuauaccuugagucauaguagagucgccgagcuuucauuggugguucguacaaac	734.2	-37.80	aaagcucgccguuucaacuau	364	aguagagucgccgagcuuuca	1072	>NODE_231563_length_2616_cov_6.874236_11889
Cam-091	auugcugacaagcugcacgaaagucgacuucgcugguacggccauguguugagugagagucucagucucaacacauagccguaccagcgaagucgacuuuaccgaacgucuc	875.4	-65.90	gcugguacggccauguguuga	996	caacacauagccguaccagcga	711	>NODE_156108_length_4031_cov_11.506574_8234
Cam-116	uuucagauuaaugucaugggccaugcuuuccaacugaagauugauuugccauggauggaugcuaucaaaggucugcaacggcccaacaaagugguguggcag	-5.9	-24.10	caacugaagauugauuug	1	gaugcuaucaaaggucugcaacgg	233	>NODE_646933_length_55_cov_14.527273_28098
Cam-155	cuauuguuagcuuggcacaguaggaaugugagcgguuuucguuuucuacugaauaucguucgaucccugguucgaauccacaacuuuuuucuuuucuuuuuuauacuuaguacu	239.7	-19.40	caguaggaaugugagcgguuuucg	166	gaauaucguucgaucccugg	101	>NODE_436171_length_1420_cov_5.359155_20790
Cam-169	gaagaaaauaugaaugaagaaaagcuauuggugcacuuauaugaguuuguacccggguuugugacgcccauaaauucggacacuaacucauguauaggcacuauuccucgacu	52.4	-36.01	augaguuuguacccggguuugu	10	uaaauucggacacuaacucaugu	85	>NODE_922570_length_2630_cov_7.145627_35518
Cam-170	uccuugguuuuuagaauacucguaaugaugauaucccauuaacagacuagagucucugaacauugugaaugucaacguuaaucugagguuuaaaacuuauuuuagcuacaagcu	-11.3	-18.70	cguaaugaugauaucccauuaaca	72	aaugucaacguuaaucugagg	28	>NODE_424858_length_3874_cov_13.964894_20296
Cam-172	agauauccgguagagaaugaaugucacgccugugcgcgucggguaagccaucucguggauggggggcaggagagggcuuacccgacgcguggaagucaucauuagaaaacggc	33.8	-52.20	ugcgcgucggguaagccaucu	50	gagggcuuacccgacgcgugga	7	>NODE_1263699_length_660_cov_13.386364_41529
Cam-189	uauguccagcaguggacuucuaaaggcugcaaauuaggaugaucaucaucgucaucauaauuugcagccuuuagaaguccacuugcuuaaauuguauuccuaaaacggaacg	38.6	-57.60	uaaaggcugcaaauuaggauga	65	caucauaauuugcagccuuuag	1	>NODE_156314_length_5775_cov_6.603809_8250
Cam-259	uaccgaaaggaccauugcucuguacgccaguuacugccgacuccauugaucugucauggcauccgccaacggaugacgguggguagugaaauuacuggauacguagucga	4.6	-35.00	uccauugaucugucauggca	2	acggaugacgguggguagugaa	105	>NODE_108002_length_3417_cov_15.207199_5766
Cam-318	cgagccaggcuggcccgacacuauaacgaaggaccaucuuaaucucguacaggcugauaucccggacgucagccuguacaagauuaggauggcucguaagcaacuuaaaaau	9.2	-48.00	aucuuaaucucguacaggcuga	2	agccuguacaagauuaggaugg	7	>NODE_338121_length_1838_cov_9.035909_16496
Cam-346	aaacaacuaaguuaaaagauuugcaacagcuuggcugaucucgagcuaucaucuccugaucagaaagagcauuguugcaaaauaaaagugcccuuucuagacaaggcugcauuc	9.4	-31.60	uugcaacagcuuggcugaucucga	10	cugaucagaaagagcauuguug	1	>NODE_387820_length_400_cov_11.637500_18622
Cam-421	aaauaacuuacuacacgacccaaacaauuaaacgaucggcuaucaaacauuuuguuucuagaaaaucgucuaauuguuuuagucgaguaguaccggcucguauuuaaaauau	5.4	-29.30	caaacaauuaaacgaucggcua	4	agaaaaucgucuaauuguu	1	>NODE_837760_length_2267_cov_7.281870_33893
Cam-432	aucagcugaucaaacgccgcggcaaguugggucuggucggcgugaacaagacggcgagcgaggcgcggcgauggcucggcgagcauaugggcaaagagcagcaggucggcgc	3.4	-45.90	ggcaaguugggucuggucggcg	7	gcgaggcgcggcgauggcucgg	1	>NODE_380314_length_1077_cov_9.726091_18305
Cam-434	ucuccgauuaaaauuaucuugccgcgcugggauuugucgguguucuucacuugaccaaucacagcgcaguucagcuuauuuuaaucauagauuaagaugcuucucuuuuaggaa	9.1	-32.80	gccgcgcugggauuugucgguguu	10	acuugaccaaucacagcgcaguuc	1	>NODE_789019_length_4565_cov_6.536911_32366
Cam-442	auauacgagcuauaccuaaaggccgauuuacauuaucuuaguguuuagaagaguguuuuaguacaacuugaaggucacguagaacggcacuaaagcuaguagcguuuaca	-3.2	-22.80	aggccgauuuacauuaucuu	11	aaggucacguagaacggcac	1	>NODE_877533_length_193_cov_7.341969_34678
Cam-447	ugcuucgaaaaaggccgugaagcuuuggucacacagaacgcgugcgcaagacaugagcgggucaugagcggcguucugugugcccaaggcguuaagccuaaagguaucauua	5.6	-48.90	agcuuuggucacacagaacgcg	4	gcguucugugugcccaaggcg	3	>NODE_242894_length_6203_cov_9.433661_12360
Cam-464	uguuacaggacuaugaccaauucgacauggccaaugaaguggacacuuuagaacaauuucuuggccuagagguaccgccggauucgucacaaucggaaaauggacagguaau	-1.8	-26.60	auggccaaugaaguggaca	2	gguaccgccggauucgucacaa	5	>NODE_238209_length_613_cov_15.869494_12132
Cam-498	cuuggaaaagaucuaagauaguauaaguuaacgaaauauaauuugagcggauucguaguguagcugacacuacgaauuggcucaaauuauauuacgucaacuguagcauuuua	4.6	-49.10	auauaauuugagcggauucgu	1	uacgaauuggcucaaauuauauu	6	>NODE_433978_length_1736_cov_4.421659_20703
Cam-518	uaauagccaguuacuuaccuuacggacaacggguaguuacuuucauacauuuuugacaaucugucaaaacuguaugaaaguaacggcccguuauccguagguuaaguuac	2.1	-57.70	uacggacaacggguaguuac	13	guaacggcccguuauccgua	1	>NODE_694510_length_3037_cov_8.434969_29530
Cam-545	ccuugcuuauauaaguggacaggcugcuguauacuuccugguacugaucggugugcucgugcuaugcuugcuagaugccaucagggagaugcagaaguacucuaauauc	2.1	-29.60	aggcugcuguauacuuccu	4	gggagaugcagaaguacucuaaua	2	>NODE_179351_length_4334_cov_8.357637_9445

Novel miRNAs identified in *Pararge aegeria*

Working ID	Putative primary sequence	mirDeep sco	MFE	Putative -5p	5' count	Putative -3p	3' count	Maps to
Par-013	gcugcuagcaagauuuuaucacuguguacgcgggggcauccauaaaaucaacuauuaauauguguauuuagcugauuuuauggcugucaccggguauauuacagugga	5	-39.60	ggggcauccauaaaaucaacua	101	gcugauuuuauggcugucac	42682	>NODE_470887_length_6655_cov_7.711345_42043
Par-017	augagaagaugagaaguuguauacggagagaaggagugcauguucguagacaaccauguuuguuggcuggcugacuacgagcaugcucucaguuucuccguaaccccacuuc	21437.7	-51.00	gagugcauguucguagacaacc	867	cugacuacgagcaugcucucag	41120	>NODE_88760_length_3699_cov_8.662341_14562
Par-058	cgacgcaaaaguugccugcggugaguacgaacuaauagagguucauaaucacucggaugggaacacucgaucaauaacaacuuuuauuauuucguccgcagagaccgucaca	1220.8	-35.53	gugaguacgaacuaauagagguu	2350	cuuuuauuauuucguccgcag	44	>NODE_210299_length_3464_cov_7.681870_26594
Par-080	ggcuaaaagacuauuaagugaaaucgccugauugaacuaggcgaucuuucuuaauggucauuuagcuugcggucuc	414.2	-32.00	agacuauuaagugaaaucgccu	803	gcgaucuuucuuaauggucauu	7	>NODE_131085_length_1379_cov_5.477158_18955
Par-094	acuguauacagguuguacuggcgauagugcacuaucaguaggcugugguucugcuaaaauagcccugggauugauagugacuagcaccagauguuuguguauaggcugaccaguca	848.1	-41.10	uuguacuggcgauagugca	1	ugauagugacuagcaccagauguu	99	>NODE_655427_length_690_cov_38.952175_46596
Par-099	cccugggaugguuacgucccaccgguaaacgcagcguugcuaccaacgcgcaacacugcguuuaccggugcggcgucgccauaccaccauacgccauaccccugccgagcugg	332.1	-55.50	ccaccgguaaacgcagcguugc	601	aacacugcguuuaccggugcgg	39	>NODE_187039_length_3875_cov_6.640000_24273
Par-104	aagaaucgccggaacagaggcgauccuugagaugggacauguagccuucuaucacacauuuacaugucccauaccaagaaucuuuuagaacagaggcgauccuugagauug	470.5	-36.52	cgauccuugagaugggacaugu	654	caugucccauaccaagaaucuu	251	>NODE_103890_length_6601_cov_6.564157_16158
Par-111	auuggguucuuugugucuauuauuuuuguacucauguaguuuguauauuucugauuacgugagcacaaaaacuauaggcgaggaccaaccaaacaaaccaaccaaacuaugg	4.6	-34.60	uauuuuuguacucauguaguuugu	286	uuacgugagcacaaaaacuaua	5	>NODE_556918_length_20507_cov_8.602428_44830
Par-117	aagcacgaggagacucaggcugucgaaucuuggucccguccucggccacuaggauucgacaaccagggacuucccguugcauuggauggag	347.6	-40.90	aggcugucgaaucuuggucc	606	ccacuaggauucgacaaccagg	67	>NODE_365730_length_16177_cov_8.763059_37394
Par-118	gggguagguggcgcagggcuucguuggugacucggucggagccgggggcuuuugcgcaguuuaguucggcgaaucauccuguggacuuguccuggggaga	1	-36.40	ucguuggugacucggucggagc	80	ugcgcaguuuaguucggcga	2	>NODE_166697_length_457_cov_3.411379_22501
Par-129	caaugguaucugcugagaucuaauugggaauuucuggggagaaaucccuaauuaaaucucggcggauauaaaauguuaca	5.1	-35.50	ugagaucuaauugggaauuucu	153	aucccuaauuaaaucucggcgg	599	>NODE_175544_length_1356_cov_5.526549_23271
Par-145	agaacauuguugaauaauaucuuacaagauauuacucuacucuuucagacacaguauuauuaugaaccugugugugaauaaguagggaauucuuuguaugugauauguaugug	194	-31.80	uauuacucuacucuuucagacaca	87	ugugugaauaaguagggaauucu	286	>NODE_83100_length_543_cov_5.130755_13891
Par-146	ucaagaucuuccaugcguuucauauauuuuaagguggacuucaucaaaucuaucaacgacuuacguugcgcugauagauuugaugaaguccuccucaaaguaucuaucuu	188.4	-42.60	acuucaucaaaucuaucaacg	61	cugauagauuugaugaaguccu	272	>NODE_338451_length_5175_cov_8.079614_35437
Par-152	accaggcuguuucgauccggacagucuacccggacaggccgacuugucuaugucgcccuauccggauagacgguccggauuguuaccgcaauucugcauauagaagaaagaa	191.3	-52.50	acagucuacccggacaggccga	326	cccuauccggauagacggucc	46	>NODE_130843_length_2132_cov_8.431050_18941
Par-160	aauguaugcggugcauacacacccggacauugguaugguaaacagucguuggagauuaccaucccguugucuguguguguauaaaaauuauugcagcuauggaaaaugugg	126.7	-35.10	acccggacauugguaugguaa	216	uaccaucccguugucugugugu	32	>NODE_315071_length_2696_cov_5.650223_33970
Par-168	uuuuguagggaguuccaaacagaacguucuugugccgcuugaguacuggacucaagcggcacaagaacgugcuguuugggcccccagcgacucguuugaagucaucugcgu	193.2	-64.70	agaacguucuugugccgcuug	232	aagcggcacaagaacgug	133	>NODE_108754_length_3319_cov_7.269057_16675
Par-181	gaaucuguaaacugaacuaaauggacaggccuaaaaauagcgcuguccuuuucccccagggaacuuguaaaagauagugcuacuuuuagacuugugagacauuuuaaaauua	72.6	-29.10	ccuaaaaauagcgcuguccuuu	30	aagauagugcuacuuuuagacu	114	>NODE_552053_length_7825_cov_9.408946_44650
Par-189	gaacaggaaauugggucaaagauauaacaugguguuccuguucuucuccuaccgcccaaaguacuucaugggcgguagaagaagagcgagaagaccauuccgaaggccaga	45.3	-46.71	cuguucuucuccuaccgccca	4	ggcgguagaagaagagcgaga	83	>NODE_299995_length_7209_cov_9.668747_33164
Par-211	cuuguugccugccgaguuccacuauuagaaugaaauaguacuaacuauugacauucauuuuaauagcgguacuuguugggcaacuauucaacau	4.8	-37.00	uccacuauuagaaugaaauaguac	105	ucauuuuaauagcgguacuug	9	>NODE_220488_length_52_cov_10.230769_27641
Par-212	cuuuuugccugccgaguuccacuauuagaaugaaauaguacuuacuauugacauucauucuaauagcgguacuuguugagcaacgauuc	3.8	-29.90	uccacuauuagaaugaaauaguac	106	uucauucuaauagcgguacuug	19	>NODE_79784_length_52_cov_45.365383_13482
Par-220	uuuacuuguggauacggcggauauuuggaguucaaagauuuacagcuuucaacuuugaauauccguuguacccuaag	49.1	-32.80	cggcggauauuuggaguucaa	13	ucaacuuugaauauccguugua	82	>NODE_208047_length_5992_cov_6.435414_26405
Par-222	guauacaaauucuaaaauaagucgacauaucuaaaaguagugccaucauuuucuaaacaaagcaucgugaaaaggauggcauuuuguacuuuaauguccaauuugugguuug	50.5	-27.06	aaguagugccaucauuuu	1	aaaggauggcauuuuguacuuuua	80	>NODE_120689_length_3235_cov_8.975579_17919
Par-240	acccgcauaaugcacaggaguugaguuuaguguggcaaauuucggguuuuauuuuauaaauauuuuuugaauaaaccugaauuuucuacacauaaauucuaacaucaaau	37.5	-29.50	uguggcaaauuucggguuuuauuu	13	auaaaccugaauuuucuacaca	62	>NODE_32494_length_2874_cov_16.130133_6775
Par-247	acaccucucugaguacgacauacgauaaggccuguagacaacggguucagagucuucgagcucaauugucauuucaguagcauucguauauccuaaaucuuuaagacuguucga	-6.7	-22.90	uacgauaaggccuguagacaacgg	42	ucauuucaguagcauucguauau	2	>NODE_616280_length_1324_cov_21.438822_45881
Par-259	gaucggcgaaaaucucgcgaucacugcugucagacgucacuuuuccauacacgaaauaaacaagugacguuugacagcagugaucguaagguuuacggaacucuuuauuagu	5.6	-53.51	ucacugcugucagacgucacuu	10	aagugacguuugacagcaguga	67	>NODE_567223_length_8238_cov_8.848750_44964
Par-262	cauuucucggcacuuguguuucuuuuucuaaagaggucaguauaaauuugacaguacuguucuccuuagaaaaaaauagcaccggugccaauauccuacgaucgcaucgucu	31.1	-37.40	ucuuuuucuaaagaggucagua	58	cuguucuccuuagaaaaaaa	1	>NODE_110532_length_6374_cov_8.879981_16909
Par-268	uaaauaaaucagaaagaucauugcuguccgccgcaaaugcuaagcuacuuccagguaucaggguagcuucaugccuggaaguaacuuaacuauugcggugcucuucugauau	28.4	-42.80	gcuaagcuacuuccagguaucagg	1	augccuggaaguaacuuaacua	46	>NODE_175291_length_832_cov_7.888221_23246
Par-269	auugcuguccaccgcaaaugcuaagcuacuuccaggcaucaggguagccucaugccuggaaguaacuuaacuauugcggugcucau	32.2	-44.20	gcuaagcuacuuccaggcauc	46	augccuggaaguaacuuaacua	46	>NODE_8331_length_836_cov_44.717705_1912
Par-280	ggaagauuucgagaguuguuccauuuuucuucuauaaggcugaauuacugccgacagucucgucuggauagaauucggccuuuauaggagaaaagaugagacaaaggucguc	33.5	-42.60	ucuucuauaaggcugaauuacugc	14	gaauucggccuuuauaggagaaa	43	>NODE_293563_length_4157_cov_6.324272_32810
Par-284	uauaaauuacaccauacagannnnnnnnnncuaagguuacugguuggucgacaauauuuauuaugaaaauauugucggaccaaacaguagccuuaguacaagauuaacagg	28.4	-44.10	uacugguuggucgacaauauu	18	uauugucggaccaaacaguagc	36	>NODE_195801_length_7222_cov_8.752007_25163
Par-290	gaaggaucaagagcgagaauggggagaauuuuaacaacuuacuaucuguguuguaucaaauaucuuacugaucucgacagguaucucuugugcaaggag	-5.5	-20.30	aagagcgagaauggggagaa	1	aaauaucuuacugaucucgacaug	32	>NODE_131368_length_809_cov_13.164400_18991
Par-305	cguuaacgacauucccauggguggacuuaguaaucauccggaugacuuauccggaugaucaguaaguccaccgauggggucuugaauucaaacaugcuucaauuuggguuaau	5.5	-50.30	guggacuuaguaaucauccgga	50	uccggaugaucaguaaguccac	1	>NODE_56670_length_730_cov_7.990411_10740
Par-324	gaaagaucgcucaacucccugcguucacgaccucguaaggaaaaaucggguaauuguucccuuacgacguggugaauccggggaagugagcggggaagcuacaggagcguug	30.1	-47.20	gcguucacgaccucguaaggaaa	42	cccuuacgacguggugaauccgg	8	>NODE_210778_length_7172_cov_8.505159_26630
Par-340	aaguggugagcaaguugcgcugggauauuauaggauugucugaaguccggcgaggacucgauaaucuuggaauccggcaacuuguucuacuaccgggauauuucuuacuugug	5.2	-43.60	ugggauauuauaggauugucuga	45	aucuuggaauccggcaacuug	4	>NODE_310986_length_3085_cov_6.844084_33711
Par-341	aaguggugagcaaguugcgcugggauauuauaggauugucugaaguccagcgagaggggaggacucgauaaucuuggaauccggcaacucguucuacuaccgggagggugacc	5.1	-41.10	ugggauauuauaggauugucuga	45	auaaucuuggaauccggca	3	>NODE_284157_length_2198_cov_6.729754_32269
Par-350	cgcagaugacuucaaacgagucgcugggggcccaaacagcacguucuugugccgcuugaguccaguacucaagcggcacaagaacguucuguuuggaacucccuacaa	12.2	-64.30	acguucuugugccgcuugag	2	aagcggcacaagaacguu	19	>NODE_108754_length_3319_cov_7.269057_16673
Par-371	caguggguuuaagggacaaugugugugguggucaauuaccaccaugcacauugucccuuaggccacuucaaacaaguaacaaacuacacugca	16.6	-52.90	uaagggacaaugugugugguggg	22	accaugcacauugucccuuagg	1	>NODE_350391_length_3251_cov_8.677945_36231
Par-377	uaaguugaaguuguuaaaucuuaccaguggguuucggccuuuuuccacgcguaguguguauuuuaauaacuaaccguagacgaauuccgaaaucuacgcguguaagacaugc	9	-29.62	uucggccuuuuuccacgcguag	1	uaaccguagacgaauuccgaaa	18	>NODE_626413_length_1576_cov_4.972081_46192
Par-404	caaaagcugacccaucggucuggugcucaaguagcuggcguuuauuuagugcaauaggaaauaacgccaguuucuuagcacaauaccuuuggguucuccacuu	5.1	-38.00	uggugcucaaguagcuggcguu	15	aacgccaguuucuuagcacaau	4	>NODE_41228_length_575_cov_52.373913_8431
Par-405	uuacaaaagacccauuggucuggugcucaaguagcuggcguuuauuuagugcaauaggaaauaacgccaguuucuuagcacaauaccuuuggguucuccacuucgacggcgc	5	-39.00	uggugcucaaguagcuggcguu	15	aacgccaguuucuuagcacaau	4	>NODE_529540_length_2122_cov_4.117814_43982
Par-425	accaaauuuauaggcuauagaccuauauaccuacuuccccgauguacgcauaaguacaucggggaaguagguauauaggucguuauuuuaucccuccc	24.3	-54.90	uauauaccuacuuccccgaugu	1	caucggggaaguagguauauag	37	>NODE_275818_length_12912_cov_7.746593_31802
Par-426	ucuggucuugucuggugggagggauaaaauaacgaccuauauaccuacuuccccgauguacuuaugcguacaucggggaaguagguauauaggucuauagccuauaaauu	23.3	-58.80	uauauaccuacuuccccgaugu	1	caucggggaaguagguauauag	35	>NODE_275818_length_12912_cov_7.746593_31799
Par-523	aagaacaaggauauauuauuaacgccaacaguuuguucaaaaauguugaaagaaguuguuucaacauuuuugaacaaacuguuggcgcuaauaauauauccuuguucuuc	15.5	-71.90	acgccaacaguuuguucaaa	1	ugaacaaacuguuggcgcua	19	>NODE_330412_length_2440_cov_8.093033_34905
Par-536	aauaaauaauggguggcugccauauauggcugacgcggcgccacuguaauaaaauucucaauggggccgcgucagccauuuugucagccauaugauuucaaaagcgcgcgaaa	6.2	-50.70	auauauggcugacgcggcgcca	8	ggggccgcgucagccauuuugu	2	>NODE_60260_length_1121_cov_95.742195_11316
Par-542	agcauagacggguuggggguuaaggcacucuguggaauauuauguacgauaaauauuaauauuucaauguagugccucaacuuccgcucacgucuagucugcaguggcguca	9.7	-37.50	aaggcacucuguggaauauua	10	auauuucaauguagugccucaa	1	>NODE_225066_length_1193_cov_5.608550_28023
Par-543	agcauagacggguugggauuaaggcacucuguggaauauuauguacgauuaauauuaauauuucaguguagugccucaacuuccguucacgucuauucugcagugguguca	11	-35.10	aaggcacucuguggaauauua	10	auauuucaguguagugccucaa	4	>NODE_495496_length_319_cov_35.692791_43038
Par-554	uggcuagcucgaggagagucucggugccgaccgcuaaugcccauuuuuugggcauuagcauucgacaucaagggacucuccacugcuuuuugccugacuggaaggaguucga	11.9	-52.80	ucggugccgaccgcuaaugccc	6	gcauuagcauucgacaucaagg	8	>NODE_365730_length_16177_cov_8.763059_37396
Par-567	uuuucaccugguauaauuacgucuagccguauucucauucugccuuagucauaaaaguagaaugagaauaugguagcauuaauuaauuacc	12	-30.60	cuagccguauucucauucugcc	3	uagaaugagaauaugguagca	12	>NODE_287541_length_370_cov_52.302704_32419
Par-602	agguuaauuaggcuauaaggaucgcagugcuuuuaauguaguuuguacccucccccaaccuaccuagaaguacuguguccuuuauagacuaaccaucaucacuucuacaaucaa	9.3	-34.10	aucgcagugcuuuuaauguaguu	10	cuaccuagaaguacuguguccu	1	>NODE_175291_length_832_cov_7.888221_23247
Par-605	ugcuuuagggccuuaaauauaaauugacauguuuuugugauuuuguucauaaaccaaacgucaaaaccuugucaauuaauauuugagguggccuauauacuaugacggagua	6.3	-29.00	aaauugacauguuuuugugauu	6	cgucaaaaccuugucaauuaau	1	>NODE_232950_length_538_cov_5.061338_28751
Par-614	aagguguuuuaggauuuaauaacacacaguacagacgugaauguacuuucacgucuguacugugguguuauuaaauccuaaaacaaaucccg	0.4	-60.80	aauaacacacaguacagacgu	1	cacgucuguacuguguguuauu	7	>NODE_251833_length_2310_cov_8.620779_30175
Par-634	cucggcuuuucuucguacuugguuggcgggaaaucguucggcgucgacuucccgccaaccaaguacggagaaaagcccac	6	-59.60	ucguacuugguuggcgggaaa	13	cuucccgccaaccaaguacgga	4	>NODE_459370_length_14515_cov_8.248708_41441
Par-658	gaauccaccgcaucugaagaaccaaauaguuacagcuuaaauuuggccucucugaugugguucgauucuu	3.4	-25.80	caccgcaucugaagaaccaaau	5	uuggccucucugauguggucga	1	>NODE_208047_length_5992_cov_6.435414_26404
Par-659	ucuuccuuauaacgauucuacuggcaaucuauguaaagucaacauagauugccagaagaaucguuuuaagggaaauu	9.2	-43.40	aacgauucuacuggcaaucuau	5	agauugccagaagaaucguuuu	11	>NODE_65880_length_7158_cov_9.488544_12008
Par-700	uacacaaauuucaaaacuacuaaauugacagaucuagaaguaacgcuguccuuuucuacaguauacauugugagaaggauagcacuacuuuaagaacugucauuauagugua	4.3	-42.50	agaaguaacgcuguccuuuucu	3	ugagaaggauagcacuacuuua	7	>NODE_300663_length_3875_cov_9.195354_33196
Par-737	uuauguauuuuugaagaguucuguagucggaaacucuuguuuccgacuacagaacucuucaaaaauacagc	6.4	-55.60	gaagaguucuguagucggaaac	2	uuuccgacuacagaacucuuc	1	>NODE_217853_length_17295_cov_8.070599_27364
Par-738	gcauguuggagcuuuuagucauugguacucuacuauuuauuuuaaucuuaaaugguggguucaucaaugcauaaaguucuaacauuaaa	8.5	-30.50	uagucauugguacucuacuauuua	6	aauggugguucaucaaugcauaa	3	>NODE_651474_length_165_cov_11.787879_46550
