# Supplementary material for: A Burst of miRNA Innovation in the Early Evolution of Butterflies and Moths
Source: Mol Biol Evol. 2015 Jan 8;32(5):1161–74. doi: 10.1093/molbev/msv004 (PMC4408404; doi:10.1093/molbev/msv004)
Supplement: Supplementary Data [file supp_msv004_Supplement_S3.pdf]

Alignment and structures of “Par-471” sequences identified by blastn

```
Plutella_xylostella        --------TAATCAGCTTTCGCAATTTGCCGCACCCGTTAGTA-ATCTGGGTGGGGCAAATTGCGAAAGCCGATTATTTGAC-TT--------
Manduca_sexta              ---GCCTATAGCTAGCTTTCGCAATTTGCCGCACCCGTAAGTC-ATCTGGGTGGGGCAAATTGCGAAAGCTGATTATTTGGC-TT--------
Bombyx_mori                ---GCCTATAGCTAGCTTTCGCAATTTGCCGCACCCGTATGTC-ATCTGGGTGGGGCAAATTGCGAAAGCTGATTATCTGGCTTT--------
Danaus_plexippus           TTAGCCTGTAGCTGGCTTTCGCAATTTGCCGCACCCGTGCTTA---TTGGGTGGGGCAAATTGCGAAAGCTGATTATTTGGCT----------
Pararge_aegeria            TTGGTCTGTAATCAGCTTTCGCAATTTGCCGCACCCGTTAGTA-ATCTGGGTGGGGCAAATTGCGAAAGCCGATTATTTGACTTTCTAGCAAC
Heliconius_melpomene       TTAGTCTTTAGTCCGCTTTCGCAATTTGCCTTACCCGTTACGC-ACTTGGGTGGGGCAAATTGCGAAAGCTGATTATTTGACTGACTATCATC
Polygonia_c-album          ------TGTAGTGAGCTTTCGCAATTTGCCGCACCCGTTTCCTTAGCCGGGTGGGGCAAATTGCGAAAGCCTATTATGTGCTTGT---TTATC
```

```
>Plutella_xylostella_"Par-471"
UAGUCAGCUUUCGCAAUUUGCCGCACCCGUAAGUGUAUCUGGGUGGGGCAAUUGCGAAAGCUGAGUAUUUGCUU
(((((.(((((((((((((((.((((((.........)))))).))))))))))))))).)))))............... (-43.40)

>Bombyx_mori_"Par-471"
GCCUAUAGCUAGCUUUCGCAAUUUGCCGCACCCGUAUGUCAUCUGGGUGGGGCAAAUUGCGAAAGCUGAUUAUCUGGCUUU
(((.((((.(((((((((((((((((.((((((........)))))).))))))))))))))))).))))..)))... (-48.30)

>Manduca_sexta_"Par-471"
GCCUAUAGCUAGCUUUCGCAAUUUGCCGCACCCGUAAGUCAUCUGGGUGGGGCAAAUUGCGAAAGCUGAUUAUUUGGCUU
(((.((((.(((((((((((((((((.((((((........)))))).))))))))))))))))).))))..))).. (-48.40)

>Danaus_plexippus_"Par-471"
UUAGCCUGUAGCUGGCUUUCGCAAUUUGCCGCACCCGUGCUUAUUGGGUGGGGCAAAUUGCGAAAGCUGAUUAUUUGGCU
..((((.((((..(((((((((((((((((.((((((.......)))))).)))))))))))))))))..))))..)))) (-50.30)

>Pararge_aegeria_Par-471
UUCGUGUAUGAAGAAAUGGUUUGGUCUGUAAUCAGCUUUCGCAAUUUGCCGCACCCGUUAGUAAUCUGGGUGGGGCAAAUUGCGAAAGCCGAUUAUUUGACUUUCUAGCAAC
............(...(((...((((.((((((.(((((((((((((((.((((((.........)))))).)))))))))))))))).))))))..))))..))).)... (-51.70)

>Heliconius_melpomene_"Par-471"
UCAAGAACUAAUUAGUCUUUAGUCCGCUUUCGCAAUUUGCCUUACCCGUUACGCACUUGGGUGGGGCAAAUUGCGAAAGCUGAUUAUUUGACUGACUAUCAUC
...........((((((..(((((.((((((((((((((((((((((((.........)))))))))))))))))))))))).)))))...))))))........ (-51.70)

>Polygonia_c-album_"Par-471"
UGUAGUGAGCUUUCGCAAUUUGCCGCACCCGUUUCCUUAGCCGGGUGGGGCAAAUUGCGAAAGCCUAUUAUGUGCUUGUUUAUC
.((((((.((((((((((((((((.((((((..........)))))).)))))))))))))))).))))))............. (-44.70)
```
