# Supplementary material for: A Burst of miRNA Innovation in the Early Evolution of Butterflies and Moths
Source: Mol Biol Evol. 2015 Jan 8;32(5):1161–74. doi: 10.1093/molbev/msv004 (PMC4408404; doi:10.1093/molbev/msv004)
Supplement: Supplementary Data [file supp_msv004_Supplement_S4.pdf]

Novel miRNAs in *Plutella xylostella* as identified by Etebari et al (2013)

Precursor	5'	5' sequence	3'	3' sequence	Remarks
tgcggcgccgctatgAACGCTTTGTGAGCTACATTATacattcaactaaatAATGTAGCTCACAAAGCGTTCAagtcggcaccaag	PC-5p-10726_37	AACGCTTTGTGAGCTACATTAT	PC-3p-317_428	AATGTAGCTCACAAAGCGTTCA	
gctggcgggcggtgcggACAGATCGTGATGGCCTTAGActttcgtagatcTAAGGTCATCACGTTTTGTCCctaccgtccagccgaa	PC-5p-44686_10	ACAGATCGTGATGGCCTTAGA	PC-3p-1778_110	TAAGGTCATCACGTTTTGTCC	
cagtgccgccgggatcggcccaaataacaagctaataacatcatccACCCCTAGCGGTCACGAATGGTTctgttatgtggtggttagtacaaCTATTCGTGACCGTTAGGCCTTatccgccttataactatttaaaaagttactgataggtggtcactgactgaaagtgagtctg	PC-5p-2710_86	ACCCCTAGCGGTCACGAATGGTT	PC-3p-369_376	CTATTCGTGACCGTTAGGCCTT	
gaccaagtACGGGTTTGCAAGCCACATATAattacagtatgTATGTGACTTGCAAAGCCAAGCttgtat	PC-5p-22660_19	ACGGGTTTGCAAGCCACATATA	PC-3p-26823_18	TATGTGACTTGCAAAGCCAAGC	
tcgtcgatACTGTGATCTCACCCGCAAGTcggccgtgcacTCGCAGGTGAGATGATAGCATTgaagg	PC-5p-6063_55	ACTGTGATCTCACCCGCAAGT	PC-3p-713_217	TCGCAGGTGAGATGATAGCATT	
aatggaagaatgctgtggtggagcAGAGTCTTGACATGTTCCAACGtgttgtcggtgccgCTGGGGCATGTCAAGACTCGGCtgcatcacggcgatggcctcg	PC-5p-82_1775	AGAGTCTTGACATGTTCCAACG	PC-3p-5943_66	CTGGGGCATGTCAAGACTCGGC	
cattctaaaaggtacAGCAATCGCATGGTCAGTAGACAgtttttgaataatgTCACTGGGTGTGTGATGCCTGTgtctgttagatac	PC-5p-33015_14	AGCAATCGCATGGTCAGTAGACA	PC-3p-362_379	TCACTGGGTGTGTGATGCCTGT	Coh-154
tatgtaattagtggcggAGTAATTTCCAGATAAACGTAcgtctttatatctaCGTTTATTTGGAAACTATCCCggcacaattaccat	PC-5p-52_2942	AGTAATTTCCAGATAAACGTA	PC-3p-11250_36	CGTTTATTTGGAAACTATCCC	
tgccagcgtccAGTGGCAACAGTTGCCAACAGAcattttgaaacctggttttTCTGGCGGCAACTGTTGTGGACaggacgctgtaa	PC-5p-143808_3	AGTGGCAACAGTTGCCAACAGA	PC-3p-29164_15	TCTGGCGGCAACTGTTGTGGAC	
gtcgtccgtctagcgCGGGGCATCTTGTGTCGAAAACTaccagTTTCGACACGAGATGCTCTGTGCtaggcggacctg	PC-5p-69049_7	CGGGGCATCTTGTGTCGAAAACT	PC-3p-38941_12	TTTCGACACGAGATGCTCTGTGT	
caagcggCGGTCTGTTCTCGTCGATactgtgatctcacccgcaagtcggtcgtgcacTCGCAGGTGAGATGATAGCATtgaagggaggagcggcaaccacgcact	PC-5p-189942_3	CGGTCTGTTCTCGTCGAT	PC-3p-26218_18	TCGTAGGTGAGATGATAGCAT	
aatgataaggcatcttCTTCTCTACTGAGTCTGGCAGTGgttttaatttaatattcaCTGACAAACCCAGTAGAGAAATagattacttatttta	PC-5p-579_253	CTTCTCTACTGAGTCTGGCAGTG	PC-3p-166865_3	CTGACAAACCCAGTAGAGAAAT	
gtgtccctatctactatctagcgaaaccactgccaagggaacgggcttggaaaaattagcggggactcagcggccaaagaagaccctgttgagcttGACTCTAGTCTGGCACTGTAAGGAGACATGAGAGGTGtagaataagtgggagacagtgggcttcggtttgctgttcgactgtgaaataccactactttcatcgtttccttacttactcggatgggcggagagcgtg	PC-5p-4356_65	GACTCTAGTCTGGCATTGTAA	PC-3p-2819_83	ATTGTAAGGAGACATGAGAGGTG	
tttctcttctggacGATGAGTGAATTTTTAGTTCACGttttgacaataccgTGACTAGATTTTCACTCATCCTCtatgggagcga	PC-5p-107226_5	GATGAGTGAATTTTTAGTTCACG	PC-3p-30516_16	TGACTAGATTTTCACTCATCCTA	
aaatggccaatttagtgGCCGTTACCTCTGAGCTTGTAtgtccataaattcatcCAAATTCAGAGGTAACGGCACActacattaggctactg	PC-5p-7336_48	GCCGTTACCTCTGAGCTTGTA	PC-3p-63_2387	CAAATTCAGAGGTAACGGCACA	
ccgGCGAGAAGCCGTATCGTTGCCAgttgagtgcgtcACAGCGATACGGCTTTTCGCTTg	PC-5p-49859_9	GCGAGAAGCCGTATCGTTGCGA	PC-3p-43425_11	ACAGCGATACGGCTTTTCGCTT	
atggcaatggccgccttgtcaGGGGGTTTTTGGATACGCAGAAtgttttgtcatacatcCAGCATATCCAAAAACTCATCTGataatttggcctctgcgtc	PC-5p-14178_31	GGGGGTTTTTGGATACGCAGAA	PC-3p-505_284	CAGCATATCCAAAAACTCATCTG	
taatctgacattagtcaGTGACATAGCTGGGATAAGTTagtgtgaaatgaaaCCTATCTCAGCTATGTCACTAtctcgtgtcaggaat	PC-5p-1600_118	GTGACATAGCTGGGATAAGTT	PC-3p-61_2483	CCTATCTCAGCTATGTCACTA	
ccgagttgctgTAAGGAACTTAAATCGAATGTCgtcatgtactgtcaaagtaaggctttgaaggttgaCATTCGGTTTAAGTTCTTTTCTgcaactagc	PC-5p-52114_9	TAAGGAACTTAAATCGAATGTC	PC-3p-55819_8	CATTCGGTTTAAGTTCTTTTCT	
cttagccccttctcccacTATACGACTCTCTGGCGATGCCacagcgactacgactggCTTCGCCAGAGAGTCGCATAGTgggagatggggtttaa	PC-5p-4606_65	TATACGACTCTCTGGCGATGCC	PC-3p-10703_37	CTTCGCCAGAGAGTCGCATAGT	
gtgcccaaTGACGGCATCGGCAGCCTAATcatgtgctaaaatcctGAAGTCTGCCGATGCCGTCAGTgggacc	PC-5p-66168_7	TGACGGCATCGGCAGCCTAGT	PC-3p-11299_35	GAAGTCTGCCGATGCCGTCAGT	
gtgtcgcacggacTGACTGACACCAACTATCTACAgctgacacacctgTAGATAGTTGGTGTCAGTCAGTccgtgcgagca	PC-5p-160069_3	TGACTGACACCAACTATCTACA	PC-3p-94124_5	TAGATAGTTGGTGTCAGTCAGT	
