# Supplementary material for: A Burst of miRNA Innovation in the Early Evolution of Butterflies and Moths
Source: Mol Biol Evol. 2015 Jan 8;32(5):1161–74. doi: 10.1093/molbev/msv004 (PMC4408404; doi:10.1093/molbev/msv004)
Supplement: Supplementary Data [file supp_msv004_Supplement_S5.pdf]

Alignment of miR-2768 sequences

```
C. ohridella  ---------------------GUCCGUUGCAAUAUUUUGACCAAUUUUAAAUUUCGAAUGGACCAAGUUCCAAAUUCGAAUCCUGUGUGAAUUGGUUAAGAUAUUGCAUCGUCUGCCUGUAACGGACCAAU--------
B. mori       --------------GUCGGGCGACCGGUGAAAUAUUUUGACCAAUUUUAUAUAUCGGUCUGAUCG-----------UUGACUCAAGGUGAAUUGGUUAAGAUAUUGCAUCGUUUGCUCGAUC-----------------
D. plexippus  CGGCCGCCGUGGCCUUGAGGCGAUCGGUGUCAUAUUUUGACCAAUUUUAAAAACUUA---------------------AUUAUAUUGUGAAUUGGUUAAGAUAUUGCAUCGUCCGCUUCAAGGGAGUACCAGCGUGGUG
P. aegeria    --------------GUGAGGCGGUCGGUGCAAUAUUUUGACCAAUUUUAUAAUAAAC--------------------UAGUAGUGUAUGAAUUGGUUAAGAUAUUGCAUCGUCCGCUUUAU------------------
P. c-album    --------------GUGAGGCGAUCGGUGCAAUAUUUUGACCAAUUUUAAAAUAAGA--------------------UAGUUUUGUAUGAAUUGGUUAAGAUAUUGCAUCGUCCGCU----------------------
H._melpomene  -----GGAGUACCCGUGAGGCGGUCGGUGCAAUAUUUUGACCAAUUUUAAAAUGACA--------------------CAGUAAUGUAUGAAUUGGUUAAGAUAUUGCAUCGUCCGCUUGACGGGAAUGCC---------
M. sexta      -----------------AGGCGACUGGUGAAAUAUUUUGACCAAUUUUAUGCGUAGUAAUGUUCGCGUUUGAUUUAGAACGUGAGUGUGAAUUGGUUAAGAUAUUGCAUCGUUCGCUUG--------------------
```

Insect Ci peptide aligments

```
CLUSTAL O(1.2.0) multiple sequence alignment


Manduca sexta                   ------------------------------------------------------------
Drosophila melanogaster         ----------MDAYALPTYFPLAYSELQFLASRRAAAVAAAATVLPGSPCINQHHPTDVS
Tribolium castaneum             MPEKEADFQAAMSAGLPLQFPSAFAAFHAP--------------LPV---DQ---RTHEG


Manduca sexta                   ------------------------------------------------------------
Drosophila melanogaster         SSVTVPSIIPTGGTSDSIKTSIQPQICNENTLLGNAGHQHNHQPQHVH---NINVTGQPH
Tribolium castaneum             RYVWDPRMQPPGAP------------------------FHHPTPPGVSGSPGSGLQLLGR


Manduca sexta                   ------------------------------------------------------GLEYLS
Drosophila melanogaster         DFHPAYRIPGYMEQLYSLQRTNSASSFHDPYVNCASAFHLAGLGLGSADFLGSRGLSSLG
Tribolium castaneum             ELHPAYRLPPHMEYLYSLQHSTAN--------------SIHGLGLS-PEYLSARGLSDLH
                                                                                      **. * 

Manduca sexta                   AARSLHPELHAGSTLASQDFQLSLDGKKHSSGSRIASPTRLRLSGGAIGASANRKRAVSW
Drosophila melanogaster         ELHNAAVAAAAAGSLASTDFHFSVDGNRRLGS--------PRPPGGSIRASISRKRALSS
Tribolium castaneum             ----------PSSALASSEFPFSIDGSR-LNS--------PRP--GSIR--QSRKRALSS
                                           ..:*** :* :*:**.:   .         *   *:*    .****:* 

Manduca sexta                   SPYSAESLDLAAVIRASPASLAVRA----PSAASTGSYGHLSAGAISPALSLSHASLAQQ
Drosophila melanogaster         SPYS-DSFDINSMIRFSPNSLATIMNGSRGSSAASGSYGHISATALNPMSHVHS-TRLQQ
Tribolium castaneum             SPYS-DSFDINS-IRYSPNSLVLV-NGSR-SSSASGSYGHLSAGAISPAFNMHPELHLQQ
                                **** :*:*: : ** ** **.        *::::*****:** *:.*   :      **

Manduca sexta                   LLARGGVVGSSGVLAGGVLLDPAHQQAAAAA----AHHAAHAHLVAGI-HRHISSPTQLL
Drosophila melanogaster         IQAHL-LRASAGL-----L-NPMTPQQVAASGFSIGHMPTSASLRVNDVHPNLSDSHIQI
Tribolium castaneum             LQAHL-MRSA-GS-----L-LPLPPATPTHSMYSLGHHPLHVSSPHNISKTD--------
                                : *:  : .: *      *  *      : :    .*    .       : .        

Manduca sexta                   MGAP-----VDVRPG--L---GLDGT-----PPQHL--QQPPQQSEVTSIMEADRRKSPQ
Drosophila melanogaster         TTSPTVTKDVSQVPAAAFSLKNLDDAREKKGPFKDVVPEQPSSTSGGVAQVEADSASSQL
Tribolium castaneum             ---------------------ALN--------------DKKKSETGTLTHLDSDAQARKI
                                                      *:              ::  . :   : :::*      

Manduca sexta                   -------------------SLMSHRDN---MHSNKPLSAAAESTVHDGLDSKDEPGDFIE
Drosophila melanogaster         SDRCYNNVVNNITGIPGDVKVNSRLDEYINCGSISIPSNEYDCANADTTDIKDEPGDFIE
Tribolium castaneum             -------------------KIKK--------EPSNAVSGTINEGQNDQTDLKDEPGDFIE
                                                   .: .           .  *   :    *  * *********

Manduca sexta                   TNCHWVDCKLEFPTQDDLVKHINTDHIHASKKAFVCRWVGCSRDEKPFKAQYMLVVHMRR
Drosophila melanogaster         TNCHWRSCRIEFITQDELVKHINNDHIQTNKKAFVCRWEDCTRGEKPFKAQYMLVVHMRR
Tribolium castaneum             TNCHWKDCGTEFQTQDELVKHINNDHIHANKKSFVCRWDGCSRAEKPFKAQYMLVVHMRR
                                ***** .*  ** ***:******.***::.**:*****  *:* ****************

Manduca sexta                   HTGEKPHKCTFEGCCKAYSRLENLKTHLRSHTGEKPYTCEYPGCAKAFSNASDRAKHQNR
Drosophila melanogaster         HTGEKPHKCTFEGCFKAYSRLENLKTHLRSHTGEKPYTCEYPGCSKAFSNASDRAKHQNR
Tribolium castaneum             HTGEKPHKCTFEGCVKAYSRLENLKTHLRSHTGEKPYTCEYPGCSKAFSNASDRAKHQNR
                                ************** *****************************:***************

Manduca sexta                   THSNEYCSQKPYVCKAPGCTKRYTDPSSLRKHVKTVHGAEFYASKKHKGCSRGDDSAESG
Drosophila melanogaster         THSNE----KPYICKAPGCTKRYTDPSSLRKHVKTVHGAEFYANKKHKGLPLNDANSRLQ
Tribolium castaneum             THSNE----KPYVCKAPGCTKRYTDPSSLRKHVKTVHGADFYANKKHKGIDGGSEDG---
                                *****    ***:**************************:***.*****    . ..   

Manduca sexta                   ---------GGGAGSSPRSEEGGVPGTARGHASSASVKSESPASPLPHGL----HTPAHL
Drosophila melanogaster         QNNSRHNLQEHNIDSSPCSEDSHLGKML--GTSSPSIKSESDISSSNHHLVNGVRASDSL
Tribolium castaneum             ---------AAGLDSSPRSEDMQSHKTA--SLSSPSIKSESDVNSPGHQQQGSPLGATQL
                                              *** **:           ** *:****  .   *           *

Manduca sexta                   SAQCGGELD---FGGAALGGFGDDNGAPYFRLDGEVEQEVVGEVGQLPLMLRAMVAIGEP
Drosophila melanogaster         LTYSPDDLAEN---------LNLDDGWNCD---------DDVDVADLPIVLRAMVNIGNG
Tribolium castaneum             AGGCNDDFPGDMTTGVPRNGPMDDPAWPYDA--------EDLEIDDLPVVLRAMVGGGVA
                                   .  ::               * .                :: :**::*****  * 
```

```
Manduca sexta                   RAHHHTP--------RFGNK----------------------MGVGR---RSSDISRKS-
Drosophila melanogaster         NASASTIGGSVLARQRFRGRLQTKGINSSTIMLCNIPESNRTFGISELNQRITELKMEPG
Tribolium castaneum             VSE-------RNPRTRMKPRLQTKPPS-----NIMSPAQRKNIGITDLNRRINDLKMEGT
                                 :             *:  :                      :*:     * .::. :

Manduca sexta                   ----------------------------------SQASVVSGAVT---------------
Drosophila melanogaster         TDAEIKIPKLPNTTIGGYTEDPLQNQTSFRNTVSNKQGTVSGSIQGQFRRDSQNSTASTY
Tribolium castaneum             VTAQNKT--------------------QFTEL--QQRLQPPGNTQAQIRRD-SNSTVSSY
                                                                  .:     *

Manduca sexta                   -----------------GAAVAGPQRLVSQHQTVYDQLSPDSSRRSSQVSCVGYA-----
Drosophila melanogaster         YGSMQSRRSSQSSQVS-SIPTMRPNPSCNSTASFYDPISPGCSRRSSQMSNGANCNSFTS
Tribolium castaneum             YGSMRSADMSRKSSLASQASSMRPG---MAPGSFYDPISAGSSRRSSQLSTATTG-----
                                                       *        :.** :*  .******:*

Manduca sexta                   -------------------------------------PAPSSALAAVQAVRTSQGNQAVL
Drosophila melanogaster         TSGLPVLNKESNKSLNACINKPNIGVQGVGIYNSSLPPPPSSHLIATNLKRLQRKDS-E-
Tribolium castaneum             --------------------------------GTCIPPSPPSHLLAGQLQRLQNNPN-K-
                                                                     * * * * * :  * ..  .

Manduca sexta                   LRGVTCSEVR--AEELALE---------LDPNVQVKEEARRLSEQSNLSD-QAQTYQPYP
Drosophila melanogaster         YHNFTSGRFSVPSYMHSLHIKNNKPVGENEFDKAIASNARRQTDPVPNINLDPLTNISRF
Tribolium castaneum             ITGGN-GNLVLQTQNASLQQTALQQT-WMSSNTTASTDARRMSEPCHTLT-------DRK
                                    . ...   :   :*.          . :     :*** ::

Manduca sexta                   GDAPFPFKTESDQET----VTYKESRSNSTNTV----VITTAQVHHPNQEVNLEQVQVAE
Drosophila melanogaster         STTPHSFDINVGKTNNIASSINKDNLRKDLFTVSIKADMAMTSDQHPNERINLDE--V--
Tribolium castaneum             SPPPRPA---------------------SVTLSPLKGTVSSSELHPNQAVVLDE--VGE
                                .  *                           *:      . :.  ***: : *::  *

Manduca sexta                   GEMVENKLVIPDEMMQYLNQSILATESVTPAKTD-SNQPEPENNDKDSATKDNASEIKNT
Drosophila melanogaster         -----EELILPDEMLQYLNLVKDDTNHLEKE-----------------------------
Tribolium castaneum             GEMVENKLVIPDEMVHYLNQVADNQNNDFSTAMSWSQKPIPSP-----------------
                                     ::*::****::***      :

Manduca sexta                   NNSRDKISDVATSDDSLLKNLGAIGSDLNISDIQVDLRSLDVSMSGNSGSLLAAKTPDDK
Drosophila melanogaster         --------------------HQAVPVGSNVSETIASN-----------------------
Tribolium castaneum             --------------------SQTLPSPSSLSHILPSP-----------------------
                                                      ::    .:*.   .

Manduca sexta                   STPLQEQVIPEVTAEKEKEFKPVTSAVTTNPLQSLQTMTANQTEQSNRIRVSLPQKQNSA
Drosophila melanogaster         ------------------------------------------------------------
Tribolium castaneum             ------------------------------------------------------------

Manduca sexta                   SPKTVISQNVLSPQNLPHSMLSPQSLPHSAMSPQSIMSPHNIPHTVM-SPPSIYNVMS-P
Drosophila melanogaster         -----HYR---EQSNIY-------YTN----------------KQILT-PPSNVDIQPNT
Tribolium castaneum             -----PSTNVLSPGNLNQMMPSPSNQA----------------NQMMPSPTSNMM-MPSP
                                           .  *:                           . ::  * *

Manduca sexta                   QSVMSVMSPQQNAMSPQSMQSLMSPQM-PNQMMMSPRHNNIGSPMSQNMASPMVNMASPM
Drosophila melanogaster         TK-FT-------------------VQDKFA--MTAV-----GGSFS-QR---EL------
Tribolium castaneum             GNQVTQMMPSPGNQLNQMM---PSPSSNFNQIMPSP-----SSNFG-AIQNPMLSPAPSM
                                 . .:                    .      * :      .. :.       :

Manduca sexta                   TQNIASPMSHGMPSPMHPGLQSPMRSQNVQQYPVMQQYNQNQMPMMHPMNQNQNMVYNNQ
Drosophila melanogaster         ---------------------------S---------------TLAVPNEHGHAKCESFH
Tribolium castaneum             TQMVPSPMSARCHS-----NQMPMQSPNAQVVPQ---------NCMMPHNQNMMHCYNNR
                                                           .                   * ::      . :

Manduca sexta                   MMNYQQMNYPNQSNQMHPLQMSRSSMM-----------SVDNSANMSRGAMNNYCEPQNQ
Drosophila melanogaster         -------------------HQSQKYMNTDIGSKQQSALPSAHQRQTEKSNYNQIIDSSMT
Tribolium castaneum             PMCW-----DNN--QNRPCQANGGYMGNCATHYHNGYVPNG-CASNKPMNYNTYQNPAYN
                                                   : .   *                  . .    *   :

Manduca sexta                   CPPMQN-PPYNPNMQYPQPPPYNSVVNNTNVMGPPPPKNNHQYNQTMMNNNQYYNHQRPY
Drosophila melanogaster         SLPELNVDSIYP-----------------------------------------------R
Tribolium castaneum             CLPSLNEPLPSPAMAAPAPA---------DL------MNQPHYNQTCYQA--NYNQCVPQ
                                . *  *     *
```

```
Manduca sexta                   NQWDYPGNQFNKHNMQKSMQNSVNMSTGSQKPINGTRQMNCNQIKTDQTDCSMNSLRSQN
Drosophila melanogaster         N----------------ETENIF------KVHGDHDNEIQCGIISQSQMSPSTNL--NND
Tribolium castaneum             NNCNCNCRN-------PPHKCYH------QCSRGGNAEIQCKDISQSQMSPGVVA--N--
                                *                  :         :       :::*  *. .* . .     .

Manduca sexta                   NQTSEVQVWDISQSQIE------ASNGRKKNQNNTMRQETYQRTLEYVENCENWKSSEMV
Drosophila melanogaster         GQFSTVNMQPITTSKLFPPEPQKIVCDTQASNTSVMHLDTYQRTLEYVQSCQNWMETNNT
Tribolium castaneum             -----------------------------GVQPLGMRQDTYQRTLEYVQNCQSWVSNTEM
                                                               :   *: :*********:.*:.* ..

Manduca sexta                   SSST------HP--------------LQGGDNMVVNDLQTSLSSFYEENQYLQMIQ
Drosophila melanogaster         STNQIQSLPGMPVNNTLFPDVSSSTHPYHGTNMVINDMTTSLTSLLEENRYLQMMQ
Tribolium castaneum             VTSTTT----HPL---------AKCGDGTSSNMVVNDMTSSLSSLLEENRYLQLIQ
                                 :.        *                 . ***:**: :**:*: ***:***::*
```

Key:

Zinc finger double domain
Zinc finger C2H2 domain
Peptide corresponding to candidate target site

Ditrysian Ci peptide alignments

```
Hme-Ci         M-------------------------------------------------------FGSP
Pca-Ci         --------------------------------------------------------FGSR
Dpl-Ci         MPDRESVGGPGTGSGGFLPLQFPSAFAAFHASTPPGAATTAMHHATHYHHHAQLAAAAAA
Pae-Ci         ------------------------------------------------------------
Mse-Ci         ------------------------------------------------------------
Bmo-Ci         ------------------------------------------------------------
Pxy-Ci         ------------------------------------------------------------
Coh-Ci         ------------------------------------------------------------

Hme-Ci         AARQTDHMQYFGRFSES------------ARLLIGLEYLSAARSLHPELHAGSTLASQDF
Pca-Ci         AARQTDHMQYFRRFS--------------ESLFTGLEYLSAARSLHPELHAGSTLASQDF
Dpl-Ci         AAGATSELSYLAALHPAYRPVPYDHPLYGANTLRGLEYLSAARSLHPELHAGSTLASQDF
Pae-Ci         ----------------------------------GLEYLSAARSLHPELHAGSTLASQEF
Mse-Ci         ----------------------------------GLEYLSAARSLHPELHAGSTLASQDF
Bmo-Ci         ----------------------------------GLEYLSAARSLHPELHAGSTLASQDF
Pxy-Ci         ----------------------------------GLEYLSAARSLHPELHAGSTLASQDF
Coh-Ci         ----------------------------------GLEYLNAARSLHPELHVGSTLASQEF

Hme-Ci         QFSLD------------GSRIASPNRLRLSGGAIGASANRKRAVSWSPYSAESLDLAAVI
Pca-Ci         QLSLEGDFTFNIFGIFSGSRIASPNRLRLSGGAIGASANRKRAVSWSPYSAESLDLAAVI
Dpl-Ci         QLSLE------------GSRIASPNRLRLSGGAIGASANRKRAVSWSPYSAESLDLAAVI
Pae-Ci         QLSLEG----------SGSRIASQNRLRLSGGAISASANRKRAVSWSPYSAESLDLAAVI
Mse-Ci         QLSLDGKKHS------SGSRIASPTRLRLSGGAIGASANRKRAVSWSPYSAESLDLAAVI
Bmo-Ci         QLSLDG-----------GSRIASPTRLRLSGGAIGASANRKRAVSWSPYSAESLDLAAVI
Pxy-Ci         QLSLEG-----------GSRIASPTRLRLSGGAIGASVNRKRAVSWSPYSAESLDLAAVI
Coh-Ci         QLSLEGSFETSL----PGSRIASPTRLRLSGGALSASVNRKRAVSWSPYSAESLDLAAVI

Hme-Ci         RASPASLAVRAPSAASTGSYGHLSAGAISPALSLSHASLAQQLLARGGVVGGSGVLPAGV
Pca-Ci         RASPASLAVRAPSAASTGSYGHLSAG------------------------GGSGVLPGGV
Dpl-Ci         RASPASLAVRAPSAASTGSYGHLSAGAISPALSLSHASLAQQLLARGG-VGGSSVLSGGV
Pae-Ci         RASPASLAVRAPSAAEISSFFFSASTALN-------------------------------
Mse-Ci         RASPASLAVRAPSAASTGSYGHLSAGAISPALSLSHASLAQQLLARGGVVGSSGVLAGGV
Bmo-Ci         RASPASLAVRAPSAASTGSYGHLSAGAISPALSLSHASLAQQLLARGGVVGSSGVLAGGV
Pxy-Ci         RASPVSLAVRAPSAASTGSYGHLSAGAISPALSLSHASLAQQLLAR----GGSGVLGGGV
Coh-Ci         RASPVSLAVRAPSAASTGSYGHLSAGAISPALSLSHASLAQQLLARGG-VGGSGVLPAGV

Hme-Ci         LLDPAHQQAAAAAAHHAAHAHLVAGIH---RSHISSPTQLLIGAPVDVRPGLGLDGTPPQ
Pca-Ci         LLDPAHQQAAAAAAHHAAHAHLVAGIHRIYRSHISSPTQLLIGGPVDVRPGL---GTPPQ
Dpl-Ci         LLDPAHQQAAAAAAHHAAHAHLVAGIH---RSHISSPTQLLIGGPVDVRNGLGLDGTPP-
Pae-Ci         ------------------------------------------------------------
Mse-Ci         LLDPAHQQAAAAAAHHAAHAHLVAGIH---R-HISSPTQLLMGAPVDVRPGLGLDGTPPQ
Bmo-Ci         LLDPAHQQAAAAAAHHAAHAHLVAGIH---RSHISSPTQLLMG-PVDVRQGLGLDGTPPQ
Pxy-Ci         LLDPAHQQAAAAAAHHAAHAHLVAGIH--YRSHISSPTQLLMG-PVDVRPSLNLDGTPPQ
Coh-Ci         LLDPAHQQAAAAAAHHAAHAHLVAGIH---RQHISSPTQLLMG-PLDVKPPYGLDGAGPQ

Hme-Ci         HMQQ-PPQQPEITSVMEAD--------RKSPPGLISHRD---INKPLSAAAESTVHDGLD
Pca-Ci         HMQQ-PQQQPEITSVMEAD-------RRKSPPGLSAHRENMHSNKPLSAAAESTVHDGLD
Dpl-Ci         HMQQ-PPQQPEITSVMEADSASTALTQRKSPQVLVSHRDNMHGNKPLSAAAESTVHDGLD
Pae-Ci         --------------------------QRKSPQVLMSHRDNMNSNKPLSAAAESTVHDGLD
Mse-Ci         HLQQ-PPQQSEVTSIMEAD-------RRKSPQSLMSHRDNMHSNKPLSAAAESTVHDGLD
Bmo-Ci         HLQQ-PSQQPEITSVMEADSA-TGLMQRKSPQGILTHRDNV-SNKPLSAAAESTVHDGLD
Pxy-Ci         HLQQ-PPQQPEITSIMEAD-------RRKSPQSLMSHRESMHSNKPLSAAAESTVHDGLD
Coh-Ci         HLQQPPPQQHEVTSIMEAD-------RRKSPQRL--------GNKPLSAAAESTVHDGLD

Hme-Ci         SKDEPGDFIEQ------VDCKLEFPTQDDLVKHINTDHIHASKKAFVCRWVGCSRDEKPF
Pca-Ci         SKDEPGDFIEQ------VDCKLEFPTQDDLVKHINTDHIHASKKAFVCRWVGCSRDEKPF
Dpl-Ci         SKDEPGDFIETNCHW--VDCKLEFPTQDDLVKHINTDHIHASKKAFVCRWVGCSRDEKPF
Pae-Ci         SKDEPGDFIETNCHWDQVDCKLEFPTQDDLVKHINTDHIHASKKAFVCRWVGCSRDEKPF
Mse-Ci         SKDEPGDFIETNCHW--VDCKLEFPTQDDLVKHINTDHIHASKKAFVCRWVGCSRDEKPF
Bmo-Ci         SKDEPGDFIETNCHW--VDCKLEFPTQDDLVKHINTDHIHASKKAFVCRWVGCSRDEKPF
Pxy-Ci         CKDEPGDFIETNCHW--VDCKLEFPTQDDLVKHINTDHIHASKKAFVCRWVGCSRDEKPF
Coh-Ci         CKDEPGDFIEQ------VDCKLEFPTQDDLVKHINTDHIHASKKAFVCRWVGCSRDEKPF
```

```
Hme-Ci        KAQYMLVVHMRRHTGEKPHKCTFEGCCKAYSRLENLKTHLRSHTGEKPYTCEYPGCAKAF
Pca-Ci        KAQYMLVVHMRRHTGEKPHKCTFEGCCKAYSRLENLKTHLRSHTGEKPYTCEYPGCAKAF
Dpl-Ci        KAQYMLVVHMRRHTGEKPHKCTFEGCCKAYSRLENLKTHLRSHTGEKPYTCEYPGCAKAF
Pae-Ci        KAQYMLVVHMRRHTGEKPHKCTFEGCCKAYSRLENLKTHLRSHTGEKPYTCEYPGCAKAF
Mse-Ci        KAQYMLVVHMRRHTGEKPHKCTFEGCCKAYSRLENLKTHLRSHTGEKPYTCEYPGCAKAF
Bmo-Ci        KAQYMLVVHMRRHTGEKPHKCTFEGCCKAYSRLENLKTHLRSHTGEKPYTCEYPGCAKAF
Pxy-Ci        KAQYMLVVHMRRHTGEKPHKCTFEGCCKAYSRLENLKTHLRSHTGEKPYTCEYPGCAKAF
Coh-Ci        KAQYMLVVHMRRHTGEKPHKCTFEGCCKAYSRLENLKTHLRSHTGEKPYTCEYPGCAKAF

Hme-Ci        SNASDRAKHQNRTHSNE----KPYVCKAPGCTKRYTDPSSLRKHVKTVHGAEFYASKKHK
Pca-Ci        SNASDRAKHQNRTHSNE---QKPYVCKAPGCTKRYTDPSSLRKHVKTVHGAEFYASKKHK
Dpl-Ci        SNASDRAKHQNRTHSNE----KPYVCKAPGCTKRYTDPSSLRKHVKTVHGAEFYASKKHK
Pae-Ci        SNASDRAKHQNRTHSNE---QKPYVCKAPGCTKRYTDPSSLRKHVKTVHGAEFYASKKHK
Mse-Ci        SNASDRAKHQNRTHSNEYCSQKPYVCKAPGCTKRYTDPSSLRKHVKTVHGAEFYASKKHK
Bmo-Ci        SNASDRAKHQNRTHSNE----KPYVCKAPGCTKRYTDPSSLRKHVKTVHGAEFYASKKHK
Pxy-Ci        SNASDRAKHQNRTHSNE---QKPYVCKAPGCTKRYTDPSSLRKHVKTVHGAEFYASKKHK
Coh-Ci        SNASDRAKHQNRTHSNE---QKPYVCKAPGCTKRYTDPSSLRKHVKTVHGPDFYASKKHK

Hme-Ci        GCSRGDDSAESGGGGAGSSPRSEEGV--PLVRGHTSSASVKSESPASPLPMGLHTNAH--
Pca-Ci        G-----------------------------------------------------------
Dpl-Ci        GCSRGDDSAESGGGCAGSSPRSEEGG--ALIRGHTSSASVKSESPASPLPL-MHTAAH--
Pae-Ci        GCSRGDDSAESGGGGAGSSPRSEEGGISMGVRGHTSSASVKSESPASPLPHGLHTPAHQV
Mse-Ci        GCSRGDDSAESGGGGAGSSPRSEEGGVPGTARGHASSASVKSESPASPLPHGLHTPAH--
Bmo-Ci        GCTRGDDSAESGGGGAGSSPRSEEGGVPPAIRGHTSSASVKSESPASPIPHGLHTSAH--
Pxy-Ci        GCNRGDDSAESGGGGAGSSPRSEEGGVPMGIRGHTSSASVKSESPASPPLNGLHISAH--
Coh-Ci        G-----------------------------------------------------------

Hme-Ci        QLSAQCGGELDFGGSGLGGFSDENGAPYFRLDGEVEQEVVGEVGQLPLMLRAMVAIGEPR
Pca-Ci        -LSAQCGGDLDFGGSGLGGFSDENGAPYFRLDGEVEQEVVGEVGQLPLMLRAMVAIGEPR
Dpl-Ci        QLSAQCGGDLDFGGSGLGGFSDEHGAPYFRLDGDVEQEVVGEVGQLPLMLRAMVAIGEPR
Pae-Ci        QLSAQCGGELDFGVSGLGGFSDENGAPYFRLDGEVNLFSLHSFILLPLMLRAMVAIGEPR
Mse-Ci        -LSAQCGGELDFGGAALGGFGDDNGAPYFRLDGEVEQEVVGEVGQLPLMLRAMVAIGEPR
Bmo-Ci        QLSAQCGGELDFGG-GLGGYRDENGLPYFRLDGEIEQEVVGEVGQLPLMLRAMVAIGEPR
Pxy-Ci        -LSAQCGGELDFGGSGLSGFNDENGAPYFRFDGEVEQEVVGEVGQLPLMLRAMVAIGEPR
Coh-Ci        -LSAHCGGELDYGGSRLGGFGDDHGGPYFPLDGEVDQEVV-EVGQ--LMLRAMVAIGEPR

Hme-Ci        AHHHAPRFGNKMGVG----------------------GRTELGGTNVAV--ELKSGLPNT
Pca-Ci        AH-----------------------------------GRTEIGGTNVTV--ELKTGHPNT
Dpl-Ci        ---HGPRFGNKMALGRLMPSVHDMG---------AVQGRTELGSTNVAV--ELKTGLPNT
Pae-Ci        AHHHMPRFGNKMGLG----------------------GRTDIGGTNVAV--ELKSGVPNT
Mse-Ci        AHHHTPRFGNKMGVGR---------------------GRTELG-TNVAV--ELKTGLPNT
Bmo-Ci        AAPHAPRLGNKMALARMPPPHADLGGGELHFRICGVTGRTELGSTNVGV--ELKTGASNT
Pxy-Ci        AHHHTPRFGNKMGL-----------------------GRTELGTTTTGVTGELKVAIPNT
Coh-Ci        VQHPAPRLNNKMG------------------------PRTDLGTTNITG--DLKVAIPNT

Hme-Ci        RRDSGISSGSSLYSARSSDISRKSSQASVVSGAVAITT----APRLVT-HATVYDQLSPD
Pca-Ci        RRDSGISSGSSLYSARSSDISRKSSQASVVSGAVATTTGIAGPQRLLSHHSTVYDQLSPD
Dpl-Ci        RRDSGISSGSSLYSARSSDISRKSSQASVVSGAVATTIGIAGPQRLASQHTNVYDQLSPD
Pae-Ci        RRDSGISSGSSLYSARSSDISRKSSQASVVSGAVVTTTGVAGQQRLVSQHTAIYDQLSPD
Mse-Ci        RRDSGISSGSSLYSARSSDISRKSSQASVVSGAV-TGAAVAGPQRLVSQHQTVYDQLSPD
Bmo-Ci        RRDSGISSGSSLYSARSSDISRKSSQASVVSGAV---VGGVGAQRLVAHHTAAYDQLSPD
Pxy-Ci        RRDSGISSGSSLYSARSSDISRKSSQASVVSGAVATTTGVAGVQRVMSHHTAVYDQLSPD
Coh-Ci        RRDSGISSGSSLYSGRSSDVSRKSSQASVISGAVATTTGVAGAQRLLSQHTPVYDQLSPD

Hme-Ci        SSRR---------SSQVSCVGYAPPPSSALAAVQAVRTSQGN-QAVLLRGVTCSEVRAEE
Pca-Ci        SSRR---------SSQVSCVGYAPPPSSALAAVQAVRTSQGN-QAVLLRGVTCSEVRAEE
Dpl-Ci        SSRRQVFIYYLYKSSQVSCVGYAPPPSSALAAVQAVRTSQGH-QAVLLRGVTCSEVRAEE
Pae-Ci        SSRR---------SSQVSCVGYAPPPSSALAAVQAVRTSQGN-QAVLLRGVTCSEVRAEE
Mse-Ci        SSRR---------SSQVSCVGYAPAPSSALAAVQAVRTSQGN-QAVLLRGVTCSEVRAEE
Bmo-Ci        SSRR---------SSQVSCAGYAPPPSSALAAVQAVRTSQGN-Q----------------
Pxy-Ci        SSRR---------SSQVSCVGYAPTPSSALAAVQAVRTSQGNQQAVLLRGVTCSEVRAEE
Coh-Ci        SSRR---------SSQVSCVGYAPTPSSALAAVQAVRNSQGN-QAVFLRGLTCSEVTAGE

Hme-Ci        LALELDPNVQIKEEARRLSEQSNLSD-QAQGYQPYPCNNDDV----GDAPFPFKTE--DD
Pca-Ci        LALELDPNVQVKEEARRLSEQSNLSD-QAQGYQPYPCNNDDV----GDAPFPFKTE--DD
Dpl-Ci        LALELDPNVQVKEEARRLSEQSNLSD-QAQSYQPYPSNNDDV----GETPFPFKTE--DD
Pae-Ci        LALELDPNVQIKEEARRLSEQSNLSD-QAQGYQPYPCNNDDVVSTPGDAPFPFKTE--DD
Mse-Ci        LALELDPNVQVKEEARRLSEQSNLSD-QAQTYQPYP----------GDAPFPFKTE--SD
```

```
Bmo-Ci        ---ELDPNVQVKEEARRLSEQSNLSD-QTQGYQPYP----------GDAPFPFKTE--CD
Pxy-Ci        LALELDPNVQVKEEARRLSEQSNVSDPAAQGYQPYPCNTDDV----GDAPFPFKTEDVDS
Coh-Ci        LTLELDPNIQVKEEARRLSEQSNTSD-H---YRPYP----------GDAPFQFKTEQLDS

Hme-Ci        HE-VNYKESRSNSTNTVVITTAQVHHPNQEVNLEQ----VAEGEMVENKLVIPDEMMQYL
Pca-Ci        HE-VNYKESRSNSTTTVVITTAQVHHPNQEVNLEQV---VAEGEMVENKLVIPDEMMQYL
Dpl-Ci        HE-IAYKESRSNSTNTVVITTAQVHHPNQEVNLEQ----VAEGEMVENKLVIPDEMMQYL
Pae-Ci        HE-ITYKESRSNSTNTVVITTAQVHHPNQEVNLEQV--IVAEGEMVENKLVIPDEMMQYL
Mse-Ci        QETVTYKESRSNSTNTVVITTAQVHHPNQEVNLEQV--QVAEGEMVENKLVIPDEMMQYL
Bmo-Ci        QETVTYKESRSNSTSTVVVTTAQIHHPNQEVNLEQ----VAEGEMVENKLVIPDEMMQYL
Pxy-Ci        HDAVSYKESRSNSTNTVVITTAQVHHPNQEVNLEQV--QVAEGEMVENKLVIPDEMMQYL
Coh-Ci        HE-VQYKDSRSNSTSTVVVTTAQIHHPNEEVNLEQVFSQVAEGEMVENKLVIPDEMMQYL

Hme-Ci        NQSILGTDSVAPAKTDSTNKKPEGKSSE--DDNPTKDILSNETQNNNNDSDKISDVATSD
Pca-Ci        NQSILGTESGAPAKTDSTNPEPAGTSEE--KDTTTKDTTNSEIQNTNNSSDKISDVATSD
Dpl-Ci        NQSILGTDSVTPAKTDSTNLKPEASK----DNDTTKDILTSETQ-TNNSSDKISDVATSD
Pae-Ci        NQSILGTDSVTPAKTESSNPDTEGVNNE--KDTTAKDILTSEIHNTNNSSDKISDVATSD
Mse-Ci        NQSILATESVTPAKTDSNQPEPE--NND--KDSATKDNAS-EIKNTNNSRDKISDVATSD
Bmo-Ci        NQSILATEAETSAKTDSANRPNTEPSLD--KDSATKDNTTNEQQNTNNSSDKISDVATSD
Pxy-Ci        NQSILGTESATPASTDPTKPPNSSDPKDTIDNSATKDKGTSD--DVNNSGDKISDVATSD
Coh-Ci        NQSILATESIVAQTIDPTNAEQNDPKRD--NKEISANKDRNEIDNENIPSEKISDVATSD

Hme-Ci        DSLLKNLGAIGSDLNISDIQVDLRSLDVSMSGNSGSLLVPKSPNEKNSPIQEQVIPEVI-
Pca-Ci        DSLLKNLGAIGSDLNISDIQVDLRSLDVSMSGNSGSLLVAKSPDDKNTPLQEQVIPEVVT
Dpl-Ci        DSLLKNLGAIGSDLNISDIQVDLRSLDVSMSGNSGSLLAAKSPDEKNPPLQEQVISEVAN
Pae-Ci        DSLLKNLGAIGSDLNISDIQVDLRSLDVSMSGNSGSLLASKSLDDKNPPLQEQVIPEVTP
Mse-Ci        DSLLKNLGAIGSDLNISDIQVDLRSLDVSMSGNSGSLLAAKTPDDKSTPLQEQVIPEVTA
Bmo-Ci        DSLLKNLGAIGSDLNISDIQVDLRSLDVSMSGNSGSLLAVKTPEDK-TPLQEQVTPEVKA
Pxy-Ci        DSLLKNLGAIGSDLNISDIPVDLRSLDVSMSGNSGSLLAAKSPDDKNPSLQEQVIPEAAT
Coh-Ci        DSLLKNLGAIGSDLNISDIPVDLRSLDVSMSGNSGSLLASKSPDEKNTSLQDQVIAEVKT

Hme-Ci        EKCEQE-YTKPPTSNVS--TNNPLQSLQTMAANQT-EQSNRMRINNP--LPQKPA--MSP
Pca-Ci        EKCEQD-FSKPPKSNAS--ANNPLQSLQTMTENQT-EQSNRMRMNNS--HTQKPA--MSP
Dpl-Ci        EKCED--YLK-PTANNA--ASNPLQSLQTMAANQT-EQSNRMRINNS--LPQKPA--ISP
Pae-Ci        EKCEQE-FSKPPTSNIV--TNNPLQSLQTMTANQT-EQSTRMRVNNP--LPQKPA--MSP
Mse-Ci        EK-EKE--FK-PVTSAV--TTNPLQSLQTMTANQT-EQSNRIRVS----LPQKQNS-ASP
Bmo-Ci        ENKERE--TK-----KS--DPNPLQSLQTMTANQT-EQSNKIRAN-----AQKQN--KTN
Pxy-Ci        DQCEQEKYVK-PVKNNTNNQNNPLQSLQSMAGNQTMDPSNRMRNNHNTIVTQNQNS-ASP
Coh-Ci        DPNEQETYTNRSDSTPN--QRNPLQSLQCMTASQN-MDKPRRGGNSS--ENQKPNSVRSP

Hme-Ci        KTVVM-------TPQTIMSP-NLVHSMLSPQSLPHSSMSPQSVRSPQHMPHGIMSPPSVY
Pca-Ci        KTIVMSPKTIVMSPQTVMSP-NLAHSMLSPQSLPHSSMSPQSVRSPQHMPHGVMSPPSIY
Dpl-Ci        KTIIM-------TPQTVMSP-NLVHSMLSPQSLPHSSMSPQSIRSPQHMPPGIMSPPSIY
Pae-Ci        KTVVM--------TQTIMSP-SLAHSMLSPQSLPHSSMSPQSIRSPQHMPQSIMSPASVY
Mse-Ci        KTVI---------SQNVLSPQNLPHSMLSPQSLPHSAMSPQSIMSPHNIPHTVMSPPSIY
Bmo-Ci        TKTIM--------SQNILSPQNLPHSMLSPQSLPHSAMSPQSVMSPHNIPQNIMSPPSAY
Pxy-Ci        KTMVMSPQTQ--IPSNVMSPQSLAHSMMSPRSLPQSAMSPQSVMSP-HMPHNVMSPPSVY
Coh-Ci        NNMVKSPQS---VPSNVMSPQMLPQNMMSPRSLPHSAMSPQSVMSPHHHSPNVMSPPNVY

Hme-Ci        NVMSPQSVMSVMSPQHNAMSPQSMQSLMSP--QMPNQ-----MTMSPRHNNIGSPISQNM
Pca-Ci        NVMSPQSVMSVMSPQHNAMSPQSMQSLLSP--QMPNQ-----MMMSPRHNNIGSPNSQNI
Dpl-Ci        NVMSPQSVMSVMSPQHNAMSPQSMPSLMSP--QMPNQ------MMSPRNNNIASPITQNM
Pae-Ci        NVMSPQSVMSVMSPQHNAMSPQSMQSLLSP--QMPNQ-----MMMSPRHNNIGSPHSQNI
Mse-Ci        NVMSPQSVMSVMSPQQNAMSPQSMQSLMSP--QMPNQ-----MMMSPRHNNIGSPMSQNM
Bmo-Ci        NVMSPQSAMSVMSPQHNAMSPQSMQSLMSP--QIPNQ-----IMLSPRHNNVSSPMSQNM
Pxy-Ci        NVMSPQSVMSVMSPQHNAMSPQSMQSLMSPQHQMQNQ-----MMMSPRHNNMGSPMSQNM
Coh-Ci        NVMSPQSAMSVMSPQHNAMSPQSMQSMMSP--QHPMQNQVQSQIMSSRNNNMVSPMSQNM

Hme-Ci        ---------------ASPMSHGMASPMHPGLQSP----VTNPMVQNMTNMTMNVQPVQNQ
Pca-Ci        ASPMMNMASPMAQNIASPMSHGMPSPMHPGLQSP--------------------------
Dpl-Ci        GSPMMNITSPLNQNIASPMSHGMPSPMHPGLQSP------NPMVQNLSNMPRNSQAIQNQ
Pae-Ci        ASPMMNMASPMTQNIASPMSHGMPSPMHPGLQSP------------------QIQMMPNN
Mse-Ci        ASPMVNMASPMTQNIASPMSHGMPSPMHPGLQSP--------------------------
Bmo-Ci        ASPMMNMASPMGQTIASPMSHGMPSPMHPGMQSPMGQGMASPMIQNMPVAMNG--PVNPN
Pxy-Ci        TSPMVNLASPMTQNMPSPMSHGMPSPMHPGMQSP--------------------------
Coh-Ci        SSPMVNMGSPMTQNVASPLSHGMPSPIHTNLQSP--------------------------

Hme-Ci        NQNMMMN----TPALSAPPYNNRQNCNNKVPNKNFNNVPNQF-QNQNYNQAPPYPAQNQ-
```

```
Pca-Ci         ------------------------------------------------------------
Dpl-Ci         NQHHMMNMNQTQQLAQQQAYNNHQNC--KLPSKNYNNVPNQY-QNQTYTQAAAYPTQNQM
Pae-Ci         HQNII-------------------------------------------------------
Mse-Ci         ------------------------------------------------------------
Bmo-Ci         LQNQMINVNQQQMALSYRPNCH------RMPARPNMPMPNQYTQNYNNNQPPQYPMQNQ-
Pxy-Ci         ------------------------------------------------------------
Coh-Ci         ------------------------------------------------------------

Hme-Ci         NFNMRNQNMQPYQMMAQFNPN--QPQMAMMPANHQNMVYNNQ-M-NY--IQPMAYMNQTN
Pca-Ci         -----------YQIMQHYNQN--HTQM--PVMANQNLVYNNQ-MINY--AQPMNYSNQSN
Dpl-Ci         NINMRHQNLQQYQMMQQFNQN--QSQIPIMPPNNSNMAYGNQ-MINY--AQHGSYTNQSN
Pae-Ci         ---YNNQMTNYVQSMNYPNQNAQMHQVQMMPNNHQNIIYNNQ-MANY--VQSMNYPNQNA
Mse-Ci         ---MRSQNVQQYPVMQQYNQN--QMPMMHPMNQNQNMVYNNQ-MMNY---QQMNYPNQSN
Bmo-Ci         NVNVHHQNVQNYQIMQSYNQN--QAPTVNQMVQNQNVHYPNQPMINY---QQMNF-NQNN
Pxy-Ci         ---MRNQNMQQYQMMQQFNQN--QMAN-MAANQNANMSYNNQQMMNY--QQQVNYPNQNA
Coh-Ci         ---------QMHPIQQQINQN------------PGNMVYNNQ-MINYQHQHQMPYPNQGN

Hme-Ci         QLHPMQMSRSSVMSVDNSGNMSRGALNSYCEQQSMCPPPAQNARYNQNMQYPQ--PPPYN
Pca-Ci         QMHPMQMSRSSMMSVDNSGNMSRGALNSYCEQQPQC-QPVQNAQYNQNMQYPQ--PPPYN
Dpl-Ci         QLHPMQISRSSMMSVDNSGNMNRGSLNGYYEQT-MS-PSAQNPQYNQNVQYPA--PPPYN
Pae-Ci         QMHPMQLSRSSVMSVDNSGNMSRGAMNSYCEQQNVC-PPMQNVQYNQNIQYPQ--PPPYN
Mse-Ci         QMHPLQMSRSSMMSVDNSANMSRGAMNNYCEPQNQC-PPMQNPPYNPNMQYPQ--PPPYN
Bmo-Ci         RAHPMQSSRSSMMSVDNSGNMTRGVMTSYCDTQHQC-PPAQTNHYNQNVQYPQ--PPPYN
Pxy-Ci         QMHPLQMSRSSVMSVDNSASIGRGTMNSYCEQQY----PQQQSQYNQPMPYPH--PPPYN
Coh-Ci         PPNPLQSSRSSGMSVDNSASMGRGTMNSYCERQY---SQMQN-QYNQNMSYPQQPPPSYN

Hme-Ci         TVVNNSNVMGPPPPKNNHQYNQAMMNNNQYYNQQRSYNQWDYPGNQFNKHN---MQKSVQ
Pca-Ci         SV-NAANVMGPPPPKNNHQYNQAMMNNNQYYSHQRSYNQWDYPGNQFNKHN---MQKSIQ
Dpl-Ci         AVNNAANVMGPPPPKNNHQYNQAMMNNNQYYNNQRSYNQWDYPGNQFNKHN---MQKSTQ
Pae-Ci         SVANSVNVMGPPPPKNNHQYNQAMMNNNQYYNHQRSYNQWDYPGNQFNKHN---MQKSGQ
Mse-Ci         SVVNNTNVMGPPPPKNNHQYNQTMMNNNQYYNHQRPYNQWDYPGNQFNKHN---MQKSMQ
Bmo-Ci         AVVNN-NVMGPPPPKNNHQYNQAMMNNNQYYNHQRPYNQWDYPGNQFNKHN---PQKSTP
Pxy-Ci         SVVNN-NVMGPPPPKNNHQYNQTMMNNNQYYNHQRPYNQWDYPGNQFNKHNGNPAQKSMQ
Coh-Ci         AAVNN-QFMGPPPPKNNHQYNQTMMNNNQYYNHQRPYSQWDYPGNQFNKHN---VQKSMQ

Hme-Ci         NSVNMSTGSQKP--NGARMSINCNQI--IKNGGEQQTDCSMNSLRSQNQ--ADVQVWDIS
Pca-Ci         NSVNMSTGSQKPV-NGTRIPMHCNQI--VKN-GEQQTDCSMNSLRSQNNQ-ADVQVWDIS
Dpl-Ci         NSVNMSTGSQKPVMNGTRISMNCNQI--MKNPG-EQTDCSMNSLRSQNNQ-ADVQVWDIS
Pae-Ci         NSVNMSTGSQKPT-NGARVPMNCNQM--AKNNGEQQADCSMNSLRSQTNQ-ADVQVWDIS
Mse-Ci         NSVNMSTGSQKPI-NGTR-QMNCNQI--KTD----QTDCSMNSLRSQNNQTSEVQVWDIS
Bmo-Ci         NSVNMSTGSQKQV-KCNRL-TNCNQI--KND----QADCSMNSLRSQNNQPSEVQVWDIS
Pxy-Ci         NSVNMSTGSQKPN-NGRTI---VNQM--MKN---AETDCSMNSLKSQGNKPSDVQVWDIS
Coh-Ci         ESVNMSTGSQKP--NGTR-PQPVNPINGLKN---DQADCSMNSLKGEGNQPSDVQVYDIS

Hme-Ci         QSQIEATNGRKKNQ------NNMRQETYQRTLEYVENCENWKSSEMVSSSTHPLQGGDNM
Pca-Ci         QSQIEATNGRKKNQ------NTMRQETYQRTLEYVENCENWKSSEMVSSSTHPLQGGDNM
Dpl-Ci         QSQIEATNGRKKNQ------NTMRQETYQRTLEYVESCENWKSSEIVSSSTHPLQGGDNM
Pae-Ci         QSQIEATNGRKKNQ------NTMRQETYQRTLEYVENCENWKSSEMVSSSTHPLQGGDNM
Mse-Ci         QSQIEASNGRKKNQ-----NNTMRQETYQRTLEYVENCENWKSSEMVSSSTHPLQGGDNM
Bmo-Ci         QSQIEANNGRRKTQ-----NNSMRQETYQRTLEYVENCENWKSSEMVSSTTHPLQ-GDNM
Pxy-Ci         QSQIEATNGRKKNQNNNNNNNVMRQETYQRTLEYVESCENWKSSEMVSSSTHPLPGADNM
Coh-Ci         QSQIEAKNGRKKNQ-----NTAMKQETYQRTLEYVENCENWKSSEMVSSSTHPLQAGDNM

Hme-Ci         VVNDLQTSLSSFYEENQYLQMIQ
Pca-Ci         VVNDLQTSLSSFYEENQYLQMIQ
Dpl-Ci         VVNDLRTSLSSFYEENQYLQMIQ
Pae-Ci         VVNDLQTSLSSFYEENQYLQMIQ
Mse-Ci         VVNDLQTSLSSFYEENQYLQMIQ
Bmo-Ci         VVNDLQTSLSSFYEENQYLQMIQ
Pxy-Ci         VVNDLQTSLTSFYEENQYLQMIQ
Coh-Ci         VVNDLQTSLSSFYEENQYLQMIQ
```

Key:

Zinc finger double domain
Zinc finger C2H2 domain
Peptide corresponding to candidate target site

Synonymous mutations introduced into Ci-CDS construct

	M	M	Q	Y	L	N	Q
Wild type:	ATG	ATG	CAA	TAT	CTT	AAC	CAA
Mutant:	ATG	ATG	CAG	TAC	CTC	AAT	CAG
